# Mental health and natural land cover: a global analysis based on random forest with geographical consideration

**DOI:** 10.1038/s41598-024-53279-7

**Published:** 2024-02-05

**Authors:** Chao Li, Shunsuke Managi

**Affiliations:** Urban Institute, Kyushu University, 744 Motooka, Nishi-Ku, Fukuoka, 819-0395 Japan

**Keywords:** Energy and society, Environmental impact

## Abstract

Natural features in living environments can help to reduce stress and improve mental health. Different land types have disproportionate impacts on mental health. However, the relationships between mental health and land cover are inconclusive. In this study, we aim to accurately fit the relationships, estimate the impacts of land cover change on mental health, and demonstrate the global spatial variability of impacts. In the analysis, we show the complex relationships between mental health and eight land types based on the random forest method and Shapley additive explanations. The accuracy of our model is 67.59%, while the accuracy of the models used in previous studies is usually no more than 20%. According to the analysis results, we estimate the average effects of eight land types. Due to their scarcity in living environments, shrubland, wetland, and bare land have larger impacts on mental health. Cropland, forest, and water could improve mental health in high-population-density areas. The impacts of urban land and grassland are mainly negative. The current land cover composition influences people’s attitudes toward a specific land type. Our research is the first study that analyzes data with geographical information by random forest and explains the results geographically. This paper provides a novel machine learning explanation method and insights to formulate better land-use policies to improve mental health.

## Introduction

Natural land cover in people’s living environments positively affects human well-being and mental health^1–6^, and this affect is mainly driven by ecosystem services^7–9^. Greenspace could improve health and well-being^10^, through reducing harm^11–13^, restoring capacities^2,14,15^, and building capacities^16,17^. However, 2.7% of the global seminatural or natural land was converted to other land types, specifically cropland and built-up area, from 1992 to 2015^18^. With a decrease in natural land cover, the estimated aggregate value of ecosystem services from 1997 to 2011 was slashed by $4.3 trillion globally and annually^8^. As the benefits of natural land cover are profound and enormous^8,9^, the effects of land cover change on mental health are critical to structure land-use plans and strategies. With continuous global development and urbanization^18^, the share of natural land cover in people’s living environments will continue to decrease. Due to the trade-off between economic development and the desire for natural land, there is an essential need to detect whether people are satisfied with the current land composition, how much alteration of land cover composition affects future mental health, and where the effects of a particular land type change are the highest.

The relationship between land cover and mental health has long been investigated^1,4,19,20^. Natural environments could reduce air pollution, noise pollution, light pollution, and extreme heat, and increase physical activity and social contact, eventually improving health and well-being by mitigating stress^4,19,21,22^. Reducing air pollution significantly benefits human well-being, especially in metropolitan areas^23^. Some findings indicate that blue-green spaces were critical to maintaining better mental status during the COVID-19 pandemic lockdown since they reduced stressor exposure^24^. The findings of a controlled laboratory study show that the impacts of natural sounds and images on stress and mental status are positive^25^. Substantial and significant evidence shows that people living in natural environments experience higher life satisfaction and happiness^2,26^. An empirical study indicates that individuals have significantly better mental health if they move to greener areas, and the effects last several years^6^. Furthermore, environmental degradation and the absence of green spaces are causal factors of mental health issues, according to a well-designed causation study^20^. Green space disproportionately affects human health among different socioeconomic and demographic groups; thus, those variables must be carefully considered^27^. On the other hand, urban land cannot be simply regarded as a negative factor. People desire more urban land to support a better life when cities become crowded^28^. Although the relationship between land cover and mental health has long been detected and discussed, the detailed impacts remain elusive. In other words, making the value of land cover change understandable and comparable is needed to achieve a sustainable society, maintain human mental health, and formulate public policies.

To probe the comparable values and impacts of land cover on mental health, quantitative land cover data play a distinct role in empirical analyses. Previous studies have used various land cover data, which describe either the share of one or several land types in a defined area^6,29–32^, or the greenery index, mainly the Normalized Difference Vegetation Index (NDVI)^33,34^. Land cover data include several land types, which are more straightforward, but temporal resolution data is further extended by at least one year^35^. While the NDVI can be obtained with the highest temporal resolution every eight days, it only depicts greenery. Although these two types of data have been widely used in previous studies, the land cover data are more suitable for the current research, which does not only concentrate on greenery. The monetary value of land covers can be estimated^36,37^. For example, residents in Germany are willing to pay 23 euro for a 1-ha increase in green urban areas within 1000 m of their houses^30^. The monetary value estimation follows the marginal substitute rate (MSR) between land cover and income. The MSR strongly relies on the marginal effects of land cover and income on well-being or mental health indicators from statistical models^37^. Most previous studies apply this method, e.g. Ref.^28–30^. The accuracy of statistical models dramatically affects the reliability of the estimated monetary value, and the assumption of the models is vital. Currently, the linear assumption is still widely employed since it is straightforward and effective.

The advantage of machine learning methods is their high accuracy. The goodness of fit in previous studies that use traditional regression methods is no more than 20%. Using the same dataset, the performance of the fine-tuned machine model still exceeds that of the traditional linear model. The relationship between mental health and land cover is mainly assumed to be linear^29,32,38^, quadratic, or logarithmic^30,31,36^. The linear relationship in this context is direct and unambiguous, suggesting a clear and definitive stance toward a specific land type. This stance can manifest in several distinct ways; i.e., it can be significantly positive, significantly negative, or not significant at all. These models are based on an uncomplicated assumption that the amounts of certain land types always have the same effect on mental health, regardless of the current land cover status. In this case, people should live in an environment with only the land type that has the most positive effect on their mental health. This is the main shortcoming of this assumption, and it is far from reality. On the other hand, the nonlinear relationship is more in line with reality. Preferences for certain land types depend on the current land cover allocation^30,31,36^. If the land cover in the living environment is too singular, it might have relatively negative impacts on residents. For example, a living environment with only urban land might lead to mental stress, while an area that includes only forest or grassland usually does not allow people to live conveniently. Thus, the fundamental idea is to build a nonlinear model. There are two types of widely used nonlinear models based on variable transformation with fundamental ordinary least squares (OLS). One assumes that the relationship between the coverage of land types, and well-being is logarithmic^31^, and the other assumes that the relationship is quadratic^30,36^. In the logarithmic relationship assumption, when certain coverage continues to increase, the effect of this land type on well-being or mental health decreases, but the direction of this attitude does not change^31^. In the quadratic relationship assumption, when the share of land cover changes, the intensity of effects on mental health will vary and may even alter the direction of the impact. Although these nonlinear assumptions are better than linear assumptions, they still have a low level of accuracy. The accuracy of machine learning methods, such as random forest, typically exceeds 60%^20,39^. A high level of accuracy means that the relationships estimated by the trained model are closer to the actual situation. To make the policies based on the analysis results reliable, we should make assumptions similar to the real world. Machine learning has fewer assumptions on the relationships than previous methods^39^. Therefore, the use of machine learning methods is valid and reasonable.

To estimate the impacts of land cover change on mental health, relatively precise relationships between land cover and mental health are desired. This study employs 100,956 observations drawn from an international survey of 37 countries and applies a nonparametric machine learning method, namely, random forest, to obtain a high-fit model. However, because the random forest model is typically model-agnostic, we employ effective tools to make the results understandable. A well-developed theory, namely, Shapley value, could fairly distribute the contribution among a group of contributors in a coalition based on game theory^40,41^. We could regard the features obtained from our survey as the contributors in a coalition, and the coalition leads to a mental health status. For example, assuming that one individual’s mental health score is 30, the forest in her/his environment might contribute 1.3 scores to her/his mental health, which could be estimated through the Shapley value method. It must be noted that if we accumulate the Shapley values of all features, the result will be equal to the value estimated by the machine learning model. This method also has disadvantages, that is, the explanations provided by Shapley values are focused on evaluating each individual case. This means that this method is not capable of producing generalized insights or conclusions. Therefore, we create a novel way, i.e., a geographically weighted connection, to link feature values with their Shapley values. Simply, we use random forest to fit the relationship between mental health and its factors, Shapley values to investigate the factors’ impacts on mental health quantitively and individually, and geographically weighted connections to generalize the explanation. According to explainable and accurate results, our research provides more information that can be used to formulate sustainable land-use policies to improve residents’ mental health.

Our study aims to investigate the relationships between land cover in individuals’ living environments and mental health, land cover’s impacts on mental health, and spatial variability of the relationships. It follows a cross-sectional observational design and involves a random sample of 100,956 participants from 37 countries. Data on mental health, demographic, and socio-economic features of the participants, including self-report mental and physical health, income, gender, job, educational background, and emotional well-being, will be collected through interviews, alongside geographical locations and land cover ratio extracted from the remote sensing dataset. A machine learning method, namely random forest, will be employed to examine associations between land cover ratio variables and mental health status while accounting for potential confounders. Shapley values are applied to compute the contribution of each land type to individuals’ mental health status, and then we use geographically weighted connections to estimate the marginal effects of each land type change. The study’s findings will be discussed in terms of land cover change implications for mental health, emphasizing the environmental role in improving mental health.

## Data and methods

### Data information

#### Survey information

Our study employs an international survey conducted by Kyushu University, Japan, from July 2015 to March 2017, covering 37 countries, including both developed and developing countries. Gallup executed the survey in each country through online and/or face-to-face methods. Gallup is the most experienced team in the global well-being survey, so the survey was able to represent each country’s demographics based on their sampling database. The investigation periods for each country were generally less than one month. The survey team created a matrix representing different age groups and genders to align with the demographics of the general population. Subsequently, they conducted recruitment and gathered responses until each cell in the matrix was filled. Moreover, to guarantee the reliability of the survey, the same questionnaires were used, while currency-related questions were based on local currencies. The population and GDP of these countries accounted for 68.58% of the global population and 82.67% of the worldwide GDP in 2017, respectively (Supplementary Material Table S2). This survey obtained self-reported individual mental health and several other demographic and socioeconomic characteristics. The total number of observations that were recorded was 100,956. However, due to a lack of geographical location or records, 95,571 observations were kept. In addition, because some individuals did not provide income information, 89,273 observations are used in the current calculations (descriptive statistics of the features shown in Supplementary Material Table S3). Except for geographical location and income information, for each respondent, all other variables of interest are completely and validly fulfilled.

The ethics review committee for Kyushu University, Japan approved all experimental protocols used for the survey, and all methods were carried out according to the relevant guidelines and regulations. All survey methods were carried out following relevant guidelines and regulations. At the beginning of the survey, respondents were informed about the survey’s aim and their rights to participate voluntarily. All respondents provided informed consent before responding to the questionnaire.

#### Mental health

**Figure 1:**
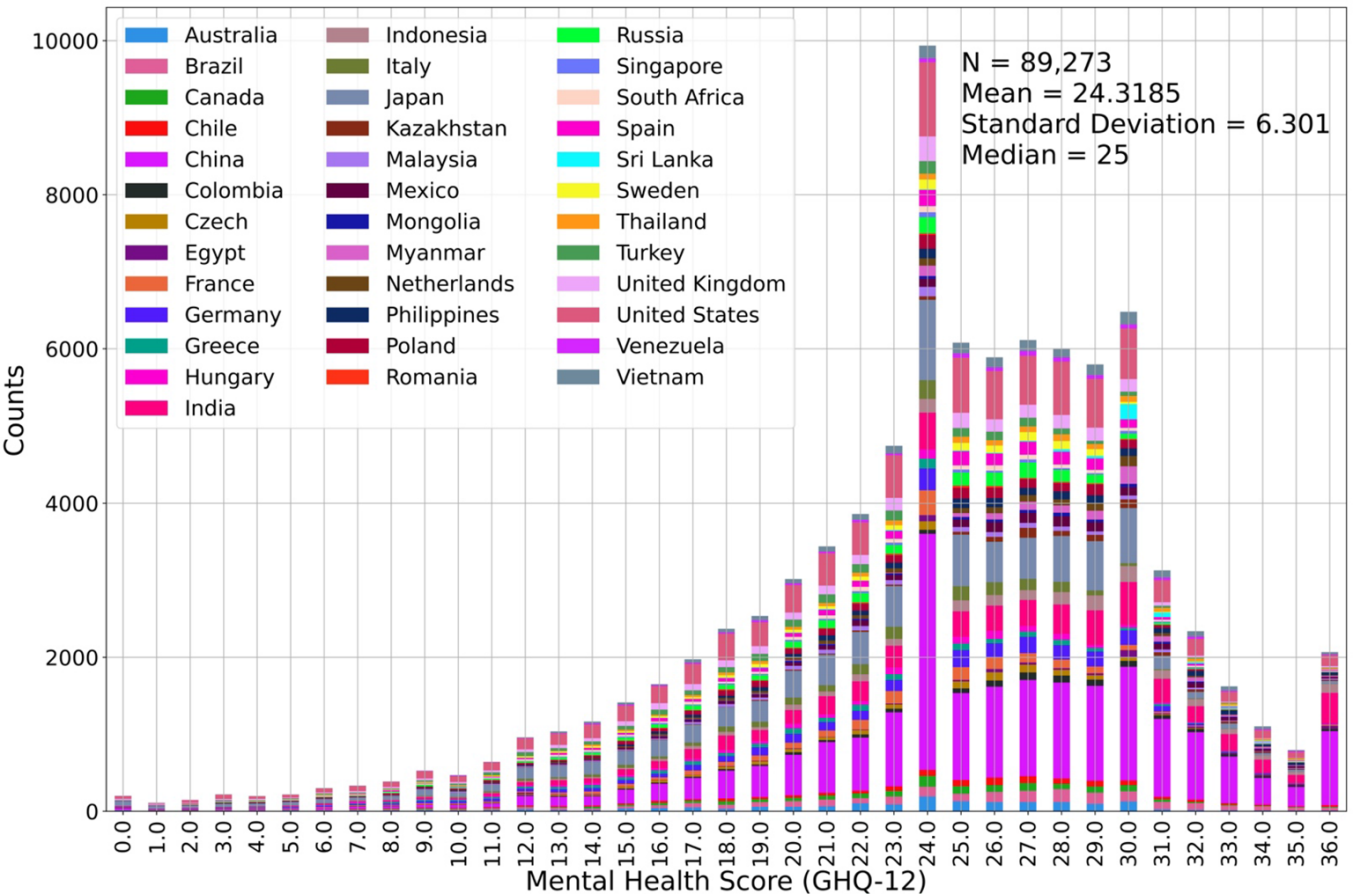
The statistical distribution of mental health assessment (the color blocks are arranged alphabetically from bottom to top according to the first letter of the country. Detailed numbers are listed in Supplementary Materials Table S1).

We include the twelve-item General Health Questionnaire (GHQ-12) in the survey to assess individual mental health. The GHQ-12 is a widely used self-report tool designed to evaluate an individual’s mental health and psychological well-being, commonly employed in clinical and research contexts^42–44^. The GHQ-12 comprises 12 items that aim to assess an individual’s experience over a specified period using a Likert scale. These 12 items ask the respondents to answer whether they have recently “(1) been able to concentrate on whatever you are doing?”, “(2) lost much sleep over worry?”, “(3) felt that you are playing a useful part in things?”, “(4) felt capable of making decisions about things?”, “(5) felt constantly under strain?”, “(6) felt you could not overcome your difficulties?”, “(7) been able to enjoy your normal day-to-day activities?”, “(8) been able to face up to your problems?”, “(9) been feeling unhappy and depressed?”, “(10) been losing confidence in yourself?”, “(11) been thinking of yourself as a worthless person?”, and “(12) been feeling reasonably happy, all things considered?”. Each item of the GHQ-12 has four potential answer options, specifically, “not at all,” “no more than usual,” “rather more than usual,” and “much more than usual,” arranged from the most negative value represented by 0 to the most positive value represented by 3. For example, for the question (1), if the participant’s answer is “much more than usual,” the score of this question should be 3, because this question is positive direction, whereas for the question (2), the same answer would rate as 0, since this question is negative direction. The mental health assessment score is computed as the summed score of all 12 items. Thus, the output variable of our study is a discrete numeric variable ranging from 0 to positive. The current random forest method is designed to execute either regression or classification. The algorithm performs the classification task using the discrete output variable, assuming the output is categorical. However, adjacent scores of the mental health assessments are related; i.e., they are ordinal rather than categorical. Figure 1 illustrates the statistical distribution of the mental health assessment scores. Most people receive 24 points in the assessment, and significantly more people score between 24 and 30 points than other range. In this situation, if we were to perform the random forest classification, then the classification accuracy for the people with lower or higher scores would be extremely low due to the unbalanced output distribution. Thus, we assume that the mental health assessment score is continuous.

#### Global land cover data

For the land cover, we use remote sensing data compiled by Tsinghua University, China (http://data.ess.tsinghua.edu.cn/), because, to our knowledge, it is the dataset with the highest global resolution, at approximately 30 m. This dataset provides information on the 2017 global land cover. It classifies land cover into ten categories: cropland, forest, grassland, shrubland, wetland, water, tundra, urban land, bare land, and snow/ice^35^. We calculate the areas of each land type surrounding our survey respondents with these data. To estimate the impact of land cover in our analysis, we use the percentages of each land type within a radius of 5000 m around each respondent,following a previous study^30^. Previous theory indicates that distance and accessibility to the natural environment would influence the relationship between land cover and mental health^45^. However, in large spatial analyses, especially multi-regional studies^29–31^, using a land cover ratio within a certain distance is still acceptable, because with a higher ratio of a land type, the residents have a higher possibility to access that land type or do some activities in that land type. Eight land types are used to examine the land cover data; the tundra and snow/ice land types are rarely present within the analyzed area. After running the random forest analysis, we estimate the Shapley values of each land type. In this study, we regard urban land as artificial land cover, while other types are considered natural.

#### Other control variables

We add several other control variables because mental health status may differ according to people’s socioeconomic and demographic characteristics; these variables are age, gender, employment, educational background, the ratio between individual income and GDP per capita in the respondent’s country (RI) (RI’s computation is summarized in Supplementary Materials), emotion in the surveyed week, number of children, self-reported health, self-reported personality, and evaluation of living environment. Among these control variables, employment, educational background, and self-reported personality are categorical. We use the one-hot encoding method to convert them into a series of dummy variables. Thus, every respondent has 49 features and one output variable in the analysis. Importantly, we include emotions in the past week to illustrate the emotional well-being; these emotions are “pleasure”, “anger”, “sadness”, “enjoyment”, and “smile”. Emotional well-being is a factor of mental health^46^. The GHQ12 is considered an aggregated score of mental health. Although there are some similar aspects between emotional well-being and the GHQ-12, we investigate each emotion’s impact on mental health by employing it as an independent variable. The descriptions of the features are listed in Supplementary Materials Table S4.

### Data analysis

#### Model pre-selection

To detect influential factors on mental health and confirm the relationship between mental health and land cover, linear regression methods, such as OLS and ordered logistic regression (OLR), are widely applied, e.g., Ref.^6,28,38,47^. These studies evaluate the monetary values of land cover through OLS estimation because OLS is straightforward to explain. Additionally, the investigations that employ the OLR are theoretically more reasonable since mental health evaluation is used as a discrete variable rather than a quantitative and continuous variable in most studies^6,38^. OLR is a typical classification function based on logistic regression. However, these two models rely on linear assumptions and thus cannot directly illustrate the importance of predictors on the outcome variable. Stated another way, based on the linear assumption, a 1-unit increase in a certain land type always has the same effect on an individual’s mental health, whatever is the status quo. This is not consistent with the actual situation. Generally, when the computational complexity of the algorithm matches the complexity of the data, the fitting results are better. Linear models’ computational complexity is relatively lower, so they cannot fit the relationships with high accuracy, in a word, under-fitting. Machine learning methods with higher computational complexities, including support vector machine (SVM), tree-based boosting models, and multi-layer perceptron (MLP), are able to grasp the non-linear relationship, which is closer to real-world situations.

In the pre-selection stage, we compare several potential models, which are OLS, OLR, SVM, adaptive boosting (AdaBoost), gradient boosting model (GBM), extreme gradient boosting (XGBoost), random forest, and multi-layer perceptron (MLP). To select the highest performance model, we test all models, except MLP, with the defaulted parameters based on tenfold cross-validation. It must be noted that we built an MLP with a similar computational complexity as XGBoost, because XGBoost has the largest computational complexity. We use the widely used equation toughly estimate the computational complexity of XGBoost. Then, based on the estimation number, an MLP’s hyperparameters, including the number of hidden layers, the number of nodes in the hidden layers, and the number of training epochs, are selected. Of course, more detailed fine-tuning, feature engineering, and hyperparameter adjustment might improve the performance of the MLP. Limited by the current computing power, we are unable to do more tests. However, to some degree, the current MLP still could be a reference to be compared with other basic models. The MLP has 22 layers, wherein one input layer, 20 fully connected layers, and one output layer. The input layer has 49 input nodes. Each fully connected layer has 100 nodes. The output layer has one output node. In total, this MLP has 207,101 parameters to train. The activation function of the fully connected layers and the output layer is “ReLU”. The MLP’s adaptor is “Adam”, the batch size is 32, and we train the MLP 20 epochs. The tenfold cross-validation average accuracies of OLS, OLR, SVM, AdaBoost, GBM, random forest, XGBoost, and MLP are 42.55%, 13.43%, 33.40%, 22.62%, 46.01% 47.34%, 47.19.%, and 44.67%, respectively, as shown in Table 1. Since our task is regression, we are also interested in root mean square error (RMSE), mean square error (MSE), and mean absolute error (MAE). Among eight potential models, OLR is for classification tasks, so RMSE, MSE, and MAE are not suitable for this method. It should be explained that RMSE and MSE are sensitive to outliers. RMSE is the same as the target variable, while MSE is more impactful. MAE is another robust measure of error when there are extreme values in the analysis. The RMSEs of OLS, SVM, AdaBoost, GBM, random forest, XGBoost, and MLP are 4.77, 5.14, 5.54, 4.63, 4.57, 4.58, and 4.65, respectively. The MSEs are 22.80, 26.44, 30.71, 21.43, 20.90, 20.96, and 21.61, respectively, and the MAEs are 3.65, 3.81, 4.52, 3.51, 3.42, 3.47, and 3.55, respectively. In terms of four indices for regression, namely R^2^, RMSE, MSE, and MAE, the random forest’s performance is the best.

**Table 1:** Statistic indicators of potential models.

Model	Task	Accuracy	RMSE	MSE	MAE
OLS	Regression	42.55%	4.77	22.8	3.65
OLR	Classification	13.43%	–	–	–
SVM	Regression	33.40%	5.14	26.44	3.81
AdaBoost	Regression	22.62%	5.54	30.71	4.52
GBM	Regression	46.01%	4.63	21.43	3.51
**Random forest**	**Regression**	**47.19%**	**4.57**	**20.9**	**3.42**
XGBoost	Regression	47.01%	4.58	20.96	3.47
MLP	Regression	44.67%	4.65	21.61	3.55

Note: Information on the best model is in bold.

In terms of the survey data, the random forest is a suitable model. The basic element, decision tree, of the random forest method has no assumption about data distribution, different from OLS and OLR. In fact, some features used in our analysis are mainly binary variables such as gender, job, and educational background, while others are discrete, such as age and RI. A decision tree is based on numerous binary judgments, so it is extremely suitable for analyzing our data.

#### Random forest

The random forest method builds a barrage of decision trees in parallel and allows them to vote for the results^48^. The voting strategy for regression takes the average value of all individual predictions as the random forest prediction. Bagging and bootstrapping are performed to guarantee the accuracy and reliability of random forest^49^. Bootstrapping is the sampling technique used by random forest. First, we set the number of trees in our random forest as ${N}_{tree}$. We extract ${N}_{tree}$ samples with replacement from the original data, and the sample sizes are 2/3 of the data of the total sample. Every decision tree utilizes the bootstrapped dataset. However, at most, a predefined number of random features (${N}_{features}$) are used in a single decision tree rather than all the features. After training, the random forest model can predict the output variable by aggregating the votes from each tree. Using the bootstrapped dataset and the aggregate of votes, this process is terminologically called “bagging”. Additionally, approximately 1/3 of the total sample is left out from the training process, which is called the out-of-bag (OOB) dataset. The OOB dataset is applied to test the accuracy of the random forest model through the OOB score, which is the proportion of OOB observations correctly predicted by the trained random forest. The reliable trained models have a relatively high OOB score.

In random forest, most parts are built randomly, while only three critical parameters must be decided by the users, specifically, the minimum number of remaining observations in end leaves (${N}_{remain}$), ${N}_{tree}$ and ${N}_{features}$. First, the minimum number of observations in the end leaves decides where the split stops because our random forest follows the greedy approach. If ${N}_{remain}$ is too small, the decision tree might be too deep and too many end leaves would be generated, which could cause the model to be large and even unavailable to the computer memory. Moreover, the random forest accuracy will increase to some extent when more trees are included. However, the cost of infinitely increasing ${N}_{tree}$ is a dramatic increment of calculation power and calculating time. Additionally, when ${N}_{tree}$ exceeds a particular value, the marginal effect of increasing the number is minimal. Accordingly, considering the size of our dataset and computing ability, the number of trees is set to 1,000. Moreover, the number of features used in the decision trees, ${N}_{features}$, is another vital factor. A large ${N}_{features}$ might reduce the model’s ability to grasp the relationship, while a small ${N}_{features}$ might cause underfitting. Previous studies have indicated that roughly one third of the total number is recommended^48–50^. Thanks to our relatively sufficient computing ability of a high-performance computer, we test the most possible ${N}_{features}$ values based on tenfold cross-validation. According to the test, the goodness of fit peaks when the ${N}_{features}$ value is 11 (the hyperparameter process is summarized in Supplementary Materials Table S5). We also test several possible ${N}_{remain}$ values, including 2, 5, 10, 15, 20, 25, 30, 35, and 40, based on tenfold cross-validation. Although the results show that with the same ${N}_{features}$ and ${N}_{tree}$, a smaller ${N}_{remain}$ causes a higher cross-validation score, the improvement is limited. For example, the increase in ${N}_{remain}$ in the cross-validation score from 2 to 10 is not more than 1%. However, the disadvantage of the smaller ${N}_{remain}$ is obvious. When we build the connection between the Shapley value and the values of features locally, the limited local datasets might make the connection coefficient nonsignificant. Due to the trade-off, we set ${N}_{remain}$ as 30. In plain language, each decision tree randomly picks 11 features from the dataset, and each end leaf includes at least 30 observations.

In this study, we employ the geographical coordinates of each respondent in the fitting process. In other words, our random forest model is apt to assign geographically close respondents to the same branch. This way is more effective than employing country variable. The division of the model is the basis of geographically local dataset. The latter stages, namely random forest model explanation and the connections between observed and explanation values, are based on the locally geographical environments. In this way, we do not need to use administrative regions to reduce mental health variations among countries and regions. This method should be more valid and reasonable. Changes in mental health are geographically continuous rather than abrupt. To clarify the difference between continuous variation used in our research and abrupt change employing country variables, we provide a simple example here. Assume that there are two respondents who are completely the same living close to the national boundary, such that respondent A and B belong to two different countries, i.e., countries A and B, respectively. Although there could not be large difference between the living environments of respondents A and B, the model predictions for those two respondents might be dramatically different. In contrast, our method divides the large dataset into numerous local datasets based on geographical information. Every respondent could be included in several local datasets. Geographically, the variation in local datasets is continuous. We investigate the local connections within each local dataset. Therefore, these local connections are also geographically continuous and spatially varied, and it is not necessary to employ the country variable.

#### Variable importance

Random forest could estimate the importance of each feature on the output variable. The basic idea of importance estimation in random forest is to calculate the reduction in accuracy before and after excluding a specific feature^48^. The reduction in the accuracy of a particular feature would be higher when it is more important to successfully predict the output variable compared with other features. This reduction is similar to the partial R^2^ in the OLS algorithm. There is no need to select the features in the random forest algorithm since issues, such as multicollinearity, do not influence the accuracy of the random forest algorithm. However, multicollinearity is a fatal problem in OLS.

#### Shapley additive explanations (SHAP)

Although the accuracy of random forest is high, it is challenging to understand and explain the results^41,51,52^. Shapley additive explanations (SHAP) is an advanced approach that aims to explain the contributions of each feature locally based on theoretically optimal Shapley values^40^. To explain the contributions of features, each feature of the observation is a “player” in a game, and the prediction value is the payout. Shapley values help us fairly distribute the payout among the players^40,53^. The Shapley value of a feature value is estimated as follows:

1 $${S}_{jx}=E[\frac{1}{p!}\sum_{J}{g}^{j|\pi (J,j)}\left(x\right)]$$

where $x$ represents a specific observation of interest, $j$ represents a particular feature of interest, ${S}_{jx}$ represents the Shapley value of the feature $j$ of the observation $x$, $J$ represents a permutation of the set of indices $\left\{1, 2,\dots ,p\right\}$ corresponding to an ordering of $p$ features included in our random forest model, $\pi (J,j)$ represents the set of the indices of the features contained in $J$ before the $j$-th variable, and ${g}^{j|\pi (J,j)}(x)$ represents the estimated contribution value of feature $j$ of the observation $x$ with a specific permutation. ${g}^{j|\pi (J,j)}(x)$ is calculated as follows:

2 $${g}^{j|\pi (J,j)}\left(x\right)=E\left(f\left(X\right)|{X}^{1}={x}^{1},\dots ,{X}^{j-1}={x}^{j-1},{X}^{j}={x}^{j}\right)-E\left(f\left(X\right)|{X}^{1}={x}^{1},\dots ,{X}^{j-1}={x}^{j-1}\right)$$

where $X$ represents a matrix of random values of features, $f()$ represents our trained random forest model, $E\left(f\left(X\right)|{X}^{1}={x}^{1},\dots ,{X}^{j-1}={x}^{j-1},{X}^{j}={x}^{j}\right)$ is the expected value of the predictions of $X$, when we set ${X}^{1}={x}^{1},\dots ,{X}^{j-1}={x}^{j-1},{X}^{j}={x}^{j}$, and $E\left(f\left(X\right)|{X}^{1}={x}^{1},\dots ,{X}^{j-1}={x}^{j-1}\right)$ is the expected value of the predictions of $X$, when we set ${X}^{1}={x}^{1},\dots ,{X}^{j-1}={x}^{j-1}$. $X$ is used to complish the predictions based on the trained random forest model, $f()$. Importantly, generally, the random values are deemed to have no explanatory ability. However, the random feature values in $X$ must belong to a range of feature values and have the same numerical characteristics. Each row in $X$ could be regarded as a real individual. Therefore, in real computations, the random dataset $X$ is not randomly generated but instead randomly picked up from our dataset. In the SHAP estimation, some features would be replaced by the aimed individual’s certain feature value. Of course, if features, even a feature, are different between two rows, we could regard them as two different individuals. When a part of $X$ is replaced, it does not represent the real individuals from our survey anymore. In our analysis, we set the dataset size of $X$ as 1000, approximately 1% of the total dataset, according to the python package makers’ recommendation^41^. We must emphasize that all features’ contributions to mental health for each observation, $x$, are estimated. $X$ is simply a random matrix; it does not represent the total dataset^41,53^. A larger dataset size here would definitely increase the computation time. To estimate the Shapley values efficiently, we use 4048 random permutations of all features. Of course, more permutations lead the estimated values to the real values, but the computing time is not affordable.

#### The connection between features’ values and their SHAP values

The explanations of SHAP values are too local. One observation’s SHAP values illustrate only one individual’s particular situation and thus cannot be directly used on other observations. A SHAP value is the feature value’s contribution to each observation’s current mental health status. For example, in one observation’s living environment, urban land comprises 99.60% of the total, and its SHAP value is -0.009. This individual’s living environment is monotonous and full of urban land, which might negatively affect her or his mental health. For another observation, urban land comprises 73.98% of the total, and its SHAP value is 0.012. The impacts of a certain feature on an individual’s mental health might be associated with his or her the current status. We employ linear regression to probe the relationship between a feature value and its contribution to mental health. However, since this research is global, a huge spatial extent makes the globally unified relationship suspicious. Estimating the relationship locally is more rational. Based on the local regression, although the relationships are locally linear, they are globally nonlinear.

Building a series of local datasets is the critical aspect. In the model training process, the location information is also included, which is the longitude and latitude of the observation. Some decision trees pick up these features. These trees divide the global extent into several zones. The observation location belongs to zones divided by different trees. Thus, we obtain a bag of boundaries. The maximums of the boundaries in each direction are regarded as the dividing lines. Every observation is surrounded by a rectangle of dividing lines, and others within one observation’s zones are considered neighbors. The neighboring zones differ by location. Every respondent has her or his neighbor zone; thus, we obtain 89,273 neighbor zones, which are geographically local. The local relationship is estimated based on one observation and others located in its neighboring zone; thus, the relationship coefficients also spatially vary. The estimation process is as follows:

3 $${S}_{jx}={\alpha }_{jx}{X}_{x}^{j}+{\beta }_{jx}$$

where ${\alpha }_{jx}$ and ${\beta }_{jx}$ are the slope and the intercept of the local relationship between feature $j$’s value and its SHAP value based on $x$’s neighbor zone, ${X}_{x}^{j}$ is a vector of the feature $j$’s values in $x$’s neighbor zone, and ${S}_{jx}$ is a vector of the SHAP values corresponding to ${X}_{x}^{j}$. According to the local relationship coefficient, we could interpret the marginal contribution of an increase in a certain feature to mental health. To improve the geographical continuity of the relationship and emphasize the difference between each point in the same neighboring zone, we add geographical weights to the coefficient estimation process. We calculate the local geographical weight vector as geographically weighted regression methods^23,54^ as follows:

4 $${{\varvec{W}}}_{x}= {\left[1-{\left({{\varvec{d}}}_{x}/{h}_{x}\right)}^{2}\right]}^{2}$$

where ${{\varvec{W}}}_{x}$ is the geographical weight vector of the elements in $x$’s neighbor zone, ${{\varvec{d}}}_{x}$ is a vector of distances between $x$ and the elements in $x$’s neighbor zone, and ${h}_{x}$ is the farthest distance of the distance vector ${{\varvec{d}}}_{x}$. According to this equation, the weights of the elements with the furthest distance in $x$’s neighbor zone are always zero, while the aim observation $x$ always has the largest weight, 1, in the regression. With the geographical weight vector, the local coefficient is estimated as follows:

5 $${Coef}_{jx}={({{X}_{x}^{j}}^{T}{{\varvec{W}}}_{x}{X}_{x}^{j})}^{-1}{{X}_{x}^{j}}^{T}{{\varvec{W}}}_{x}{S}_{jx}$$

where ${Coef}_{jx}$ is the estimated local coefficient, including ${\alpha }_{jx}$ and ${\beta }_{jx}$. Because we have 89,273 geographically local datasets, we eventually obtain 89,273 sets of local coefficients, which spatially vary.

#### Monetary values of land cover

To make the impacts of land cover change on mental health understandable and comparable, we estimate the monetary values of land cover. This method is friendly to the public because it is free of considerable background knowledge. We take the marginal substitution rate (MSR) of land cover and income as the monetary values, and it is estimated as follows:

6 $${MSR}_{jx}=\frac{{\alpha }_{jx}}{{\alpha }_{INCx}}$$

where ${MSR}_{jx}$ is the MSR of feature $j$ in observation $x$’s location, and ${\alpha }_{INCx}$ is the local relationship coefficient between the income value and its SHAP value based on the observations in $x$’s neighbor zone. In this equation, we require either the coefficients ${\alpha }_{jx}$ and ${\alpha }_{INCx}$ to be significant (*p* value < 0.1), or the MSR to be set to zero.

7 $${MV}_{jx}={MSR}_{jx}\times {GDPPC}_{x}$$

where ${MV}_{jx}$ is the monetary value of feature $j$ in observation $x$’s location, and ${GDPPC}_{x}$ is the GDP per capita of respondent $x$’s country in the surveyed year. Based on these equations, the monetary values can be explained by how much income changes equal a 1% increase in a specific land cover.

#### Analysis roadmap

**Figure 2:**
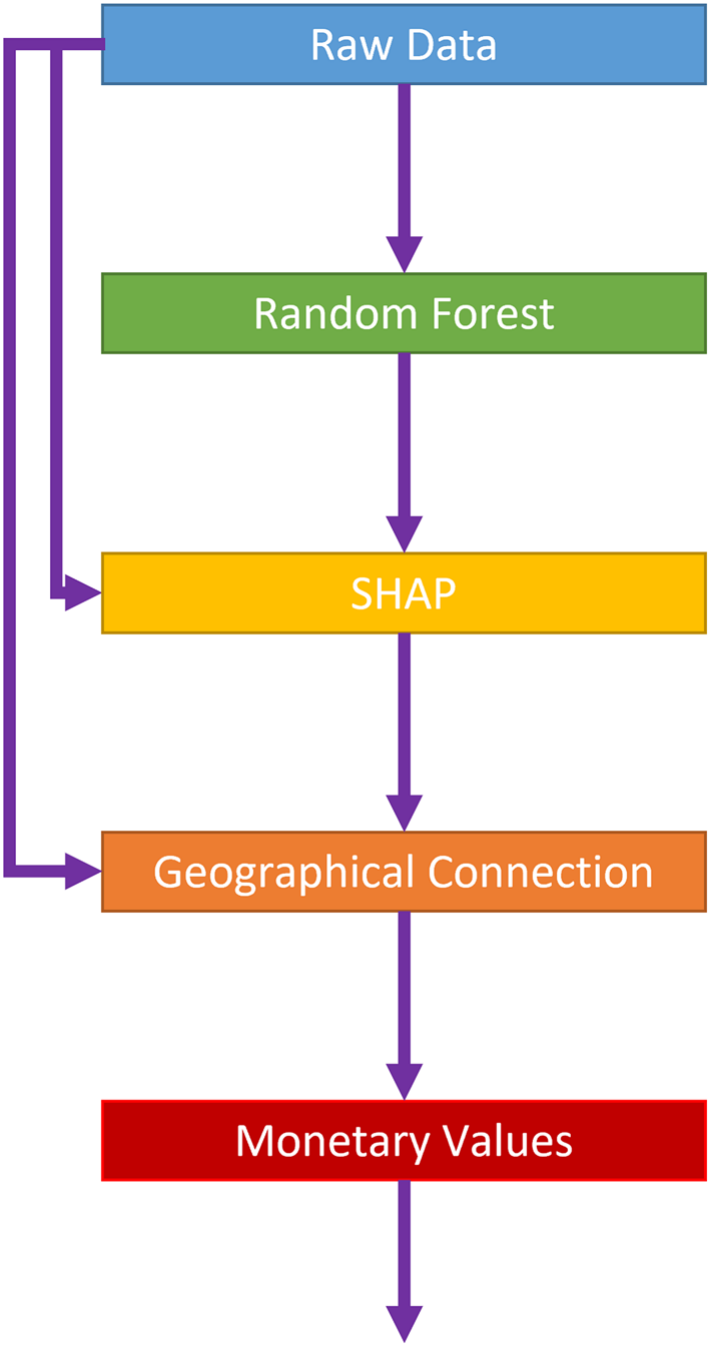
Analysis roadmap.

Figure 2 demonstrates our analysis roadmap from raw data to monetary values. First, we use the raw data to train a high-accuracy random forest. The random forest model is nonparametric, which means that the contribution of each variable is not straightforward. In this way, we take the second step to estimate the contribution of each variable value to mental health by using SHAP values. Importantly, SHAP values depict the contribution of current values of variables to mental health individually. A positive SHAP value indicates that the current variable values positively contribute to mental health, and vice versa. In other words, in the current study, we regard SHAP values as highlighting people’s attitude toward their current status. However, we do not know how variations in the current values affect SHAP values. Hence, we should use some method to connect the SHAP values with real values. Since this study covers the whole world, a statistic global analysis might lead to a biased relationship. Therefore, in the third step, we employ geographically weighted regression and local datasets to investigate the local coefficients individually. In fact, for each respondent, the coefficients of relationships between values of the variables of interest and their contribution to mental health can be spatially varied. For an individual respondent, a positive coefficient for a variable indicates that as the variable increases, its contribution to mental health also increases. Simply, the local coefficients of geographical connection represent the people’s attitude toward variations in variables of interest, and they are not directly related to the current values. In the fourth step, we use the local coefficients of each respondent to calculate monetary values. These monetary values can also differ among the respondents. They are not directly affected by the current variable values. These monetary values help make people’s attitudes toward the variation in variables easily understandable.

## Results

**Figure 3:**
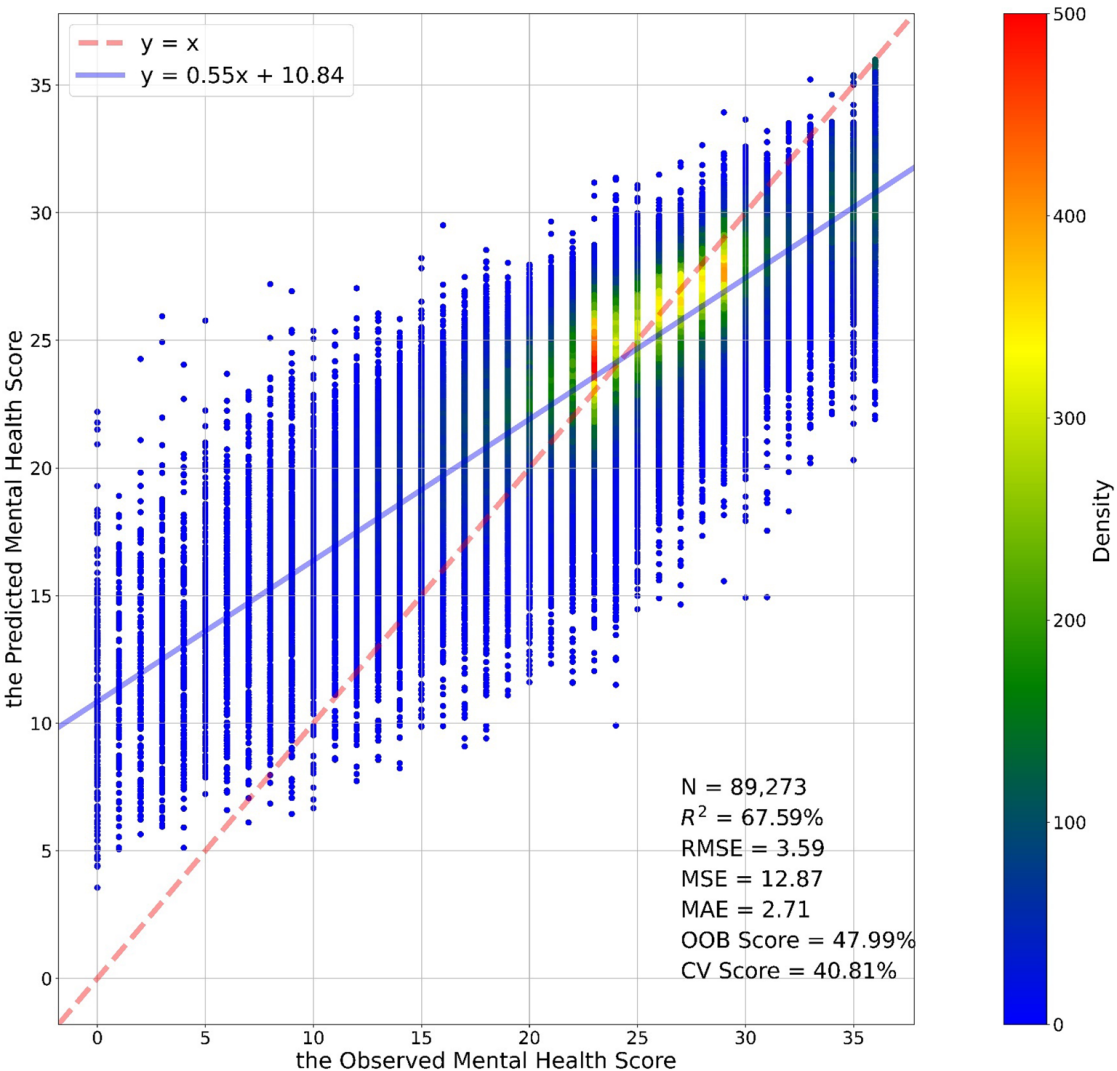
The density plots between the measured and predicted mental health score (the red dashed line is the 1:1 line. The blue line is the regression line.)

In this study, the trained random forest model employs 1000 trees. At most, 11 features are randomly chosen in the bootstrapped datasets to train each tree. Every end leaf must have at least 30 observations. The accuracy of the random forest model is 67.59%, whereas the accuracy of the OLS model is only 42.66%. Moreover, the values of the RMSE, MSE, and MAE of the random forest model are 3.59, 12.87, and 2.71, respectively, while the values of the RMSE, MSE, and MAE of the OLS model are 4.77, 22.77, and 3.65, respectively. In terms of accuracy, the random forest model in this study significantly exceeds the linear regression. The OOB score of our model is 47.99%. Additionally, the average tenfold cross-validation score of the random forest model is 40.81%, while the score of the OLS model is 38.19%.Our model is selected based on the trade-off between accuracy and explanation. Figure 3 demonstrates the relationship between predicted and measured mental health scores. The slope of the fit line between the predicted and measured mental health scores is lower than 1. Random forest rarely exactly predicts extreme values, e.g., the 0 and 36 values at the extreme ends of the score range for the GHQ-12. Put another way, random forest’s prediction is closer to the mean value of the output variable. As shown in Fig. 1, extreme values are rare; thus, the status of the random forest model is acceptable.

**Figure 4:**
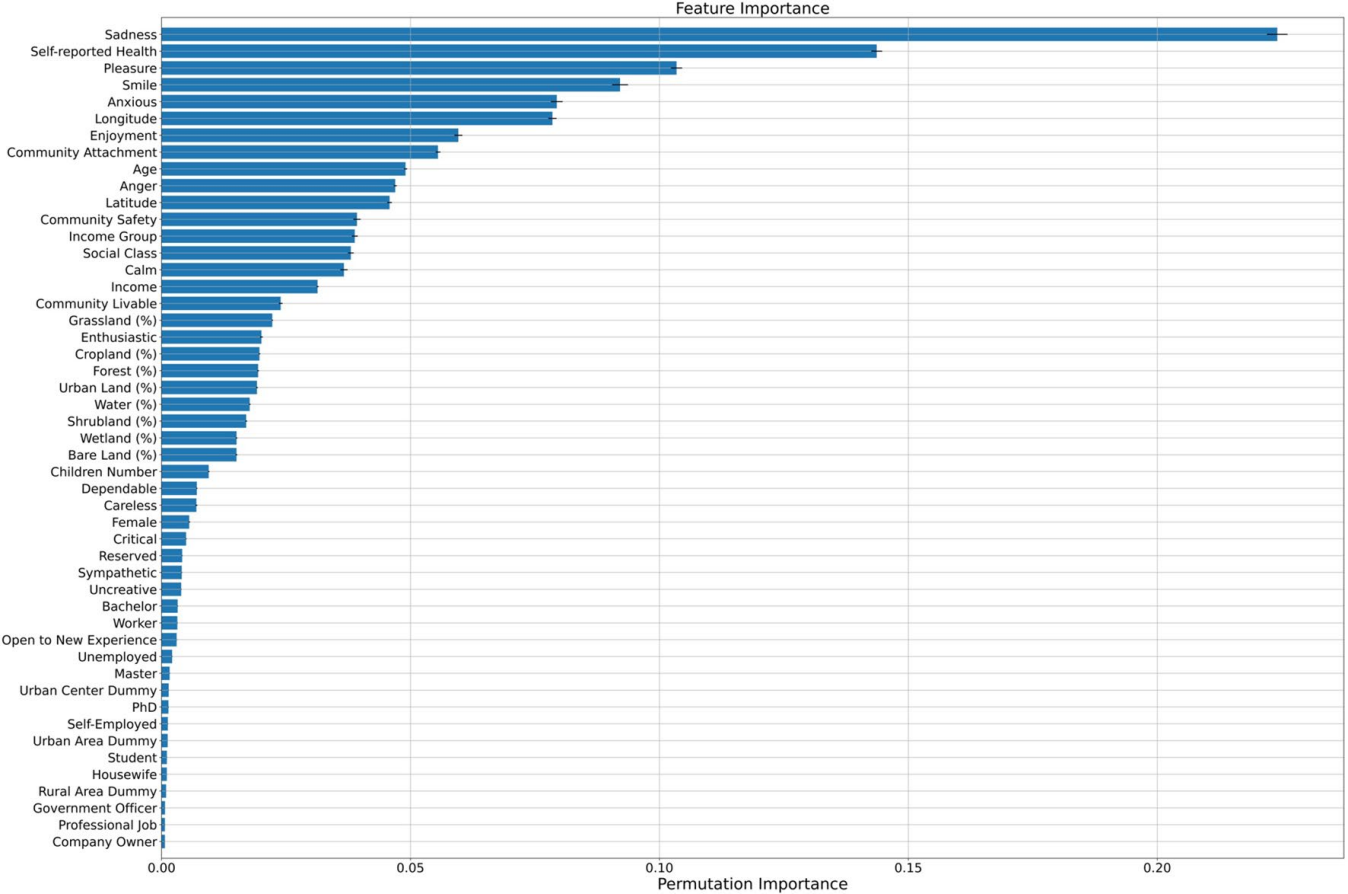
Feature importance.

Figure 4 demonstrates the importance of each feature. Emotions, including sadness, pleasure and smile, and self-reported health, affect mental health the most. For example, if we do not employ the feature “sadness” in the model, the accuracy will decrease by 22.41%. The income and land cover in respondents’ living environments significantly influence their mental health. The accuracy decreases by 3.13% by not including the income feature in the model. Moreover, the importance values of cropland, forest, grassland, shrubland, wetland, water, urban land, and bare land, are equal to 1.97%, 1.94%, 2.23%, 1.70%, 1.50%, 1.77%, 1.92%, and 1.51% reductions in accuracy, respectively.

**Figure 5:**
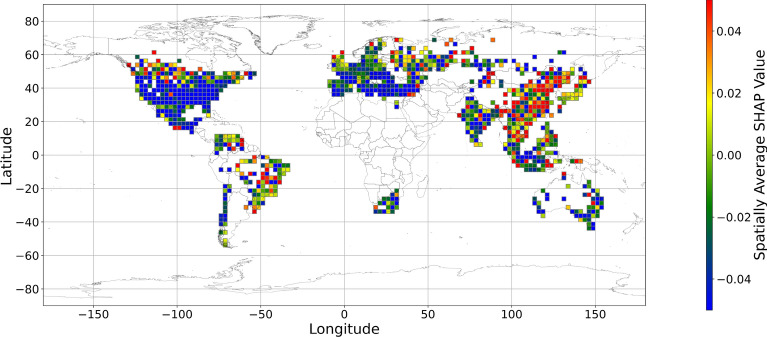
The spatially average SHAP values of income. (Note: Cell size is 2.5° × 2.5°; Map’s Shapefile is downloaded from https://hub.arcgis.com/datasets/esri::world-countries-generalized/explore; We use Python 3.9.16 to plot https://www.python.org/downloads/release/python-3916/).

**Figure 6:**
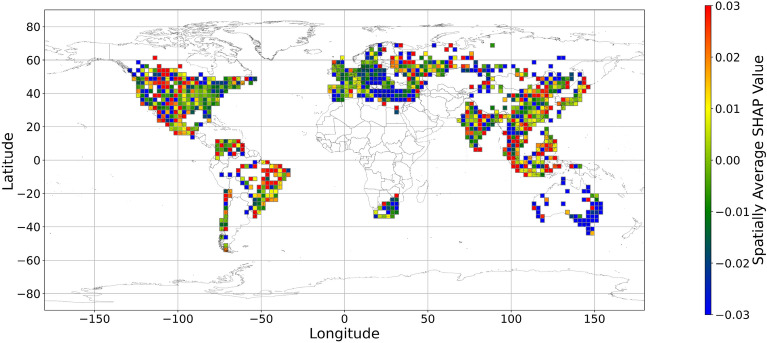
The spatially average SHAP values of cropland (note: Cell size is 2.5° × 2.5°; Map’s Shapefile is downloaded from https://hub.arcgis.com/datasets/esri::world-countries-generalized/explore; We use Python 3.9.16 to plot https://www.python.org/downloads/release/python-3916/).

**Figure 7:**
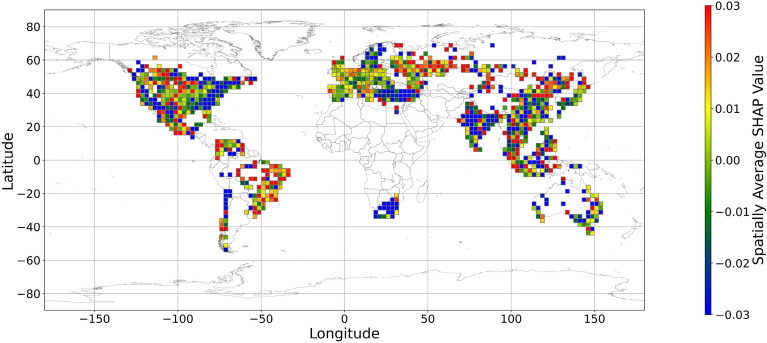
The spatially average SHAP values of forest (Note: Cell size is 2.5° × 2.5°; Map’s Shapefile is downloaded from https://hub.arcgis.com/datasets/esri::world-countries-generalized/explore; We use Python 3.9.16 to plot https://www.python.org/downloads/release/python-3916/).

**Figure 8:**
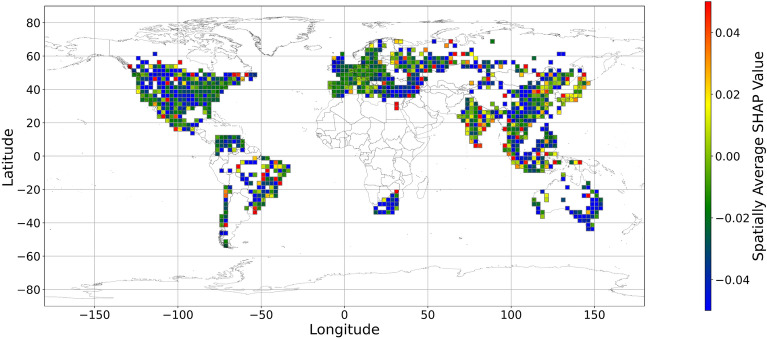
The spatially average SHAP values of grassland (Note: Cell size is 2.5° × 2.5°; Map’s Shapefile is downloaded from https://hub.arcgis.com/datasets/esri::world-countries-generalized/explore; We use Python 3.9.16 to plot https://www.python.org/downloads/release/python-3916/).

**Figure 9:**
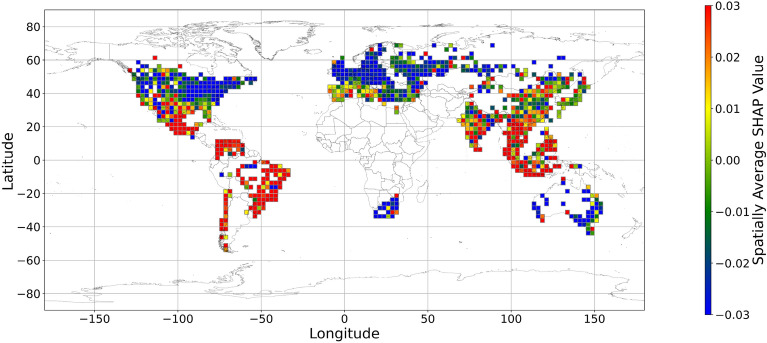
The spatially average SHAP values of shrubland (Note: Cell size is 2.5° × 2.5°; Map’s Shapefile is downloaded from https://hub.arcgis.com/datasets/esri::world-countries-generalized/explore; We use Python 3.9.16 to plot https://www.python.org/downloads/release/python-3916/).

**Figure 10:**
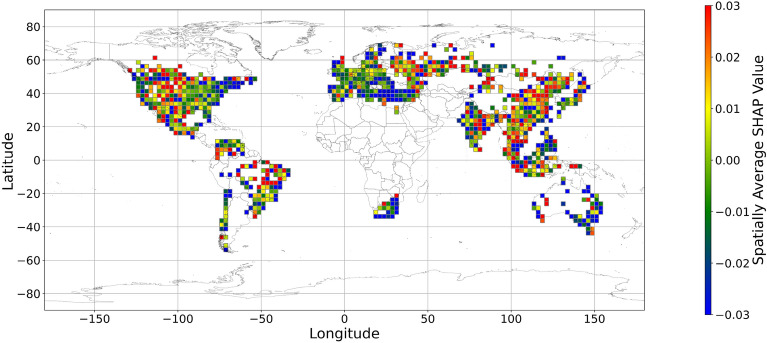
The spatially average SHAP values of water (Note: Cell size is 2.5° × 2.5°; Map’s Shapefile is downloaded from https://hub.arcgis.com/datasets/esri::world-countries-generalized/explore; We use Python 3.9.16 to plot https://www.python.org/downloads/release/python-3916/).

**Figure 11:**
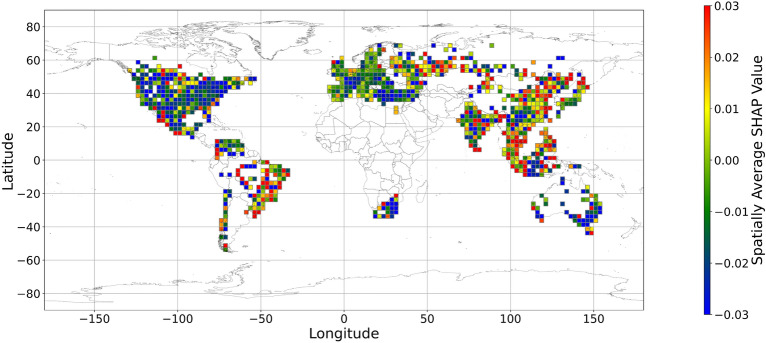
The spatially average SHAP values of wetland (Note: Cell size is 2.5° × 2.5°; Map’s Shapefile is downloaded from https://hub.arcgis.com/datasets/esri::world-countries-generalized/explore; We use Python 3.9.16 to plot https://www.python.org/downloads/release/python-3916/).

**Figure 12:**
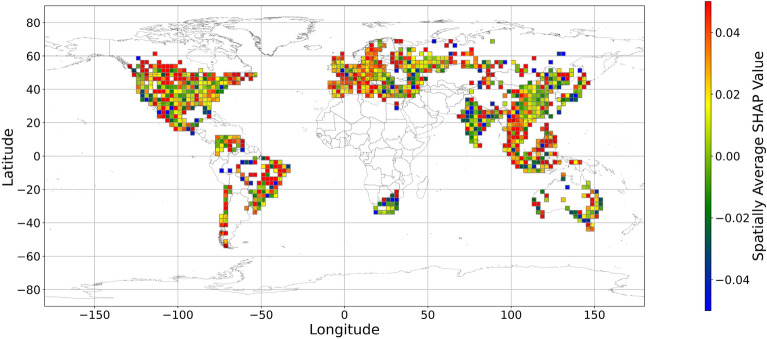
The spatially average SHAP values of urban land (Note: Cell size is 2.5° × 2.5°; Map’s Shapefile is downloaded from https://hub.arcgis.com/datasets/esri::world-countries-generalized/explore; We use Python 3.9.16 to plot https://www.python.org/downloads/release/python-3916/).

**Figure 13:**
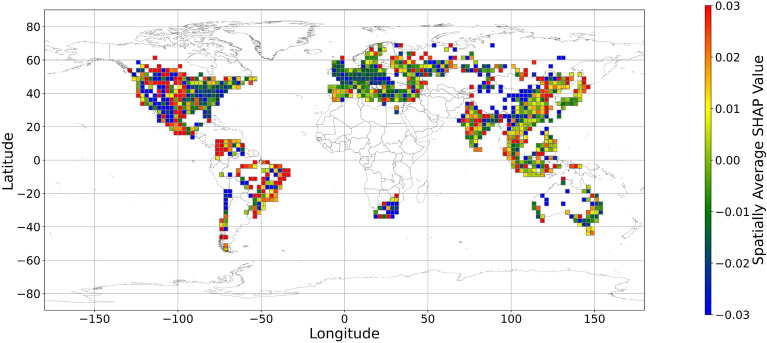
The spatially average SHAP values of bare land (Note: Cell size is 2.5° × 2.5°; Map’s Shapefile is downloaded from https://hub.arcgis.com/datasets/esri::world-countries-generalized/explore; We use Python 3.9.16 to plot https://www.python.org/downloads/release/python-3916/).

Figures 5, 6, 7, 8, 9, 10, 11, 12 and 13 illustrates nine maps of spatially average SHAP values of income and land cover features. To make the SHAP values spatial distribution readable, we use a spatially average value because the geographical scatter plots are hard to read (Supplementary Materials Fig. S4). We mean all the values in each cell with a 2.5-arc-degree side length. The observation numbers in each cell are different. Figure 5 displays the spatially average SHAP value of income. In most areas, current income features negatively contribute to mental health. A lower RI value is the main reason for negative contributions. Previous studies have indicated that increased income improves human self-evaluation and emotional well-being, although some have noted there is a threshold for further improvement^55,56^. For most people, mental health can benefit from increased income. Based on Fig. 5 and the current status of RI (Supplementary Materials Fig. S4.a), it can be inferred that income positively affects mental health. However, the SHAP value of the land cover feature represents the attitudes toward current feature values. Figures 6, 7, 8, 9, 10, 11, 12 and 13 demonstrate the SHAP values of the land cover features. Thus, an observation’s low mental health score due to land cover in their living environment might vary. A living environment with too much or too little a certain land type might negatively impact an individual’s mental health status. For example, in terms of urban land features, too high of an urban land percentage means a monotone scene of one’s living environment, but too low of a value indicates a totally rural area without convenient urban services. In other words, based on the SHAP values (Figs. 6, 7, 8, 9, 10, 11, 12, 13), we can judge only whether the current land cover status (Supplementary Materials Fig. S4) positively impacts mental health; however, we never know that the negative status is due to insufficiency or overplus.

**Figure 14:**
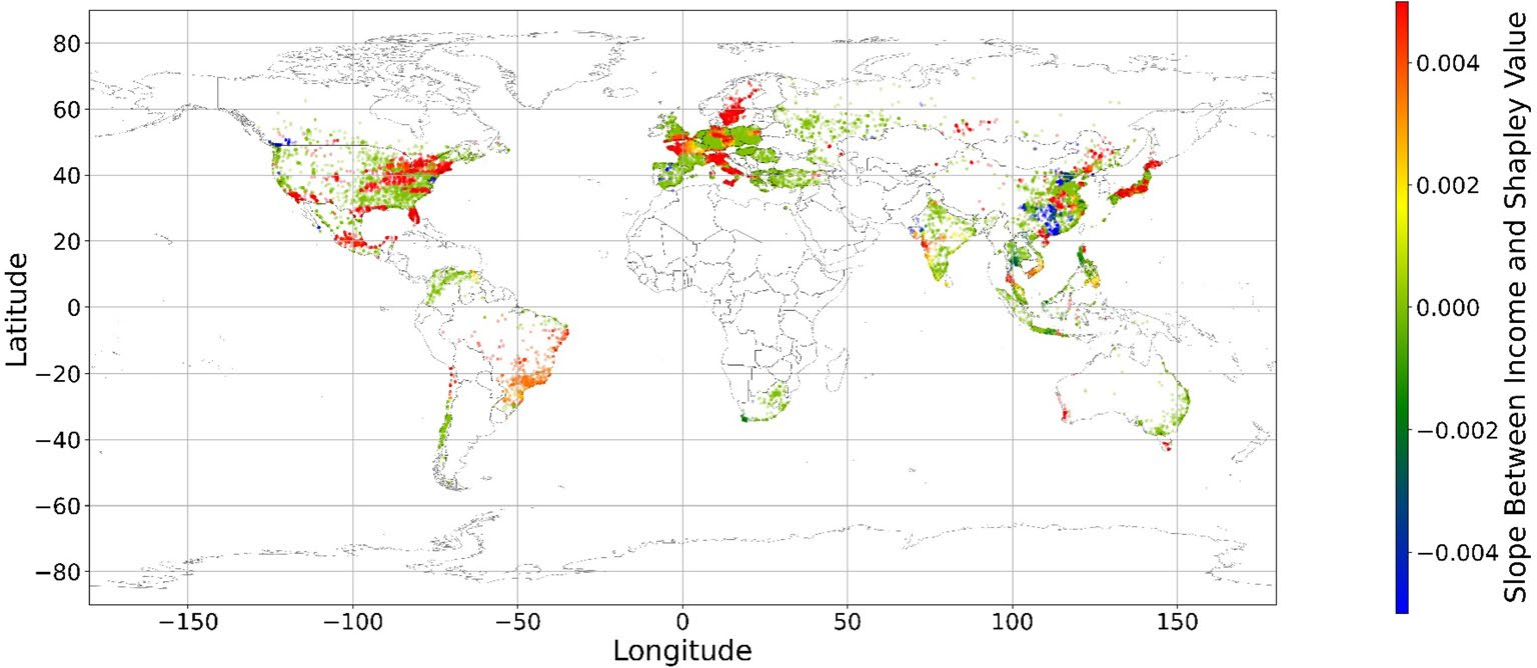
The spatial scatter plot of the local coefficient between income and its SHAP value (Map’s Shapefile is downloaded from https://hub.arcgis.com/datasets/esri::world-countries-generalized/explore; We use Python 3.9.16 to plot https://www.python.org/downloads/release/python-3916/).

The geographical connection between the current land feature value and its SHAP value is desired since the SHAP value cannot inform us that increasing or decreasing specific features would improve one’s mental health. Figure 14 demonstrates nine maps of spatially average local coefficient of income features and land cover features on mental health, based on Eqs. (3)-(5). If a local dataset’s coefficient is nonsignificant, the coefficient would be set to zero. According to Fig. 14, in most zones, a higher RI value is associated with a larger contribution to mental health, while in some metropolitan areas, such as Hong Kong, Beijing, and Washington D.C., a higher RI is negatively related to the SHAP value. The increase in income does not always contribute more to mental health. Previous studies have shown that the relationship between income and human well-being might not be monotonical^55,57^; i.e., there is a turning point in the relationship. In fact, if increased income cannot fulfill more mental needs, then the effects of this increase are limited^58–60^. Furthermore, higher income is usually accompanied by higher levels of responsibility and heavier workloads, which might even worsen the situation^61^. Therefore, the connection between income and its contribution to mental health is negative in these metropolitan areas.

**Figure 15:**
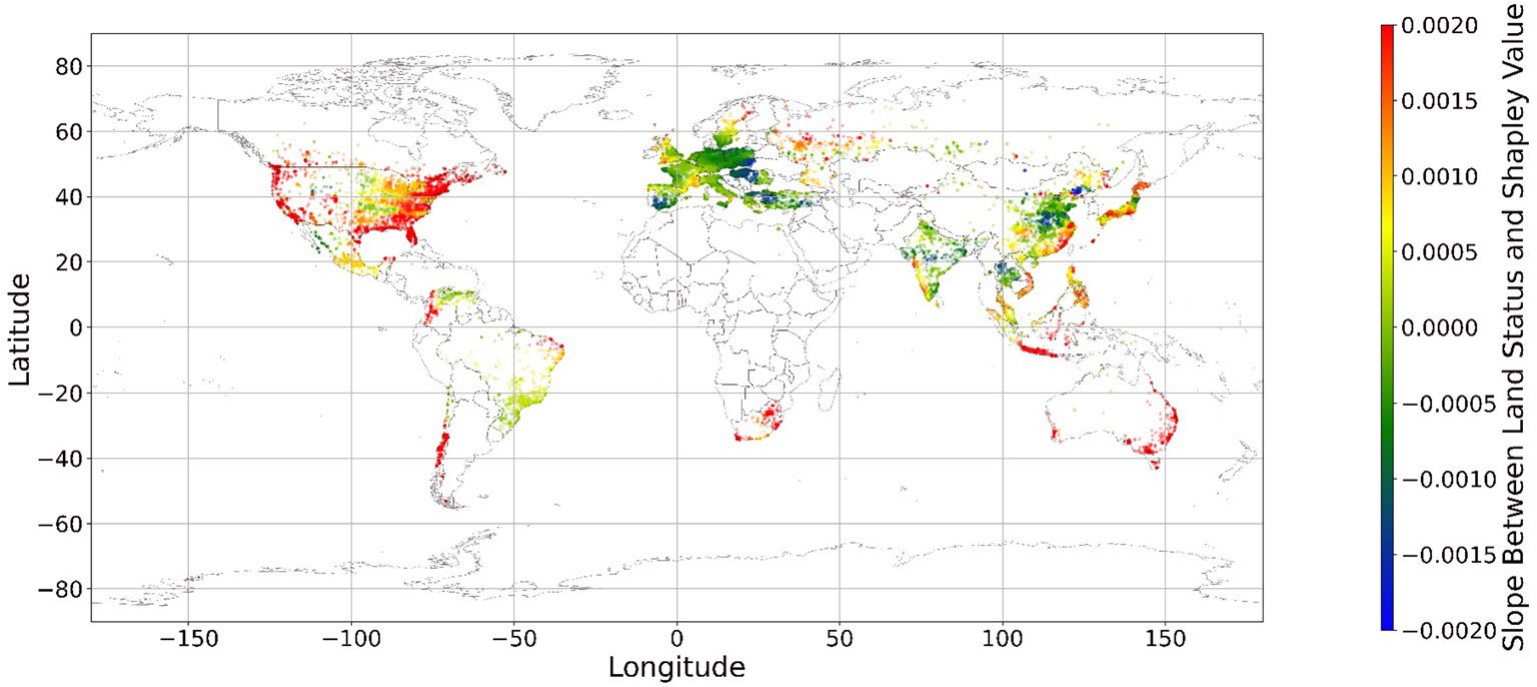
The spatial scatter plot of the local coefficient between cropland and its SHAP value (Map’s Shapefile is downloaded from https://hub.arcgis.com/datasets/esri::world-countries-generalized/explore; We use Python 3.9.16 to plot https://www.python.org/downloads/release/python-3916/).

**Figure 16:**
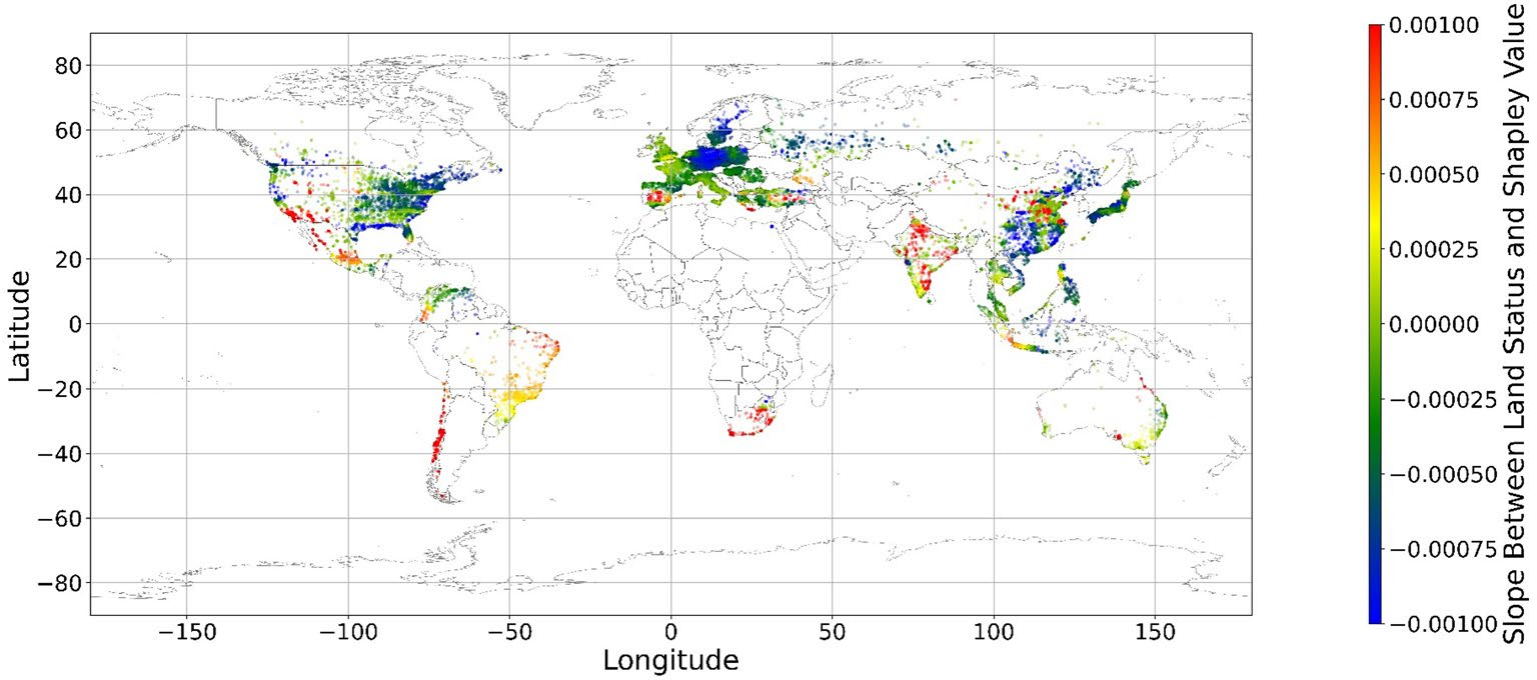
The spatial scatter plot of the local coefficient between forest and its SHAP value.

**Figure 17:**
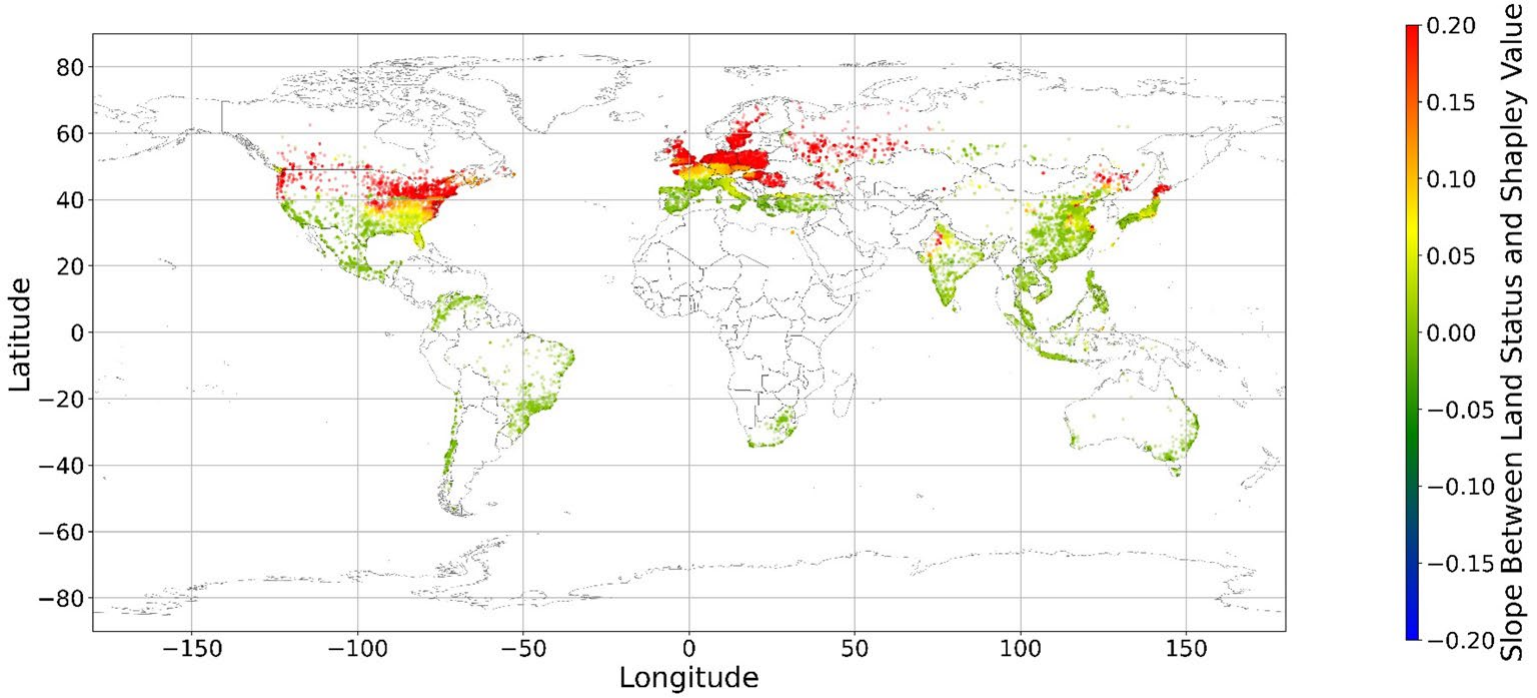
The spatial scatter plot of the local coefficient between shrubland and its SHAP value (Map’s Shapefile is downloaded from https://hub.arcgis.com/datasets/esri::world-countries-generalized/explore; We use Python 3.9.16 to plot https://www.python.org/downloads/release/python-3916/).

**Figure 18:**
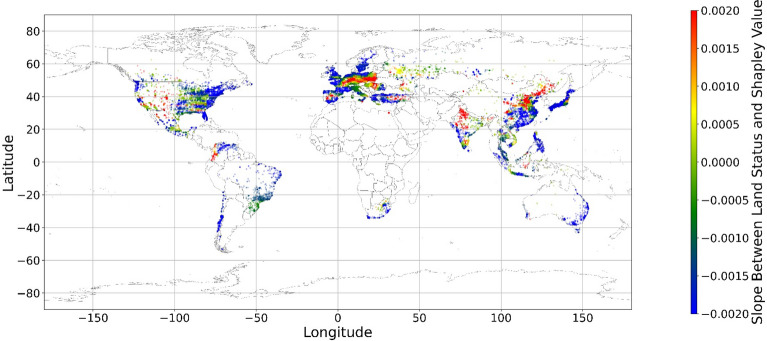
The spatial scatter plot of the local coefficient between water and its SHAP value (map’s Shapefile is downloaded from https://hub.arcgis.com/datasets/esri::world-countries-generalized/explore; We use Python 3.9.16 to plot https://www.python.org/downloads/release/python-3916/).

**Figure 19:**
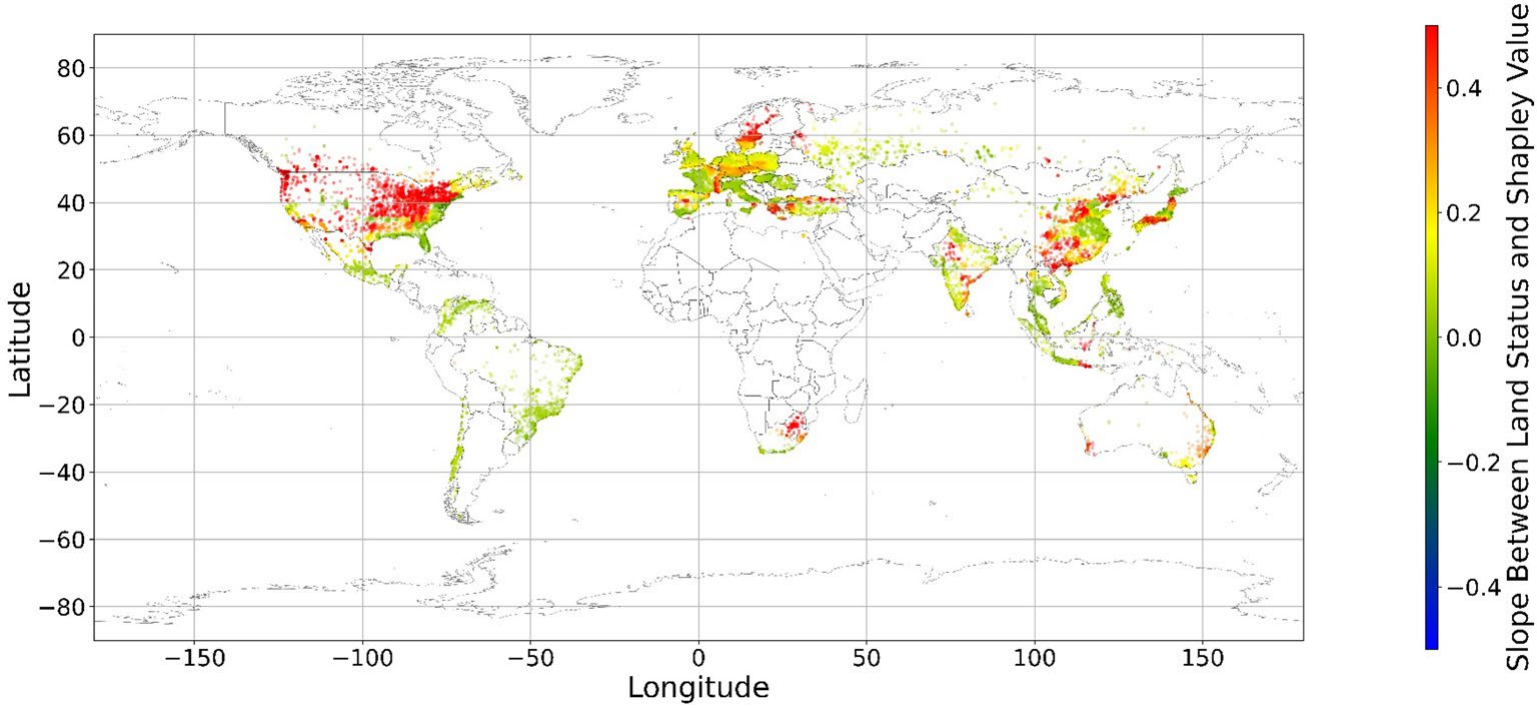
The spatial scatter plot of the local coefficient between wetland and its SHAP value (map’s Shapefile is downloaded from https://hub.arcgis.com/datasets/esri::world-countries-generalized/explore; We use Python 3.9.16 to plot https://www.python.org/downloads/release/python-3916/).

**Figure 20:**
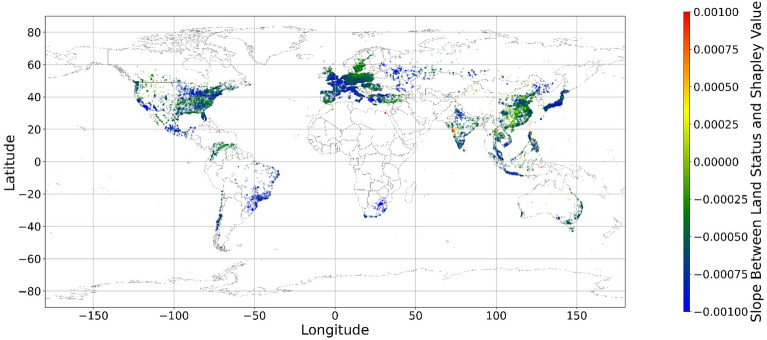
The spatial scatter plot of the local coefficient between urban land and its SHAP value (map’s shapefile is downloaded from https://hub.arcgis.com/datasets/esri::world-countries-generalized/explore; We use Python 3.9.16 to plot https://www.python.org/downloads/release/python-3916/).

**Figure 21:**
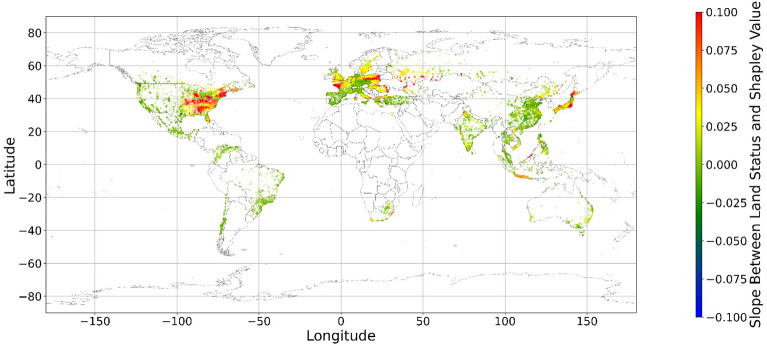
The spatial scatter plot of the local coefficient between bare land and its SHAP value (map’s shapefile is downloaded from https://hub.arcgis.com/datasets/esri::world-countries-generalized/explore; We use Python 3.9.16 to plot https://www.python.org/downloads/release/python-3916/).

**Figure 22:**
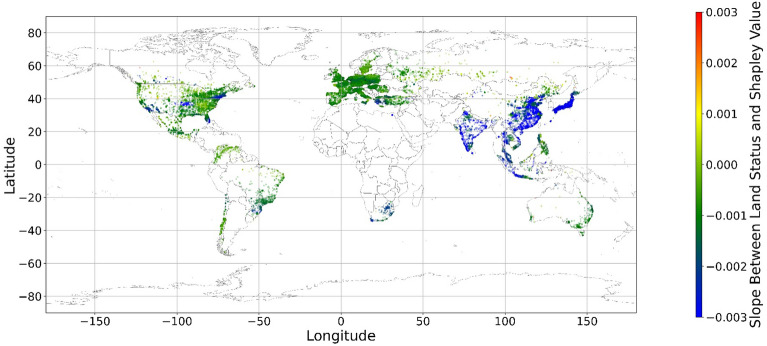
The spatial scatter plot of the local coefficient between grassland and its SHAP value (map’s Shapefile is downloaded from https://hub.arcgis.com/datasets/esri::world-countries-generalized/explore; We use Python 3.9.16 to plot https://www.python.org/downloads/release/python-3916/).

**Figure 23:**
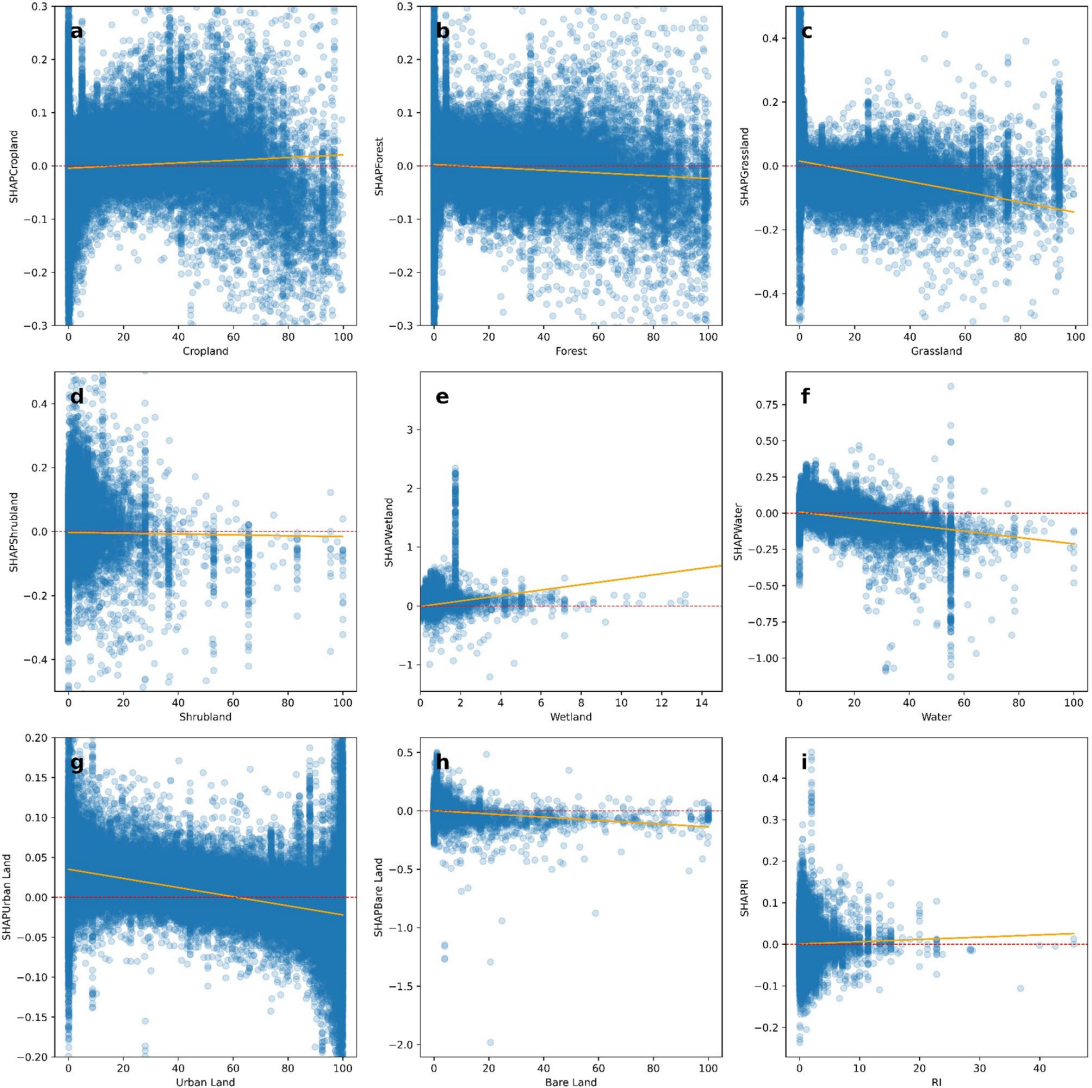
The scatter plot between variables of interest and their SHAPs (Red dashed lines are the ablines where y-axis value equals 0; and yellow lines are linear fitting lines between x-axis value and y-axis value).

Figure 15 shows the local coefficients between cropland status and its SHAP value based on geographically weighted connections. Referring to the current status of cropland (Supplementary Materials Fig. S4.b), in places with too much cropland, an increase in cropland has negative impacts on one’s mental health, whereas in the regions with rare cropland, more cropland could contribute more to one’s mental health. The reason for people’s preferences is scarcity value. According to Figs. 16, 17, 18, 19, 20, and 21, the relationships between forest, grassland, shrubland, water, wetland, urban land, and bare land, and their contributions to mental health are similar to the link found between cropland and its SHAP values. Grassland is an exception, as illustrated by Fig. 22; the relationship between grassland and its contribution to mental health is negative in most places, and the degree of positive connection is relatively low, which is counterintuitive. These are two reasons for this problem. First, this research uses remote sensing data. In the remote sensing process, grassland is more easily misclassified, especially when close to cropland and shrubland^35^. In particular, sporadic grass is more likely to be misclassified; thus, the low accuracy of grassland in urban areas might mislead the model’s results. Second, a large area of grassland is often used for grazing rather than improving mental health in rural areas. Figure 23 illustrates the scatter plots between variables of interest and their SHAP values. Because the distributions shown in Fig. 23 briefly demonstrate the global links, they cannot be directly used to explain the local relationships. For forest, grassland, shrubland, water, urban land, and bare land, when their values are lower, their SHAP values tend to be larger. In other words, when they are scarce, they can obtain the largest values.

**Figure 24:**
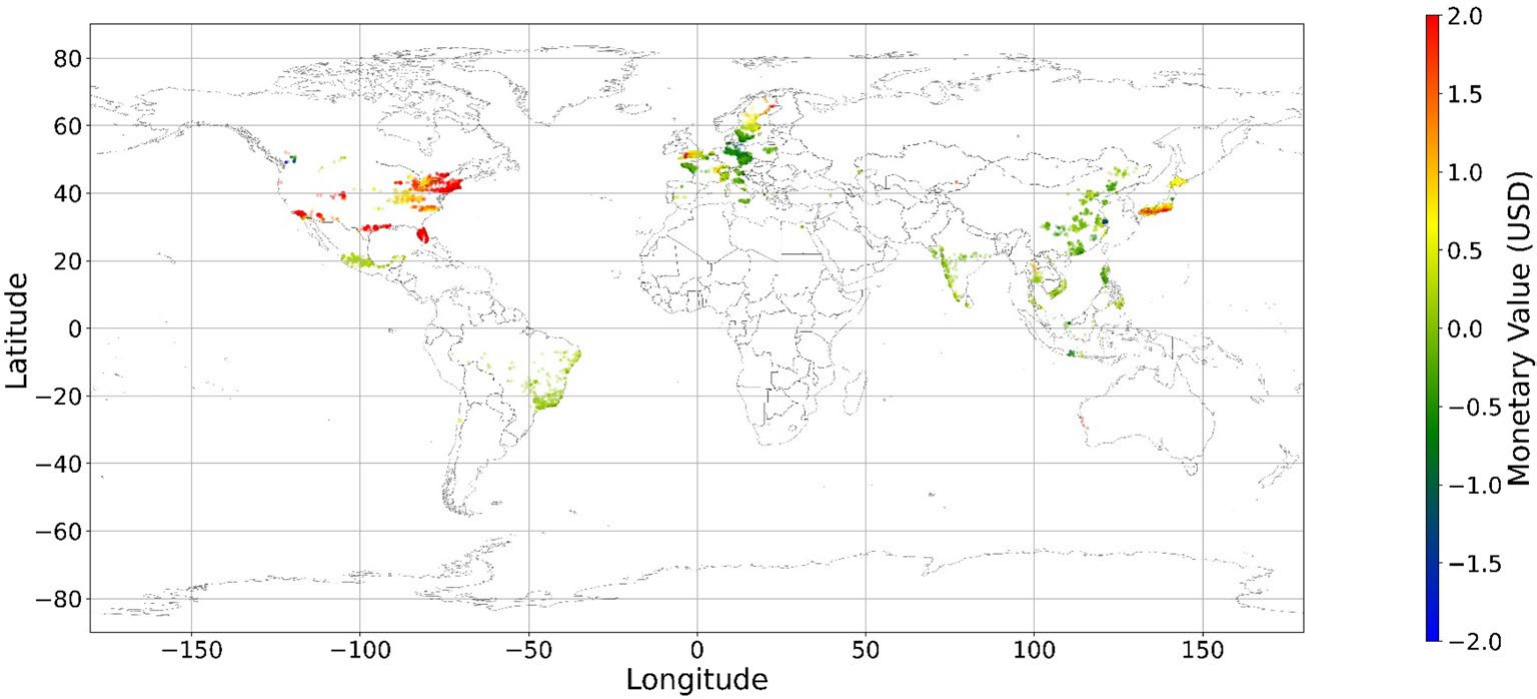
The spatial scatter plot of the monetary value of cropland (Note: Zero has been removed; Map’s Shapefile is downloaded from https://hub.arcgis.com/datasets/esri::world-countries-generalized/explore; We use Python 3.9.16 to plot https://www.python.org/downloads/release/python-3916/).

**Figure 25:**
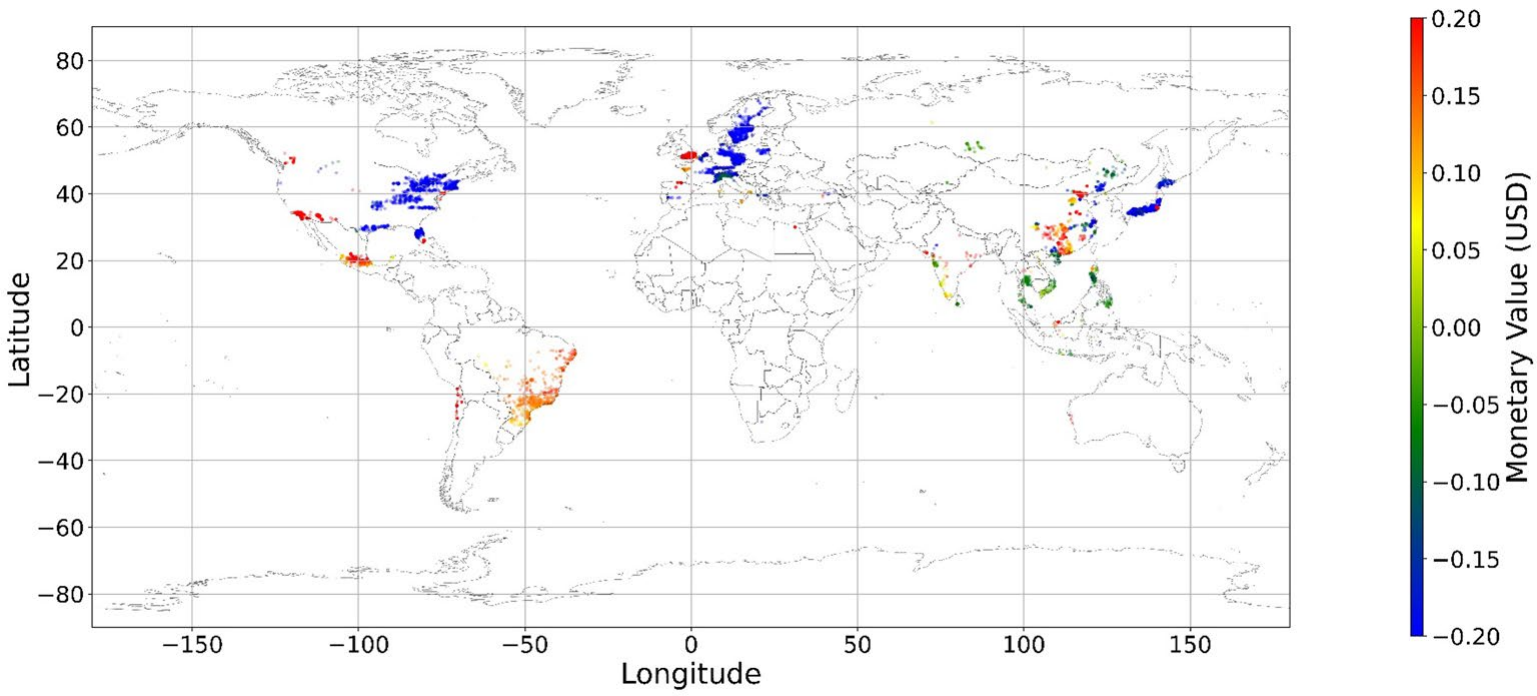
The spatial scatter plot of the monetary value of forest (Note: Zero has been removed; Map’s Shapefile is downloaded from https://hub.arcgis.com/datasets/esri::world-countries-generalized/explore; We use Python 3.9.16 to plot https://www.python.org/downloads/release/python-3916/).

**Figure 26:**
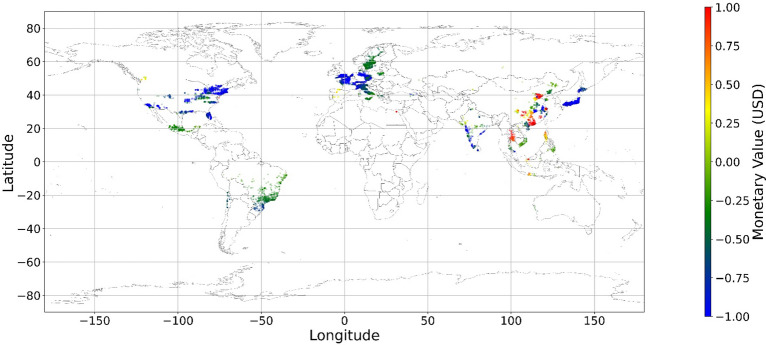
The spatial scatter plot of the monetary value of grassland (Note: Zero has been removed; Map’s Shapefile is downloaded from https://hub.arcgis.com/datasets/esri::world-countries-generalized/explore; We use Python 3.9.16 to plot https://www.python.org/downloads/release/python-3916/).

**Figure 27:**
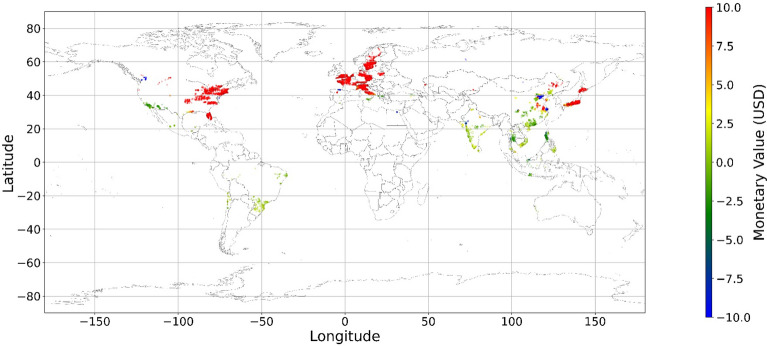
The spatial scatter plot of the monetary value of shrubland (Note: Zero has been removed; Map’s Shapefile is downloaded from https://hub.arcgis.com/datasets/esri::world-countries-generalized/explore; We use Python 3.9.16 to plot https://www.python.org/downloads/release/python-3916/).

**Figure 28:**
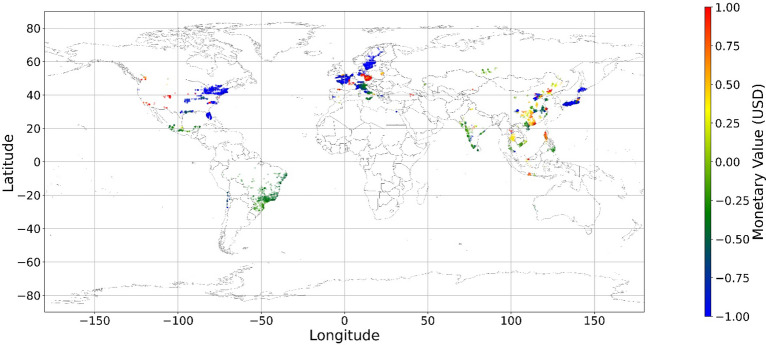
The spatial scatter plot of the monetary value of water (Note: Zero has been removed; map’s shapefile is downloaded from https://hub.arcgis.com/datasets/esri::world-countries-generalized/explore; We use Python 3.9.16 to plot https://www.python.org/downloads/release/python-3916/).

**Figure 29:**
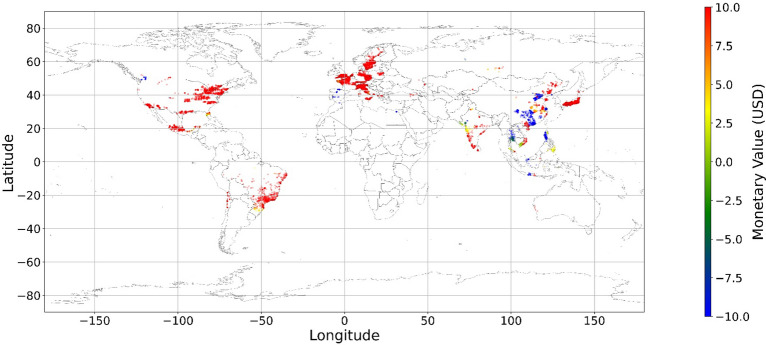
The spatial scatter plot of the monetary value of wetland (Note: Zero has been removed; map’s shapefile is downloaded from https://hub.arcgis.com/datasets/esri::world-countries-generalized/explore; We use Python 3.9.16 to plot https://www.python.org/downloads/release/python-3916/).

**Figure 30:**
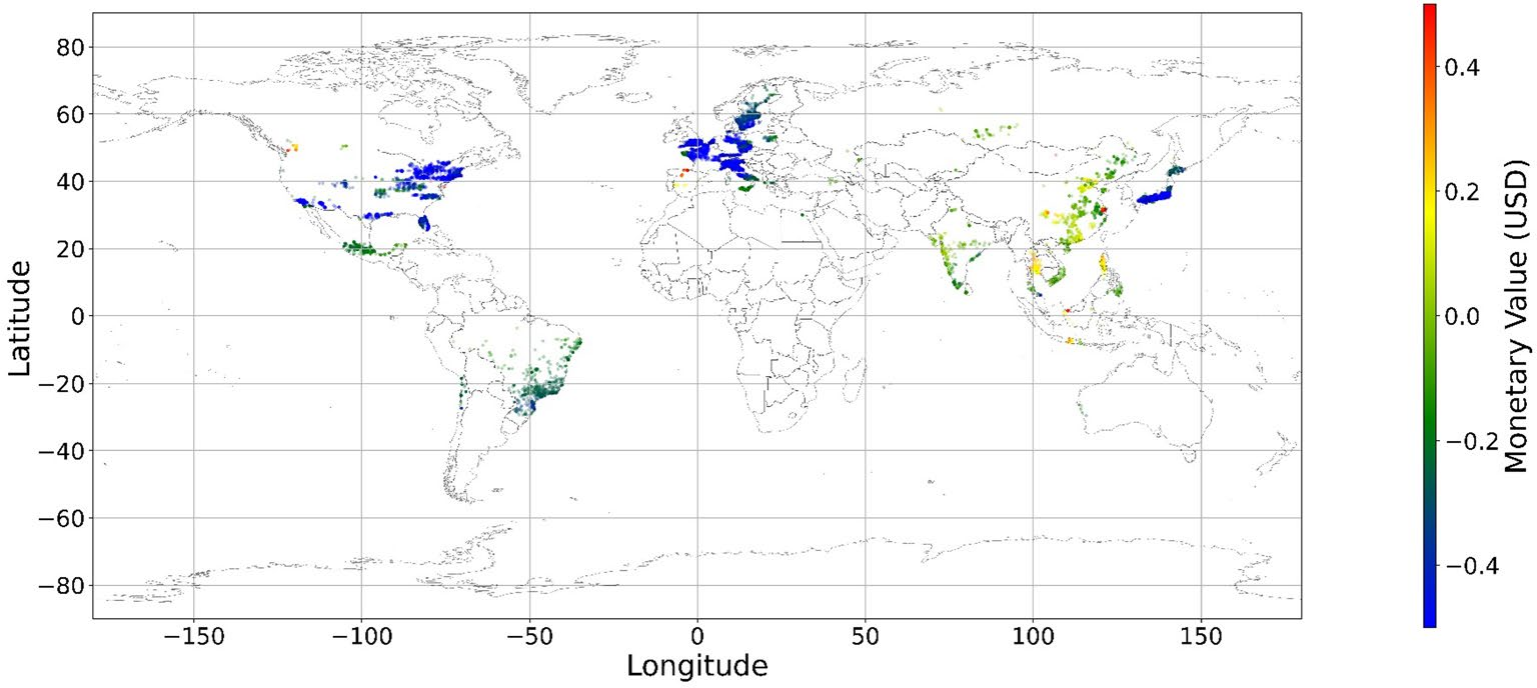
The spatial scatter plot of the monetary value of urban land (Note: Zero has been removed; map’s shapefile is downloaded from https://hub.arcgis.com/datasets/esri::world-countries-generalized/explore; We use Python 3.9.16 to plot https://www.python.org/downloads/release/python-3916/).

**Figure 31:**
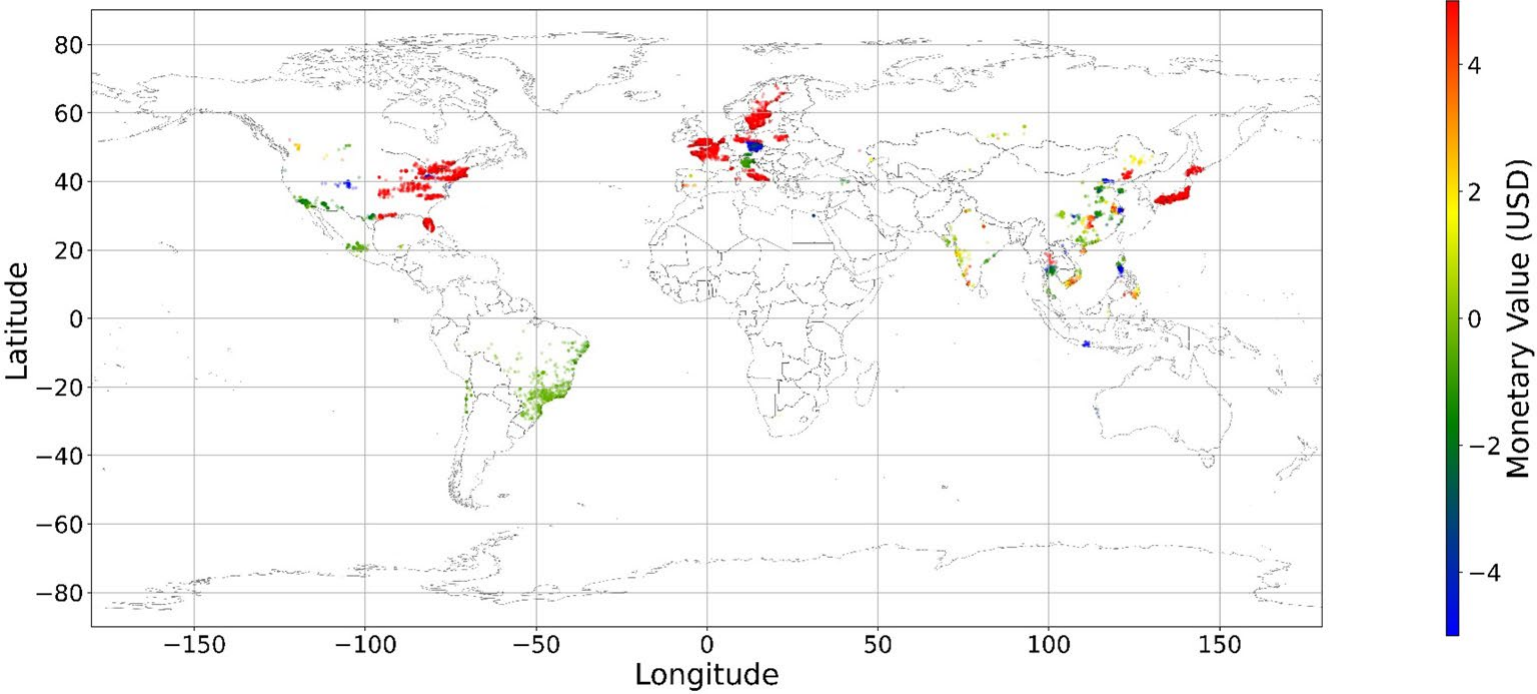
The spatial scatter plot of the monetary value of bare land (Note: Zero has been removed; map’s shapefile is downloaded from https://hub.arcgis.com/datasets/esri::world-countries-generalized/explore; We use Python 3.9.16 to plot https://www.python.org/downloads/release/python-3916/).

Figure 24, 25, 26, 27, 28, 29, 30 and 31 illustrates the spatially average monetary values of eight land types, according to Eq. (6). As shown in Fig. 24, the monetary values of cropland are higher in metropolitan areas such as New York, London, Paris, and Tokyo, among others. Forest and water monetary values (Figs. 25 and 28) are also higher in large cities. Grassland’s and urban land’s monetary values (Figs. 26 and 30) are positive when the contribution of an increase in income is negative. In most places, their monetary values are favorable due to the scarcity values of shrubland, wetland, and bare land (Figs. 27, 29, and 11). Importantly, shrubland, wetland, and bare land are very rare in most living environments (as shown in Figs. S4e,f,h). A slight increase in wetland, shrubland, or bare land is difficult. This is the reason for their extraordinary monetary value, which is consistent with previous studies^8^.

## Discussion

Our main findings are that mental health and land cover relationships are geographically local and spatially varied. Increases in each land type positively impact mental health when the percentages of these land types are low. Accordingly, it could be implied that people who prefer to live in environments with high diversity and extremely monolithic landscapes might have poor mental health. Furthermore, this is the first study that uses SHAP and random forest to grasp the relationship between land cover and mental health. To make the results understandable, we employ geographically local technology to connect the current land cover status to its SHAP values. This study provides one more way in which to explain the machine learning model. Based on the links between the SHAP value and current status, the monetary values of land cover are estimated, although the numbers of significant monetary values of land cover are limited. Our results show that a slight increase in shrubland, wetland, and bare land in most regions could improve people’s mental health. Cropland, forest, and water are mainly desired in metropolitan areas and places with too little cropland, forest, and water. Moreover, the model’s accuracy is relatively high, indicating the reliability of the results. The accuracy, RMSE, MSE, and MAE values are 67.59%, 3.59, 12.87, and 2.71, respectively, exceeding those of most previous studies.

Previous studies have focused more on the impacts of green space on human well-being or mental health in cities^6,29,30,32,38^. The coverage percentage of green space positively affects mental health^6,32,38^. In our study, almost all natural land types are positively related to mental health when their percentages are low, as illustrated in Fig. 23. A relatively higher proportion of natural land can promote direct and indirect interactions between humans and nature^28,29,62–64^. Nature-based recreation is a typical interaction, which could improve mental health through restoration^65,66^. Furthermore, a relatively higher natural environment ratio could increase physical actions in nature^4,9^. This is the key reason supporting the connections we find in this study. A one-unit increase in the wetland is associated with the largest potential increase in mental health, as shown in Figs. 14, 15, 16, 22, 17, 18, 19, 20 and 21, compared with other land types. Wetland is the most preferred, as it provides the most ecosystem service^7,8^, and it is scarce in the living environment. Bare land’s average SHAP values and monetary values are high. According to the figure in the data provider’s article^35^, large areas of bare land are generally desert, although they might be used as sports play yards when located within a city. Shrubland’s situation is similar to that of wetland and bare land, and their scarcity positively impacts mental health. Forest and cropland effects vary. In metropolitan areas, increased cropland and forest percentages improve mental health. It is relatively difficult for people to enter large areas of forest to have various natural experiences; these areas are also associated with the possibility of crime^10,67^. A high percentage of urban land is negatively associated with mental health. Living in cities naturally is necessary^68–70^. However, the adverse effects of large amounts of non urban land types on mental health indicate that people living in rural areas are likely to have mental disorders and need more assistants. Therefore, in regard to land use, the percentage of urban land should be carefully treated and balanced.

The biggest contribution of this study is providing a new way in which to employ a machine learning method, namely, random forest, to analyze the data with geographic information. The random forest method is good at grasping nonparametric relationships, thereby, improving the model’s accuracy and making the explanation more reliable. Directly adding geographical locations to the analysis in the random forest model makes the analysis take geographical context into account because the model deems that the neighbor observations are similar. However, this does not work in traditional regression methods, such as OLS, spatial autoregressive regression, spatial lag X regression, and spatial error regression^71^, as the coefficients of longitude and latitude are hard to explain. Importantly, we are not denying the importance of OLS. In contrast, our method serves as an improvement on the traditional model. Currently, the widely used approaches used to explain random forest results are partial dependence plots^72^, accumulated local effects^73^, and Shapley values^40,41^. Among these three methods, the Shapley value approach has the most solid theoretical foundation^53^. However, Shapley value explanations are entirely local. In other words, one observation’s explanation cannot be directly used on other observations. For this reason, building reasonable connections between Shapley values and feature values is critical in related studies. Links created by geographically weighted regression methods are spatially continuous. The relationship coefficients of each location do not suddenly change and are more similar if they are closer together, which is more consistent with the real world. This connection method makes the relationship between the feature values and their contribution more understandable.

There are several limitations and issues worthy of note. First, the land cover variables represent the percentages of eight land types present in the buffers within a 5-km radius surrounding the living locations of respondents. There is an assumption that the quality of land cover does not influence the effects of those land types on mental health. For example, there may be no difference between a well-designed urban park and grassland in a pasture. Furthermore, the impacts of the distance to a certain land type are ignored. Second, this study uses only global cross-sectional data; thus, it cannot detect differences within individuals when land cover changes. Thus, global research using panel data to probe the effects within individuals is still desired. Third, the number of respondents in each country is not the same or even proportional to the country’s population. Countries with more respondents have more substantial impacts on the results. Thus, the results might be prejudiced, although this database is one of the largest databases available in this field. Fourth, due to the limits of surveying fees, we cannot investigate the temporal variation in mental health, including seasonal and annual variation. In future studies, long-term panel data should be used to investigate the impacts of land cover within individuals. Moreover, the model’s cross-validation accuracy is not ideal, which might make the SHAP values inaccurate. Further improvement of the model is needed. Effective explanatory methods and tools should be developed to make the machine learning results understandable.

## Conclusion

The relationships between land cover in living environments and mental health are more complex than linear assumptions. An unsuitable increase in a specific land type might not improve residents’ mental health. Among the eight land types, shrubland, wetland, and bare land have the highest effects on mental health due to their scarcity in living environments. The impacts of cropland, forest, and water are high, mainly in metropolitan areas. In contrast, the impacts of urban land and grassland are mainly negative. Our study illustrates the heterogeneity of the effects of eight land types on mental health to provide more information for governments and the public. Furthermore, this research offers one example of analyzing data with geographical information by random forest and explaining the results geographically.

### Supplementary Information

Supplementary Information.

## Data Availability

The fully reproducible codes are publicly available at 10.5281/zenodo.10450766. Data are available from the corresponding author on reasonable request.
