# Supplementary material for: Mental health and natural land cover: a global analysis based on random forest with geographical consideration
Source: Sci Rep. 2024 Feb 5;14:2894. doi: 10.1038/s41598-024-53279-7 (PMC10844245; doi:10.1038/s41598-024-53279-7)
Supplement: Supplementary file 1 — Supplementary Information. [file 41598_2024_53279_MOESM1_ESM.docx]

Supplementary Materials to:

Mental Health and Natural Land Cover: A Global Analysis Based on Random Forest with Geographical Consideration

# Authors

Chao Li^1^, Shunsuke Managi*^1^

# Affiliations

1 Urban Institute, Kyushu University, Japan

* Correspondent to: Shunsuke Managi, managi@doc.kyushu-u.ac.jp, Kyushu University 744 Motooka, Nishi-ku, Fukuoka 819-0395 Japan

# Data and Methods

## Individual Income Data

An essential factor affecting mental health is individual income. We include this variable as a covariate in our analysis. In this study, we use the ratio between individual income and GDP per capita in the respondent’s country (RI) as the income variable because the income in the survey is based on local currency. The main reason is that the same amounts of money might have different effects in different countries. For example, the purchasing power of 100 USD in the U.S. and Sri Lanka are not the same for the local people in each location. The RI is calculated as follows:

| ${RI}_{i}= {Inc}_{i}-{GDPPC}_{i}$ | (1) |
| --- | --- |

where ${RI}_{i}$ is the RI of respondent $i$, ${Inc}_{i}$ is the individual income of respondent $i$, and ${GDPPC}_{i}$ is the GDP per capita of respondent $i$’s country in the surveyed year. Importantly, the units of ${Inc}_{i}$ and ${GDPPC}_{i}$ are the current price of international USD. To unify the individual income data, we also convert the local currencies into the current price of international USD. We employ the official annual average exchange rate of the year during the conversion process when the survey was conducted in that country. Moreover, the survey questionnaires ask the respondents to select their gross household income range rather than to report their exact gross household income. Thus, we take the midpoint of the range chosen by the respondent as the gross household income. For instance, in the U.S., if respondents report that their gross household income ranges from 50,000 to 60,000 USD per year, then their household income is considered 55,000 USD per year in the analysis. According to previous studies 46,47, the calculation of annual gross individual income is as follows:

| ${Inc}_{i}=\frac{{GHI}_{i}}{{({Adu}_{i}+0.7{Chi}_{i})}^{0.5}}$ | (2) |
| --- | --- |

where ${Inc}_{i}$ is the annual gross individual income of respondent $i$, ${GHI}_{i}$ represents the gross annual household income of respondent $i$, ${Adu}_{i}$ represents the number of adults in respondent $i$’s household, and ${Chi}_{i}$ represents the number of children in respondent $i$’s household. Limited by the household size and the maximum value of the selection interval, individual income rarely exceeds four times the GDP per capita in the respondent’s country. (**Supplementary Material** **Figure s1** demonstrates the statistical distribution of the RI in the respondent’s country.)

## Decision Tree and Random Forest

A single decision tree is the fundamental element of the random forest method. There are two types of trees, namely, decision trees for either classification or regression ^50,51^. **Figure S2** shows a simple example of a three-layer regression decision tree. To complete the prediction in the example tree, the algorithm passes three internodes and makes three judgments at most. As seen in the example illustrated in **Figure S2**, we assume that only three features, namely, the unemployed dummy variable, self-reported health, and the RI, affect the output variable, i.e., mental health. The rules of each judgment and feature range split are a critical part of machine learning training. A large amount of data is put into the algorithm to train the decision tree to decide the rules of each judgment and feature range split. We employ a greedy approach to train regression decision trees ^52^. This approach chooses the features and splits their ranges to minimize the residual sum of squares (RSS) as follows:

| $RSS= \sum_{l\in leaves} \sum_{i\in C_{l}} {(y_{i} - \bar{y}_{C_{l}})}^{2}$ | (2) |
| --- | --- |

where $l$ is a leaf, $C_{l}$ is the case in leaf $l$, $y_{i}$ is the observed value and $\bar{y}_{C_{l}}$ is the average observed value in leaf $l$. Unless the RSS is smaller than the defined threshold or the number of remaining cases in the end leaf is less than the defined threshold, the number of internodes of trees will continue to increase ^52^. These decision tree algorithms are nonlinear and closer to real-world situations ^53^. In most cases, a single regression decision tree is insufficient to fit the output variables and usually causes an overfitting analysis. Models that assemble a bundle of decision trees could solve the overfitting issues smoothly, these models are named boosting models.

# Table:

| **Table S1: Country-level Mnetal Health Status Counts** | | | | | | | | | | |
| --- | --- | --- | --- | --- | --- | --- | --- | --- | --- | --- |
| GHQ12 | Australia | Brazil | Canada | Chile | China | Colombia | Czech | Egypt | France | Germany |
| 0 | 12 | 2 | 2 | 1 | 23 | 0 | 0 | 10 | 5 | 4 |
| 1 | 7 | 1 | 1 | 1 | 7 | 0 | 2 | 2 | 3 | 4 |
| 2 | 4 | 3 | 4 | 0 | 9 | 1 | 2 | 4 | 4 | 4 |
| 3 | 7 | 7 | 2 | 0 | 10 | 0 | 1 | 3 | 6 | 8 |
| 4 | 7 | 4 | 3 | 0 | 13 | 1 | 3 | 2 | 4 | 3 |
| 5 | 13 | 6 | 3 | 3 | 12 | 0 | 1 | 1 | 5 | 11 |
| 6 | 9 | 8 | 3 | 2 | 26 | 0 | 1 | 0 | 7 | 14 |
| 7 | 10 | 10 | 6 | 6 | 28 | 2 | 7 | 4 | 8 | 16 |
| 8 | 12 | 14 | 8 | 5 | 29 | 4 | 6 | 1 | 5 | 19 |
| 9 | 17 | 10 | 11 | 9 | 38 | 2 | 2 | 1 | 15 | 27 |
| 10 | 12 | 15 | 9 | 8 | 38 | 3 | 6 | 3 | 10 | 28 |
| 11 | 15 | 21 | 6 | 9 | 50 | 3 | 10 | 4 | 10 | 35 |
| 12 | 27 | 27 | 6 | 12 | 129 | 11 | 12 | 18 | 16 | 37 |
| 13 | 19 | 27 | 7 | 13 | 124 | 9 | 17 | 5 | 22 | 43 |
| 14 | 17 | 21 | 21 | 13 | 124 | 7 | 15 | 6 | 34 | 45 |
| 15 | 36 | 32 | 19 | 15 | 180 | 13 | 26 | 2 | 32 | 53 |
| 16 | 42 | 37 | 31 | 26 | 222 | 15 | 30 | 4 | 33 | 49 |
| 17 | 49 | 60 | 29 | 18 | 277 | 16 | 20 | 3 | 57 | 73 |
| 18 | 32 | 54 | 44 | 37 | 360 | 22 | 29 | 8 | 56 | 76 |
| 19 | 57 | 61 | 38 | 29 | 404 | 18 | 41 | 8 | 68 | 107 |
| 20 | 58 | 74 | 44 | 31 | 526 | 34 | 41 | 9 | 74 | 115 |
| 21 | 65 | 87 | 52 | 36 | 659 | 25 | 38 | 4 | 83 | 110 |
| 22 | 104 | 67 | 59 | 37 | 690 | 41 | 55 | 8 | 125 | 120 |
| 23 | 89 | 103 | 72 | 61 | 960 | 47 | 55 | 13 | 161 | 145 |
| 24 | 192 | 127 | 140 | 78 | 3066 | 52 | 110 | 79 | 324 | 283 |
| 25 | 130 | 97 | 100 | 79 | 1129 | 61 | 90 | 21 | 165 | 221 |
| 26 | 118 | 134 | 87 | 99 | 1180 | 80 | 107 | 42 | 147 | 187 |
| 27 | 118 | 148 | 101 | 89 | 1249 | 98 | 100 | 29 | 121 | 212 |
| 28 | 118 | 169 | 69 | 69 | 1246 | 92 | 73 | 23 | 111 | 187 |
| 29 | 97 | 145 | 80 | 75 | 1232 | 81 | 57 | 24 | 89 | 195 |
| 30 | 127 | 133 | 79 | 61 | 1477 | 75 | 43 | 97 | 68 | 189 |
| 31 | 24 | 98 | 32 | 34 | 1011 | 48 | 12 | 13 | 21 | 73 |
| 32 | 18 | 88 | 12 | 32 | 877 | 42 | 11 | 21 | 11 | 28 |
| 33 | 14 | 59 | 11 | 22 | 604 | 33 | 0 | 17 | 2 | 12 |
| 34 | 10 | 53 | 7 | 16 | 347 | 26 | 2 | 20 | 5 | 11 |
| 35 | 9 | 40 | 6 | 10 | 251 | 20 | 0 | 19 | 3 | 3 |
| 36 | 9 | 39 | 9 | 22 | 961 | 31 | 1 | 38 | 17 | 7 |

| **Table S1: Country-level Mnetal Health Status Counts (Continuous)** | | | | | | | | | | |
| --- | --- | --- | --- | --- | --- | --- | --- | --- | --- | --- |
| GHQ12 | Greece | Hungary | India | Indonesia | Italy | Japan | Kazakhstan | Malaysia | Mexico | Mongolia |
| 0 | 1 | 1 | 6 | 1 | 1 | 60 | 0 | 3 | 0 | 0 |
| 1 | 1 | 0 | 3 | 2 | 0 | 27 | 0 | 4 | 0 | 0 |
| 2 | 3 | 5 | 1 | 2 | 0 | 27 | 0 | 0 | 0 | 0 |
| 3 | 3 | 0 | 7 | 1 | 1 | 38 | 1 | 3 | 1 | 1 |
| 4 | 8 | 6 | 8 | 0 | 0 | 39 | 0 | 1 | 2 | 0 |
| 5 | 4 | 8 | 4 | 3 | 5 | 37 | 0 | 4 | 3 | 0 |
| 6 | 8 | 10 | 13 | 0 | 4 | 51 | 3 | 4 | 4 | 0 |
| 7 | 8 | 15 | 10 | 5 | 3 | 39 | 0 | 3 | 2 | 0 |
| 8 | 15 | 11 | 13 | 4 | 3 | 48 | 2 | 5 | 2 | 0 |
| 9 | 19 | 9 | 25 | 7 | 8 | 79 | 1 | 8 | 3 | 1 |
| 10 | 17 | 7 | 18 | 5 | 9 | 55 | 1 | 14 | 6 | 2 |
| 11 | 20 | 18 | 33 | 8 | 18 | 83 | 2 | 12 | 9 | 0 |
| 12 | 26 | 17 | 41 | 15 | 28 | 147 | 3 | 13 | 8 | 1 |
| 13 | 30 | 22 | 65 | 19 | 19 | 152 | 3 | 10 | 15 | 2 |
| 14 | 36 | 29 | 59 | 16 | 36 | 163 | 3 | 13 | 13 | 1 |
| 15 | 34 | 27 | 77 | 19 | 38 | 182 | 5 | 12 | 25 | 0 |
| 16 | 41 | 28 | 93 | 30 | 31 | 213 | 5 | 15 | 24 | 7 |
| 17 | 50 | 28 | 127 | 43 | 42 | 233 | 7 | 27 | 25 | 9 |
| 18 | 37 | 40 | 188 | 48 | 69 | 251 | 3 | 39 | 35 | 6 |
| 19 | 45 | 37 | 141 | 39 | 70 | 269 | 13 | 33 | 37 | 7 |
| 20 | 70 | 61 | 177 | 70 | 93 | 347 | 14 | 51 | 42 | 23 |
| 21 | 54 | 45 | 235 | 62 | 82 | 397 | 14 | 51 | 52 | 20 |
| 22 | 75 | 50 | 256 | 91 | 132 | 419 | 19 | 57 | 74 | 22 |
| 23 | 73 | 88 | 282 | 90 | 157 | 529 | 18 | 59 | 67 | 20 |
| 24 | 123 | 118 | 478 | 179 | 242 | 1045 | 44 | 124 | 104 | 36 |
| 25 | 85 | 89 | 329 | 143 | 184 | 670 | 36 | 62 | 95 | 36 |
| 26 | 58 | 103 | 328 | 138 | 166 | 525 | 64 | 62 | 129 | 34 |
| 27 | 69 | 71 | 339 | 127 | 146 | 535 | 129 | 64 | 130 | 38 |
| 28 | 70 | 77 | 381 | 161 | 133 | 596 | 65 | 57 | 134 | 47 |
| 29 | 45 | 40 | 449 | 193 | 64 | 642 | 81 | 45 | 120 | 33 |
| 30 | 34 | 36 | 558 | 207 | 38 | 715 | 113 | 50 | 108 | 43 |
| 31 | 27 | 23 | 304 | 112 | 12 | 174 | 46 | 37 | 73 | 28 |
| 32 | 10 | 11 | 203 | 93 | 8 | 86 | 30 | 24 | 60 | 20 |
| 33 | 6 | 4 | 218 | 66 | 4 | 68 | 27 | 11 | 40 | 10 |
| 34 | 1 | 3 | 169 | 55 | 2 | 28 | 19 | 6 | 28 | 6 |
| 35 | 3 | 3 | 104 | 38 | 1 | 29 | 13 | 10 | 24 | 2 |
| 36 | 0 | 4 | 399 | 114 | 3 | 38 | 15 | 15 | 24 | 1 |

| **Table S1: Country-level Mnetal Health Status Counts (Continuous)** | | | | | | | | | | |
| --- | --- | --- | --- | --- | --- | --- | --- | --- | --- | --- |
| GHQ12 | Myanmar | Netherlands | Philippines | Poland | Romania | Russia | Singapore | South Africa | Spain | Sri Lanka |
| 0 | 0 | 1 | 1 | 3 | 0 | 0 | 2 | 0 | 0 | 2 |
| 1 | 0 | 2 | 1 | 2 | 0 | 2 | 5 | 2 | 3 | 0 |
| 2 | 0 | 1 | 0 | 5 | 0 | 4 | 2 | 3 | 6 | 0 |
| 3 | 0 | 5 | 1 | 7 | 1 | 5 | 4 | 6 | 3 | 0 |
| 4 | 0 | 3 | 4 | 7 | 1 | 6 | 1 | 4 | 3 | 0 |
| 5 | 0 | 3 | 1 | 8 | 1 | 5 | 4 | 6 | 4 | 0 |
| 6 | 0 | 4 | 4 | 8 | 0 | 6 | 1 | 12 | 5 | 1 |
| 7 | 0 | 4 | 2 | 8 | 0 | 6 | 2 | 13 | 6 | 0 |
| 8 | 0 | 4 | 3 | 17 | 2 | 15 | 1 | 7 | 18 | 1 |
| 9 | 0 | 7 | 8 | 17 | 0 | 20 | 1 | 14 | 8 | 0 |
| 10 | 0 | 8 | 5 | 14 | 1 | 14 | 3 | 5 | 11 | 1 |
| 11 | 0 | 10 | 6 | 21 | 1 | 17 | 3 | 15 | 11 | 0 |
| 12 | 0 | 4 | 10 | 17 | 5 | 18 | 5 | 23 | 18 | 2 |
| 13 | 1 | 13 | 8 | 19 | 5 | 31 | 9 | 24 | 25 | 1 |
| 14 | 1 | 12 | 9 | 29 | 4 | 35 | 10 | 25 | 26 | 0 |
| 15 | 1 | 18 | 21 | 44 | 6 | 42 | 11 | 25 | 24 | 3 |
| 16 | 4 | 25 | 28 | 43 | 8 | 45 | 16 | 22 | 44 | 4 |
| 17 | 4 | 24 | 32 | 54 | 7 | 61 | 11 | 38 | 35 | 1 |
| 18 | 3 | 33 | 31 | 67 | 15 | 60 | 17 | 24 | 45 | 5 |
| 19 | 7 | 32 | 51 | 81 | 9 | 67 | 15 | 29 | 50 | 8 |
| 20 | 9 | 35 | 45 | 70 | 8 | 85 | 20 | 39 | 50 | 6 |
| 21 | 3 | 44 | 67 | 85 | 9 | 100 | 20 | 46 | 72 | 9 |
| 22 | 2 | 44 | 59 | 90 | 15 | 123 | 23 | 58 | 78 | 2 |
| 23 | 8 | 58 | 74 | 95 | 20 | 113 | 31 | 51 | 102 | 12 |
| 24 | 135 | 95 | 126 | 179 | 20 | 209 | 64 | 81 | 209 | 7 |
| 25 | 49 | 66 | 124 | 140 | 28 | 170 | 38 | 55 | 178 | 9 |
| 26 | 81 | 79 | 118 | 136 | 22 | 174 | 25 | 70 | 147 | 9 |
| 27 | 106 | 87 | 93 | 115 | 13 | 201 | 35 | 61 | 168 | 12 |
| 28 | 94 | 79 | 102 | 108 | 16 | 156 | 22 | 48 | 161 | 31 |
| 29 | 116 | 98 | 102 | 140 | 14 | 115 | 17 | 44 | 144 | 31 |
| 30 | 227 | 129 | 100 | 109 | 13 | 69 | 35 | 42 | 108 | 198 |
| 31 | 72 | 40 | 67 | 37 | 4 | 28 | 14 | 24 | 33 | 60 |
| 32 | 54 | 19 | 63 | 21 | 5 | 13 | 12 | 19 | 27 | 22 |
| 33 | 24 | 5 | 52 | 7 | 0 | 13 | 5 | 11 | 12 | 12 |
| 34 | 23 | 6 | 26 | 1 | 1 | 4 | 10 | 12 | 5 | 13 |
| 35 | 10 | 2 | 31 | 2 | 1 | 5 | 2 | 10 | 9 | 5 |
| 36 | 13 | 8 | 32 | 4 | 0 | 4 | 6 | 10 | 12 | 3 |

| **Table S1: Country-level Mnetal Health Status Counts (Continuous)** | | | | | | | |
| --- | --- | --- | --- | --- | --- | --- | --- |
| GHQ12 | Sweden | Thailand | Turkey | United Kingdom | United States | Venezuela | Vietnam |
| 0 | 0 | 2 | 3 | 15 | 37 | 1 | 1 |
| 1 | 1 | 0 | 3 | 10 | 14 | 0 | 0 |
| 2 | 0 | 3 | 6 | 8 | 33 | 2 | 1 |
| 3 | 0 | 0 | 13 | 14 | 59 | 0 | 1 |
| 4 | 4 | 0 | 7 | 17 | 35 | 0 | 1 |
| 5 | 2 | 1 | 11 | 9 | 38 | 1 | 1 |
| 6 | 4 | 6 | 14 | 12 | 52 | 0 | 5 |
| 7 | 3 | 1 | 15 | 20 | 67 | 1 | 3 |
| 8 | 8 | 0 | 13 | 20 | 66 | 1 | 5 |
| 9 | 11 | 2 | 21 | 24 | 97 | 3 | 2 |
| 10 | 7 | 3 | 21 | 20 | 76 | 7 | 8 |
| 11 | 9 | 8 | 39 | 24 | 106 | 2 | 5 |
| 12 | 17 | 3 | 53 | 34 | 145 | 5 | 7 |
| 13 | 18 | 7 | 36 | 41 | 148 | 7 | 21 |
| 14 | 22 | 21 | 41 | 42 | 177 | 15 | 25 |
| 15 | 15 | 21 | 51 | 60 | 204 | 13 | 28 |
| 16 | 18 | 20 | 67 | 84 | 219 | 14 | 14 |
| 17 | 23 | 26 | 61 | 81 | 262 | 17 | 44 |
| 18 | 33 | 35 | 70 | 89 | 345 | 18 | 46 |
| 19 | 41 | 38 | 95 | 99 | 309 | 27 | 57 |
| 20 | 37 | 38 | 93 | 91 | 357 | 25 | 53 |
| 21 | 37 | 41 | 113 | 112 | 422 | 23 | 66 |
| 22 | 54 | 49 | 111 | 119 | 424 | 31 | 77 |
| 23 | 61 | 62 | 130 | 163 | 548 | 25 | 99 |
| 24 | 129 | 75 | 164 | 320 | 963 | 51 | 165 |
| 25 | 99 | 82 | 112 | 196 | 718 | 53 | 140 |
| 26 | 95 | 69 | 111 | 159 | 628 | 47 | 132 |
| 27 | 113 | 74 | 114 | 168 | 636 | 62 | 143 |
| 28 | 107 | 87 | 77 | 174 | 693 | 57 | 116 |
| 29 | 88 | 66 | 43 | 171 | 633 | 48 | 139 |
| 30 | 29 | 77 | 56 | 163 | 657 | 53 | 164 |
| 31 | 11 | 49 | 28 | 45 | 286 | 35 | 93 |
| 32 | 6 | 37 | 15 | 22 | 219 | 30 | 69 |
| 33 | 6 | 26 | 11 | 11 | 128 | 14 | 58 |
| 34 | 3 | 20 | 2 | 8 | 105 | 13 | 37 |
| 35 | 2 | 18 | 4 | 3 | 77 | 13 | 14 |
| 36 | 2 | 17 | 7 | 19 | 141 | 12 | 30 |

| **Table S2: Numbers of Observations in Each Country and Survey Method** | | | | | |
| --- | --- | --- | --- | --- | --- |
| Country | Number of Observations | Survey Method | GDP in 2017 (current USD) | Population in 2017 | |
| Egypt | 1,010 | Face-to-Face-Based | 2.3537E+11 | 9.6443E+07 | |
| South Africa | 1,110 | Web-Based | 3.4955E+11 | 5.7000E+07 | |
| China | 18,931 | Web-Based | 1.2143E+13 | 1.3864E+09 | |
| India | 6,562 | Both | 2.6522E+12 | 1.3387E+09 | |
| Indonesia | 2,363 | Both | 1.0154E+12 | 2.6465E+08 | |
| Japan | 10,098 | Web-Based | 4.8600E+12 | 1.2679E+08 | |
| Kazakhstan | 1,000 | Face-to-Face-Based | 1.6681E+11 | 1.8038E+07 | |
| Malaysia | 1,077 | Web-Based | 3.1896E+11 | 3.1105E+07 | |
| Mongolia | 500 | Face-to-Face-Based | 1.1426E+10 | 3.1138E+06 | |
| Myanmar | 1,073 | Face-to-Face-Based | 6.6719E+10 | 5.3383E+07 | |
| Philippines | 1,672 | Web-Based | 3.1362E+11 | 1.0517E+08 | |
| Singapore | 550 | Web-Based | 3.3841E+11 | 5.6123E+06 | |
| Sri Lanka | 284 | Web-Based | 8.8020E+10 | 2.1444E+07 | |
| Thailand | 1,115 | Web-Based | 4.5528E+11 | 6.9210E+07 | |
| Turkey | 1,954 | Web-Based | 8.5268E+11 | 8.1102E+07 | |
| Vietnam | 1,497 | Both | 2.2378E+11 | 9.4597E+07 | |
| Czech | 1,178 | Web-Based | 2.1591E+11 | 1.0594E+07 | |
| France | 2,130 | Web-Based | 2.5863E+12 | 6.6865E+07 | |
| Germany | 3,165 | Web-Based | 3.6567E+12 | 8.2657E+07 | |
| Greece | 1,358 | Web-Based | 2.0309E+11 | 1.0755E+07 | |
| Hungary | 1,354 | Web-Based | 1.4151E+11 | 9.7880E+06 | |
| Italy | 2,106 | Web-Based | 1.9570E+12 | 6.0537E+07 | |
| Netherlands | 1,371 | Web-Based | 8.3181E+11 | 1.7131E+07 | |
| Poland | 2,218 | Web-Based | 5.2622E+11 | 3.7975E+07 | |
| Romania | 472 | Web-Based | 2.1170E+11 | 1.9587E+07 | |
| Russia | 2,118 | Web-Based | 1.5786E+12 | 1.4450E+08 | |
| Spain | 2,032 | Web-Based | 1.3093E+12 | 4.6593E+07 | |
| Sweden | 1,330 | Web-Based | 5.4055E+11 | 1.0058E+07 | |
| United Kingdom | 2,993 | Web-Based | 2.6662E+12 | 6.6059E+07 | |
| Canada | 1,332 | Web-Based | 1.6469E+12 | 3.6543E+07 | |
| Mexico | 1,669 | Web-Based | 1.1577E+12 | 1.2478E+08 | |
| United States | 10,620 | Web-Based | 1.9485E+13 | 3.2499E+08 | |
| Australia | 2,004 | Web-Based | 1.3301E+12 | 2.4602E+07 | |
| Brazil | 2,255 | Web-Based | 2.0536E+12 | 2.0783E+08 | |
| Chile | 1,174 | Web-Based | 2.7775E+11 | 1.8470E+07 | |
| Colombia | 1,089 | Web-Based | 3.1179E+11 | 4.8901E+07 | |
| Venezuela * | 807 | Face-to-Face-Based | 1.4384E+11 | 2.9390E+07 | |
| Total | 95,571 |  | 6.6924E+13 | 5.1513E+09 | |
| The proportion of the world population: 68.58% | | | | |  |
| The proportion of world GDP: 82.67% | | | | |  |

Note: Data on population and GDP are provided by World Bank. World Bank did not provide the GDP of Venezuela in 2017.

Population: <https://data.worldbank.org/indicator/sp.pop.totl>

GDP: <https://data.worldbank.org/indicator/ny.gdp.mktp.cd>

GDP of Venezuela: <https://countryeconomy.com/gdp/venezuela>

| **Table S3: Descriptive Statistics of Features** | | | | | | | |
| --- | --- | --- | --- | --- | --- | --- | --- |
| **Statistic** | **N** | **Mean** | **St. Dev.** | **Min** | **Pctl(25)** | **Pctl(75)** | **Max** |
| Mental Health Score | 89,273 | 24.319 | 6.301 | 0 | 21 | 29 | 36 |
| RI | 89,273 | 0.734 | 1.138 | 0.003 | 0.227 | 0.879 | 45.664 |
| Social Class | 89,273 | 2.960 | 0.822 | 1 | 3 | 3 | 5 |
| Student Dummy | 89,273 | 0.056 | 0.231 | 0 | 0 | 0 | 1 |
| Worker Dummy | 89,273 | 0.533 | 0.499 | 0 | 0 | 1 | 1 |
| Company Owner Dummy | 89,273 | 0.021 | 0.142 | 0 | 0 | 0 | 1 |
| Government Officer Dummy | 89,273 | 0.031 | 0.174 | 0 | 0 | 0 | 1 |
| Self-employed Dummy | 89,273 | 0.076 | 0.265 | 0 | 0 | 0 | 1 |
| Professional Job Dummy | 89,273 | 0.035 | 0.183 | 0 | 0 | 0 | 1 |
| Housewife Dummy | 89,273 | 0.084 | 0.277 | 0 | 0 | 0 | 1 |
| Unemployed Dummy | 89,273 | 0.086 | 0.280 | 0 | 0 | 0 | 1 |
| Pleasure | 89,273 | 3.167 | 0.809 | 1 | 3 | 4 | 4 |
| Anger | 89,273 | 2.351 | 0.935 | 1 | 2 | 3 | 4 |
| Sadness | 89,273 | 2.347 | 0.954 | 1 | 2 | 3 | 4 |
| Enjoyment | 89,273 | 3.052 | 0.830 | 1 | 3 | 4 | 4 |
| Smile | 89,273 | 3.318 | 0.777 | 1 | 3 | 4 | 4 |
| Euthusiastic | 89,273 | 0.423 | 0.494 | 0 | 0 | 1 | 1 |
| Critical | 89,273 | 0.168 | 0.374 | 0 | 0 | 0 | 1 |
| Dependable | 89,273 | 0.648 | 0.478 | 0 | 0 | 1 | 1 |
| Anxious | 89,273 | 0.245 | 0.430 | 0 | 0 | 0 | 1 |
| Open to New Experience | 89,273 | 0.507 | 0.500 | 0 | 0 | 1 | 1 |
| Reserved | 89,273 | 0.430 | 0.495 | 0 | 0 | 1 | 1 |
| Sympathetic | 89,273 | 0.623 | 0.485 | 0 | 0 | 1 | 1 |
| Careless | 89,273 | 0.119 | 0.324 | 0 | 0 | 0 | 1 |
| Calm | 89,273 | 0.499 | 0.500 | 0 | 0 | 1 | 1 |
| Uncreative | 89,273 | 0.177 | 0.382 | 0 | 0 | 0 | 1 |
| Urban Center Dummy | 89,273 | 0.688 | 0.463 | 0 | 0 | 1 | 1 |
| Urban Area Dummy | 89,273 | 0.144 | 0.352 | 0 | 0 | 0 | 1 |
| Rural Area Dummy | 89,273 | 0.167 | 0.373 | 0 | 0 | 0 | 1 |
| Income Group | 89,273 | 2.795 | 0.900 | 1 | 2 | 3 | 5 |
| Female Dummy | 89,273 | 0.489 | 0.500 | 0 | 0 | 1 | 1 |
| Age | 89,273 | 42.797 | 14.817 | 18 | 30 | 54 | 99 |
| Self-reported Health | 89,273 | 3.824 | 0.883 | 1 | 3 | 4 | 5 |
| Bachelor Dummy | 89,273 | 0.390 | 0.488 | 0 | 0 | 1 | 1 |
| Master Dummy | 89,273 | 0.090 | 0.287 | 0 | 0 | 0 | 1 |
| PhD Dummy | 89,273 | 0.018 | 0.134 | 0 | 0 | 0 | 1 |
| Community Livable | 89,273 | 4.023 | 0.852 | 1 | 4 | 5 | 5 |
| Community Attachment | 89,273 | 3.621 | 1.038 | 1 | 3 | 4 | 5 |
| Community Safety | 89,273 | 3.017 | 0.748 | 0 | 3 | 3 | 4 |
| Children Number | 89,273 | 1.212 | 1.216 | 0 | 0 | 2 | 10 |
| Cropland (%) | 89,273 | 13.668 | 19.065 | 0.000 | 0.996 | 18.257 | 99.804 |
| Forest (%) | 89,273 | 12.482 | 19.495 | 0.000 | 0.513 | 15.169 | 100.000 |
| Grassland (%) | 89,273 | 10.469 | 14.771 | 0.000 | 1.057 | 13.935 | 99.316 |
| Shrubland (%) | 89,273 | 1.081 | 3.876 | 0.000 | 0.000 | 0.363 | 40.000 |
| Wetland (%) | 89,273 | 0.083 | 0.268 | 0.000 | 0.000 | 0.047 | 3.000 |
| Water (%) | 89,273 | 3.391 | 8.050 | 0.000 | 0.017 | 2.292 | 50.000 |
| Urban Land (%) | 89,273 | 57.991 | 33.761 | 0.000 | 29.081 | 88.476 | 100.000 |
| Bare Land (%) | 89,273 | 0.525 | 2.159 | 0.000 | 0.000 | 0.168 | 20.000 |
| X | 89,273 | 47.756 | 79.521 | -128.6 | 1.025 | 116.397 | 153.555 |
| Y | 89,273 | 29.558 | 20.730 | -53.3 | 21.029 | 42.002 | 69.558 |

| **Table S4: Descriptions of Features** | | |
| --- | --- | --- |
| **Aspect** | **Predictor Name** | **Descriptions or Questions of Predictors** |
|  | Mental Health Score | The output variable |
| Income | RI | Because of the economic gap between countries, absolute annual individual income is less effective in classifying the level of human mental health among people from different countries. Therefore, we employ a new variable RI. |
| Social Class | Social Class | Which **social class** do you feel that you belong within your country? (5: Upper - 1: Lower) |
| Job | Student Dummy | Are you a student, now? (1: yes, 0: otherwise) |
| Job | Worker Dummy | Are you a worker including both full-time and part-time, now? (1: yes, 0: otherwise) |
| Job | Company Owner Dummy | Are you a company owner, now? (1: yes, 0: otherwise) |
| Job | Government Officer Dummy | Are you a government officer, now? (1: yes, 0: otherwise) |
| Job | Self-employed Dummy | Are you self-employed, now? (1: yes, 0: otherwise) |
| Job | Professional Job Dummy | Are you doing a profesional job, such as professor, lawyer, doctor, now? (1: yes, 0: otherwise) |
| Job | Housewife Dummy | Are you a housewife or househusband, now? (1: yes, 0: otherwise) |
| Job | Unemployed Dummy | Are you unemployed, now? (1: yes, 0: otherwise) |
| Emotion Weekly | Pleasure | How often have you felt or experienced the following feelings or actions within a week? (4: often - 1:not at all) |
| Emotion Weekly | Anger | How often have you felt or experienced the following feelings or actions within a week? (4: often - 1:not at all) |
| Emotion Weekly | Sadness | How often have you felt or experienced the following feelings or actions within a week? (4: often - 1:not at all) |
| Emotion Weekly | Enjoyment | How often have you felt or experienced the following feelings or actions within a week? (4: often - 1:not at all) |
| Emotion Weekly | Smile | How often have you felt or experienced the following feelings or actions within a week? (4: often - 1:not at all) |
| Personality | Euthusiastic | Do you see yourself as someone who is euthusiatic? (1: yes - 0: otherwise) |
| Personality | Critical | Do you see yourself as someone who is critical? (1: yes - 0: otherwise) |
| Personality | Dependable | Do you see yourself as someone who is dependable? (1: yes - 0: otherwise) |
| Personality | Anxious | Do you see yourself as someone who is anxious? (1: yes - 0: otherwise) |
| Personality | Open to New Experience | Do you see yourself as someone who opens to new experience? (1: yes - 0: otherwise) |
| Personality | Reserved | Do you see yourself as someone who is reserved? (1: yes - 0: otherwise) |
| Personality | Sympathetic | Do you see yourself as someone who is sympathetic? (1: yes - 0: otherwise) |
| Personality | Careless | Do you see yourself as someone who is disorganized? (1: yes - 0: otherwise) |
| Personality | Calm | Do you see yourself as someone who is calm? (1: yes - 0: otherwise) |
| Personality | Uncreative | Do you see yourself as someone who is conventional? (1: yes - 0: otherwise) |
| Geographical variable | Urban Center Dummy | The data are extracted from ESA. Data source (European Commission): https://ghsl.jrc.ec.europa.eu/datasets.php |
| Geographical variable | Urban Area Dummy | The data are extracted from ESA. Data source (European Commission): https://ghsl.jrc.ec.europa.eu/datasets.php |
| Geographical variable | Rural Area Dummy | The data are extracted from ESA. Data source (European Commission): https://ghsl.jrc.ec.europa.eu/datasets.php |
| Income Group | Income Group | Which **income group** do you feel that you belong within your country? (5: Upper - 1: Lower) |
| Gender | Female Dummy | Are you female? (1: yes, 0: otherwise) |
| Age | Age | Please tell us your age. |
| Physical Health | Self-reported Health | All in all, how would you describe your state of health? (5: very good - 1: very poor) |
| Education | Bachelor Dummy | Are you with bachelor degree? (1: yes, 0: otherwise) |
| Education | Master Dummy | Are you with master degree? (1: yes, 0: otherwise) |
| Education | PhD Dummy | Are you with Ph.D. degree? (1: yes, 0: otherwise) |
| Living Environmental Aspect | Community Liviability | How livable is your neighborhood? (5: very livable - 1: not livable) |
| Living Environmental Aspect | Community Attachment | How attached are you to your local community? (4: extremly attached - 1: completely detached) |
| Living Environmental Aspect | Community Safety | Please tell us about safety of your neighborhood. (4: very safe - 1: very dangerous) |
| Family Aspect | Children Number | How many children do you have? |
| Land Cover | Cropland (%) | Percentage of a certain land type. Data Source: http://data.ess.tsinghua.edu.cn/ |
| Land Cover | Forest (%) | Percentage of a certain land type. Data Source: http://data.ess.tsinghua.edu.cn/ |
| Land Cover | Grassland (%) | Percentage of a certain land type. Data Source: http://data.ess.tsinghua.edu.cn/ |
| Land Cover | Shrubland (%) | Percentage of a certain land type. Data Source: http://data.ess.tsinghua.edu.cn/ |
| Land Cover | Wetland (%) | Percentage of a certain land type. Data Source: http://data.ess.tsinghua.edu.cn/ |
| Land Cover | Water (%) | Percentage of a certain land type. Data Source: http://data.ess.tsinghua.edu.cn/ |
| Land Cover | Urban Land (%) | Percentage of a certain land type. Data Source: http://data.ess.tsinghua.edu.cn/ |
| Land Cover | Bare Land (%) | Percentage of a certain land type. Data Source: http://data.ess.tsinghua.edu.cn/ |
| **Note: The features that do not mention the data source are from our surveys.** | | |

| **Table S5: Hyperparameter Fine-tuning** | | | | | | | |
| --- | --- | --- | --- | --- | --- | --- | --- |
| Ntree | | Nfeature | | Nfeature | | Nremain | |
| Potential Parameter | R2 based on 10-fold CV | Potential Parameter | R2 based on 10-fold CV | Potential Parameter | R2 based on 10-fold CV | Potential Parameter | R2 based on 10-fold CV |
| 50 | 37.838% | 1 | 35.626% | 26 | 40.355% | 2 | 41.139% |
| 100 | 38.620% | 2 | 38.389% | 27 | 40.302% | 5 | 41.102% |
| 200 | 38.974% | 3 | 39.783% | 28 | 40.278% | 10 | 41.033% |
| 300 | 39.121% | 4 | 40.528% | 29 | 40.207% | 15 | 40.974% |
| 400 | 39.197% | 5 | 40.822% | 30 | 40.186% | 20 | 40.922% |
| 500 | 39.238% | 6 | 41.052% | 31 | 40.155% | 25 | 40.842% |
| **1000** | **39.284%** | 7 | 41.114% | 32 | 40.114% | **30** | **40.753%** |
|  |  | 8 | 41.124% | 33 | 40.052% | 35 | 40.702% |
|  |  | 9 | 41.136% | 34 | 40.012% | 40 | 40.659% |
|  |  | 10 | 41.138% | 35 | 39.968% |  |  |
|  |  | **11** | **41.139%** | 36 | 39.974% |  |  |
|  |  | 12 | 41.076% | 37 | 39.916% |  |  |
|  |  | 13 | 41.059% | 38 | 39.811% |  |  |
|  |  | 14 | 40.989% | 39 | 39.788% |  |  |
|  |  | 15 | 40.930% | 40 | 39.787% |  |  |
|  |  | 16 | 40.876% | 41 | 39.712% |  |  |
|  |  | 17 | 40.814% | 42 | 39.710% |  |  |
|  |  | 18 | 40.777% | 43 | 39.597% |  |  |
|  |  | 19 | 40.738% | 44 | 39.558% |  |  |
|  |  | 20 | 40.670% | 45 | 39.503% |  |  |
|  |  | 21 | 40.634% | 46 | 39.500% |  |  |
|  |  | 22 | 40.600% | 47 | 39.381% |  |  |
|  |  | 23 | 40.542% | 48 | 39.338% |  |  |
|  |  | 24 | 40.481% | 49 | 39.284% |  |  |
|  |  | 25 | 40.420% |  |  |  |  |

# Figure:


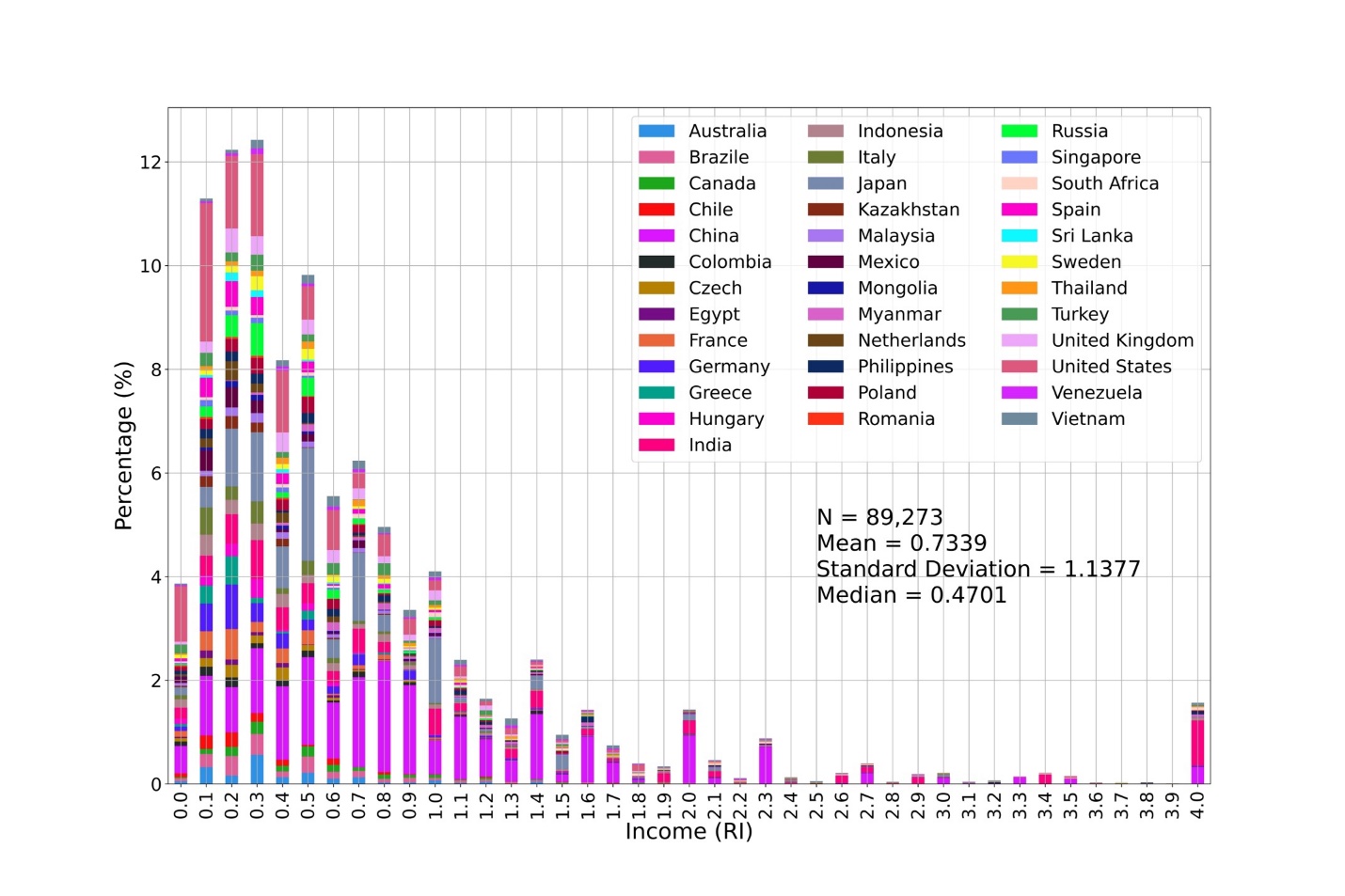


Figure S1: The Statistical Distribution of RI

(Note: In this figure, the column of 4.0 represents the count of RI no less than 4.0.)


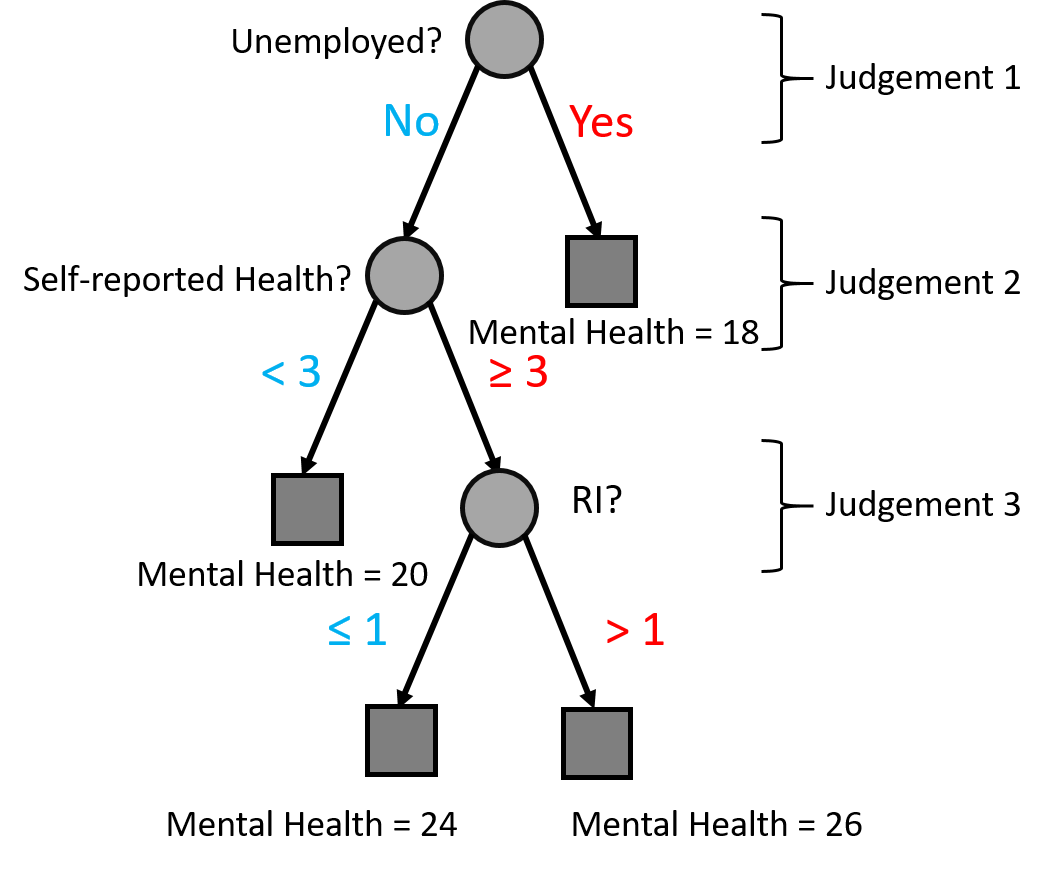


Figure S2: Example of a Regression Decision Tree


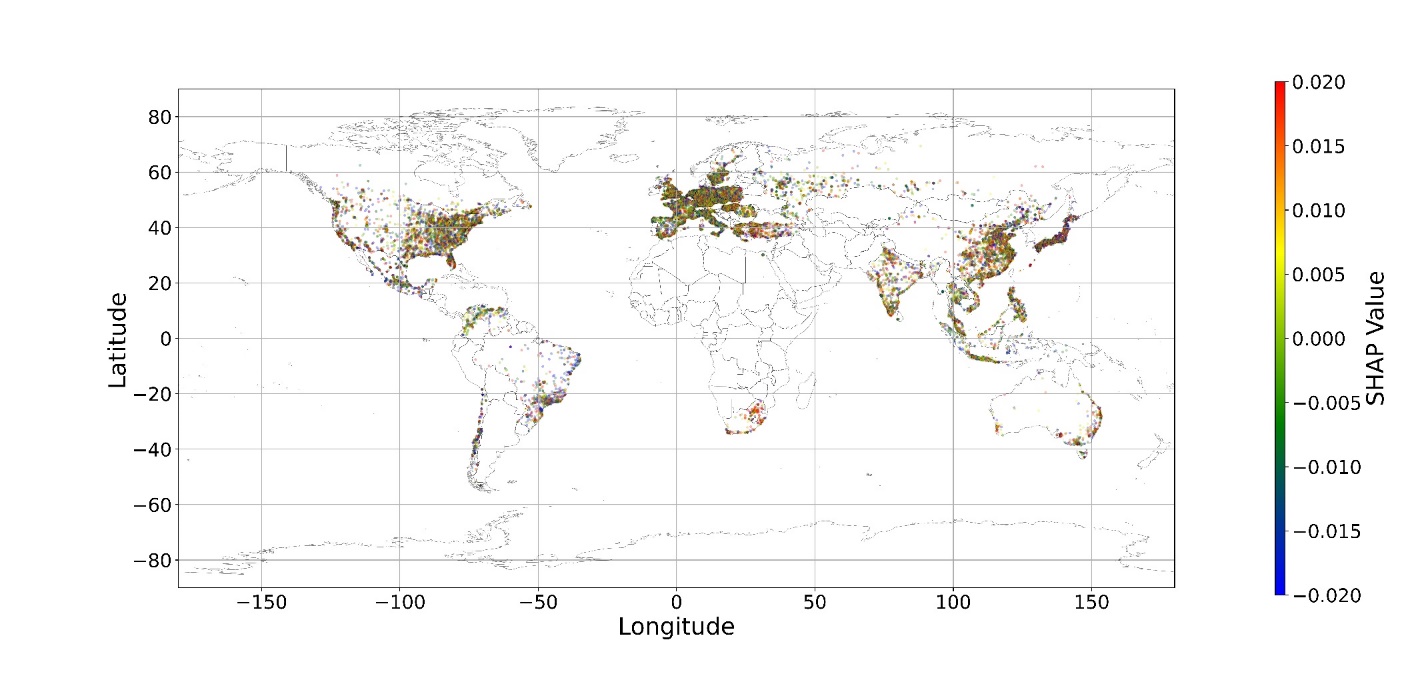


Figure S3.a: The Spatial Scatter Plot of the Income’s SHAP Values

(Map’s Shapefile is downloaded from <https://hub.arcgis.com/datasets/esri::world-countries-generalized/explore> ; We use Python 3.9.16 to plot <https://www.python.org/downloads/release/python-3916/>)


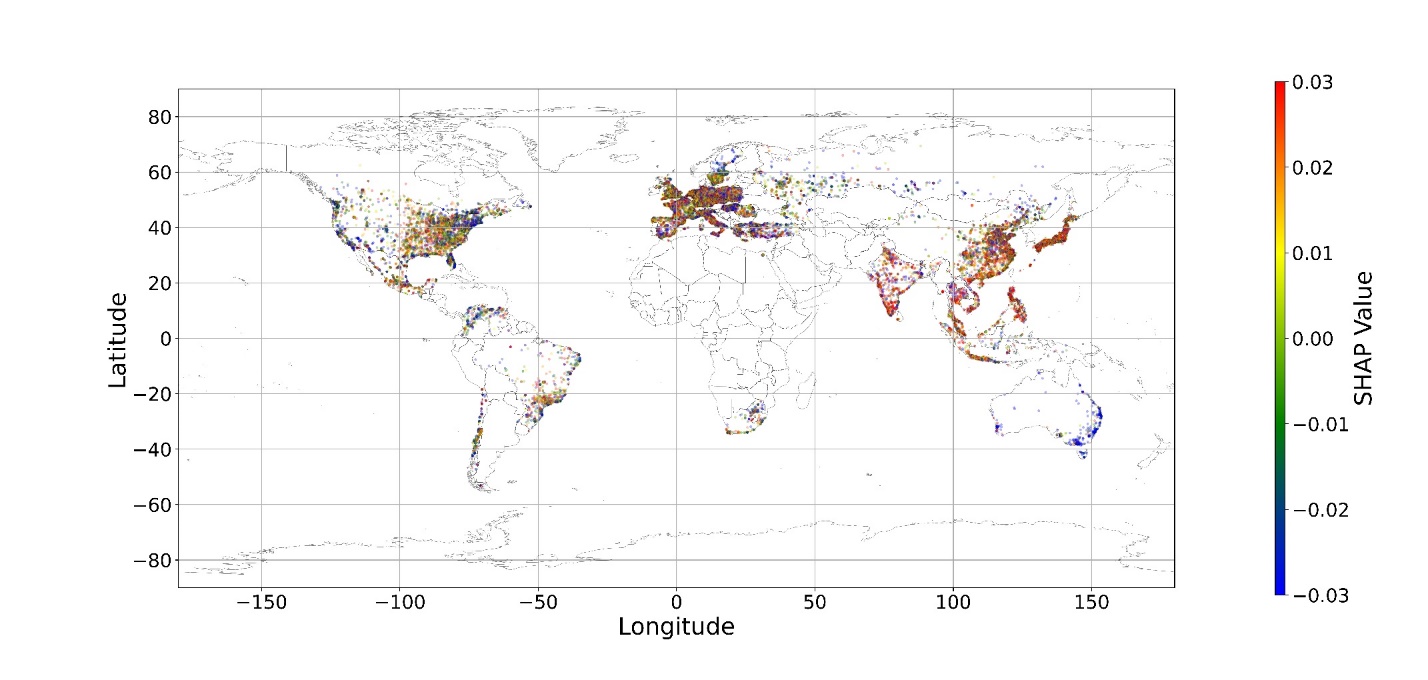


Figure S3.b: The Spatial Scatter Plot of the Cropland’s SHAP Values

(Map’s Shapefile is downloaded from <https://hub.arcgis.com/datasets/esri::world-countries-generalized/explore> ; We use Python 3.9.16 to plot <https://www.python.org/downloads/release/python-3916/>)


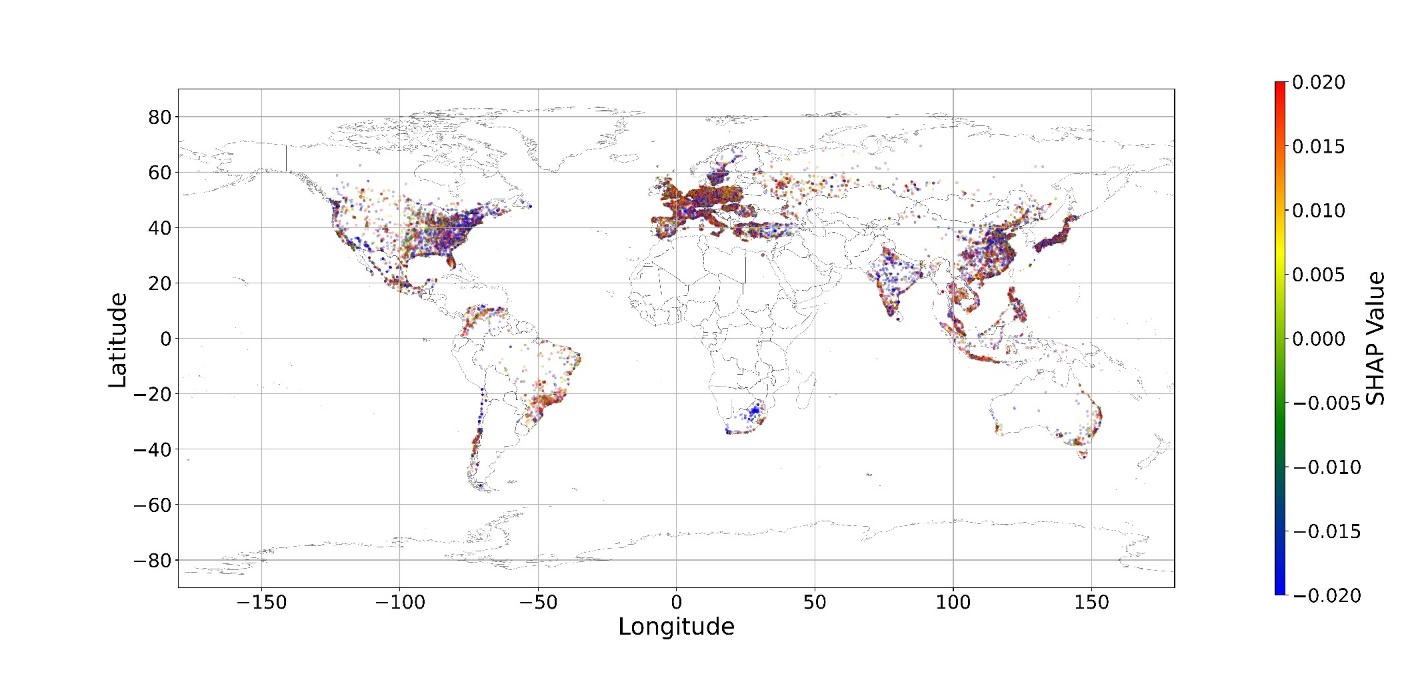


Figure S3.c: The Spatial Scatter Plot of the Forest’s SHAP Values

(Map’s Shapefile is downloaded from <https://hub.arcgis.com/datasets/esri::world-countries-generalized/explore> ; We use Python 3.9.16 to plot <https://www.python.org/downloads/release/python-3916/>)


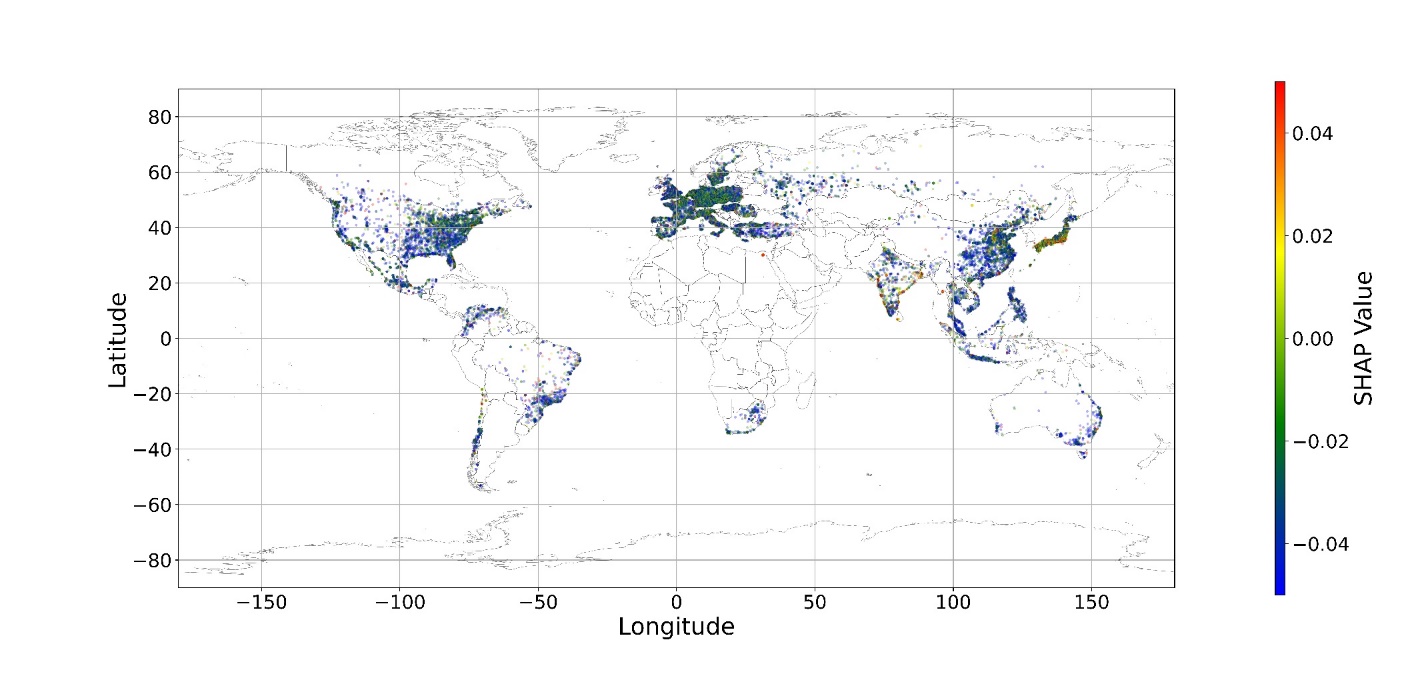


Figure S3.d: The Spatial Scatter Plot of the Grassland’s SHAP Values

(Map’s Shapefile is downloaded from <https://hub.arcgis.com/datasets/esri::world-countries-generalized/explore> ; We use Python 3.9.16 to plot <https://www.python.org/downloads/release/python-3916/>)


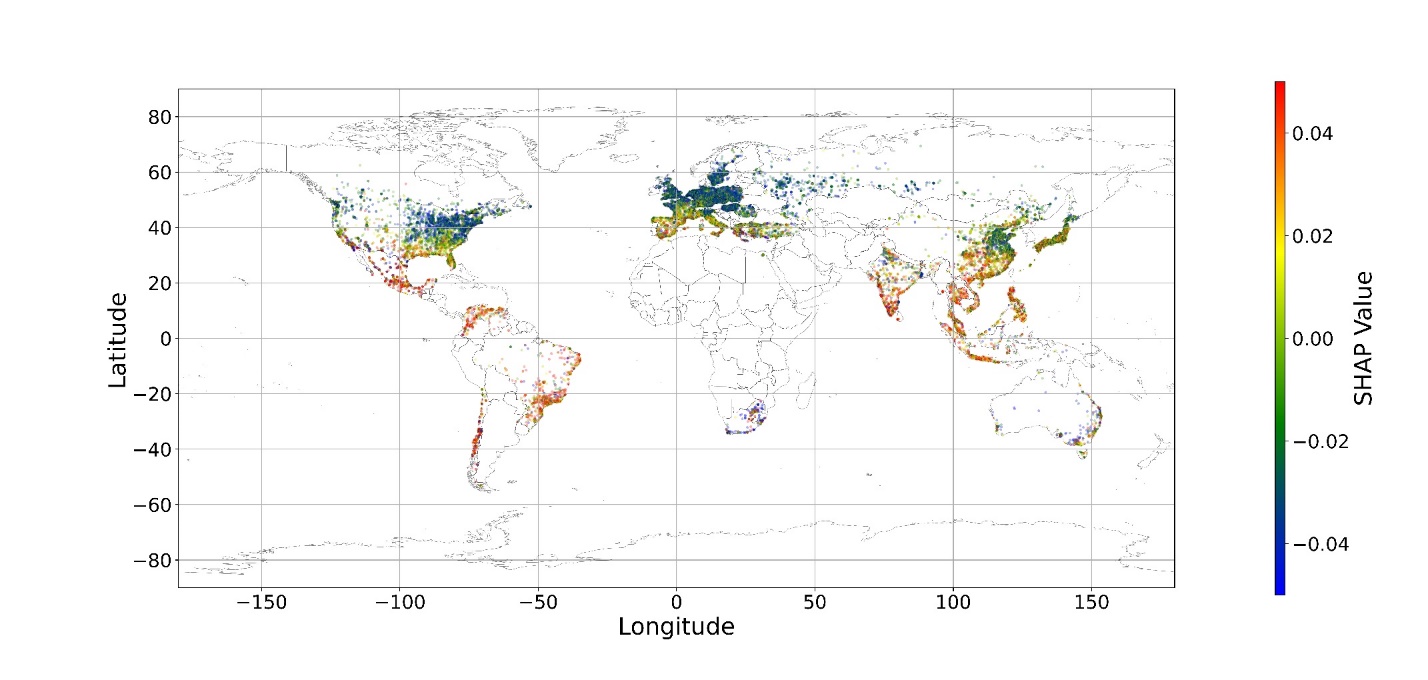


Figure S3.e: The Spatial Scatter Plot of the Shrubland’s SHAP Values

(Map’s Shapefile is downloaded from <https://hub.arcgis.com/datasets/esri::world-countries-generalized/explore> ; We use Python 3.9.16 to plot <https://www.python.org/downloads/release/python-3916/>)


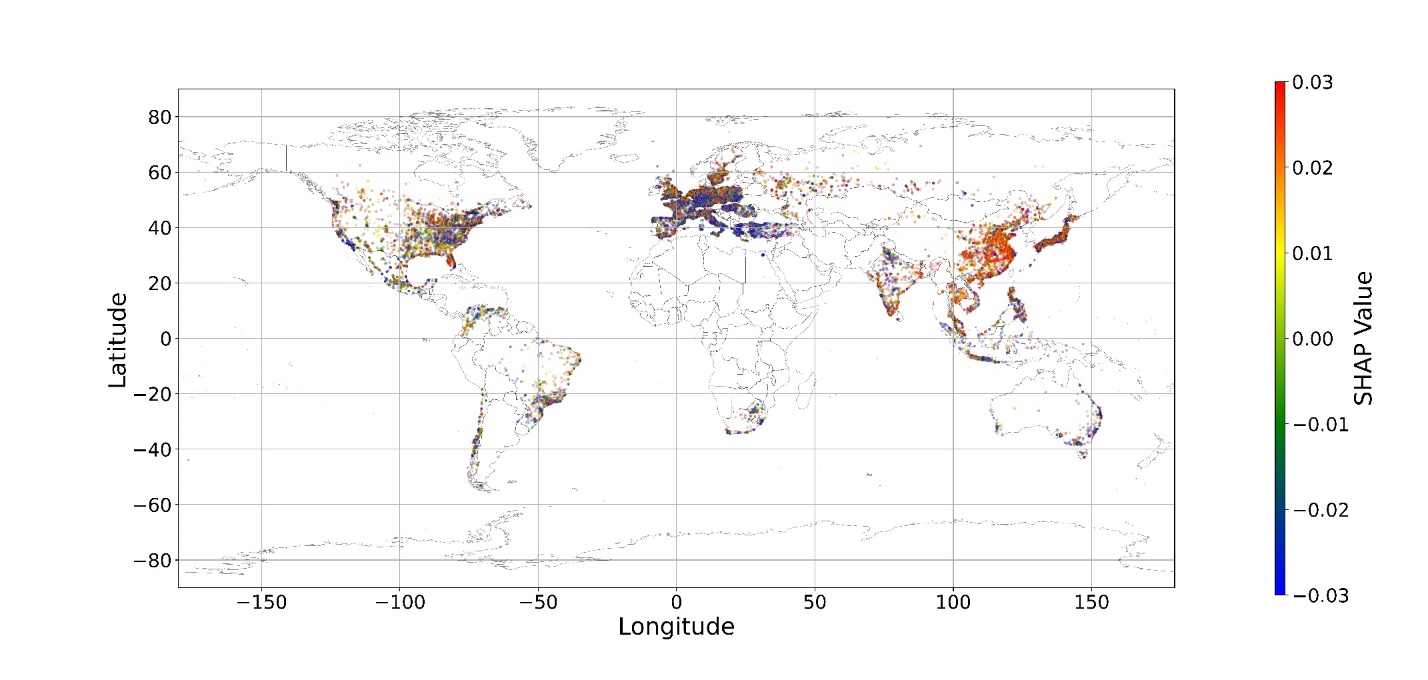


Figure S3.f: The Spatial Scatter Plot of the Water’s SHAP Values

(Map’s Shapefile is downloaded from <https://hub.arcgis.com/datasets/esri::world-countries-generalized/explore> ; We use Python 3.9.16 to plot <https://www.python.org/downloads/release/python-3916/>)


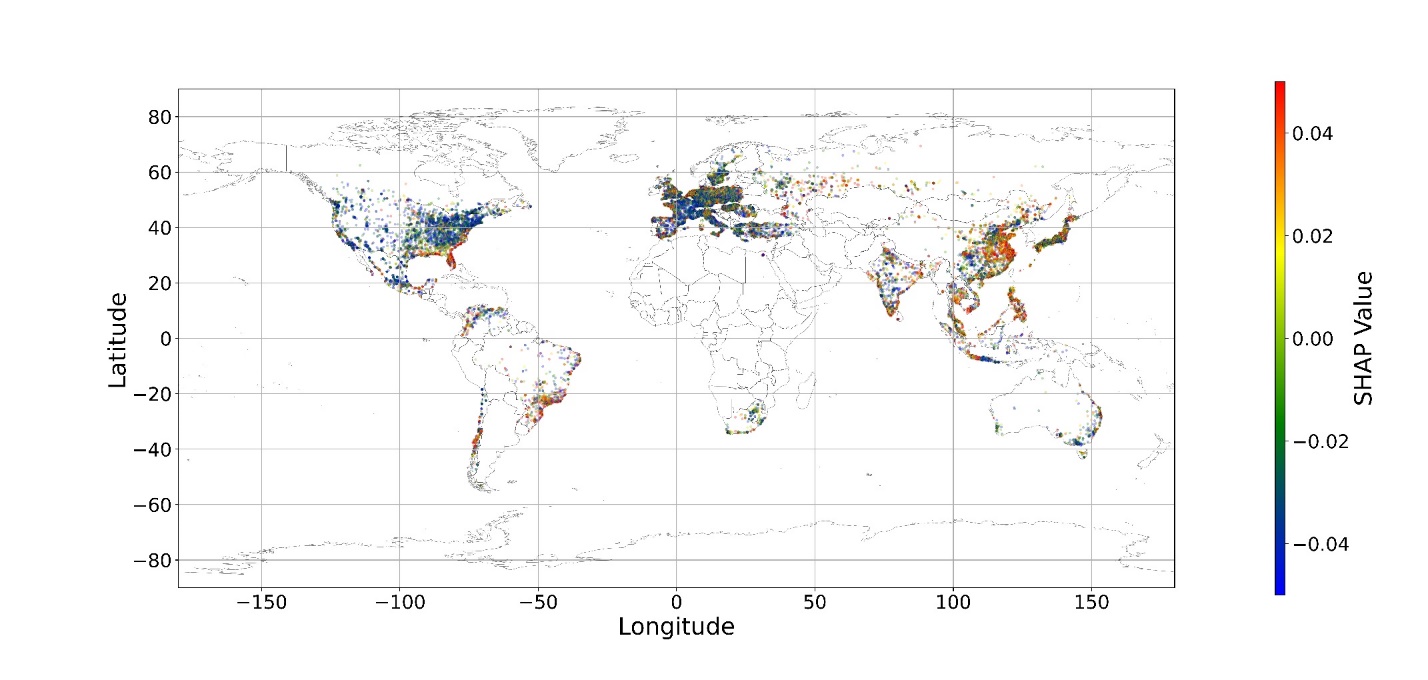


Figure S3.g: The Spatial Scatter Plot of the Wetland’s SHAP Values

(Map’s Shapefile is downloaded from <https://hub.arcgis.com/datasets/esri::world-countries-generalized/explore> ; We use Python 3.9.16 to plot <https://www.python.org/downloads/release/python-3916/>)


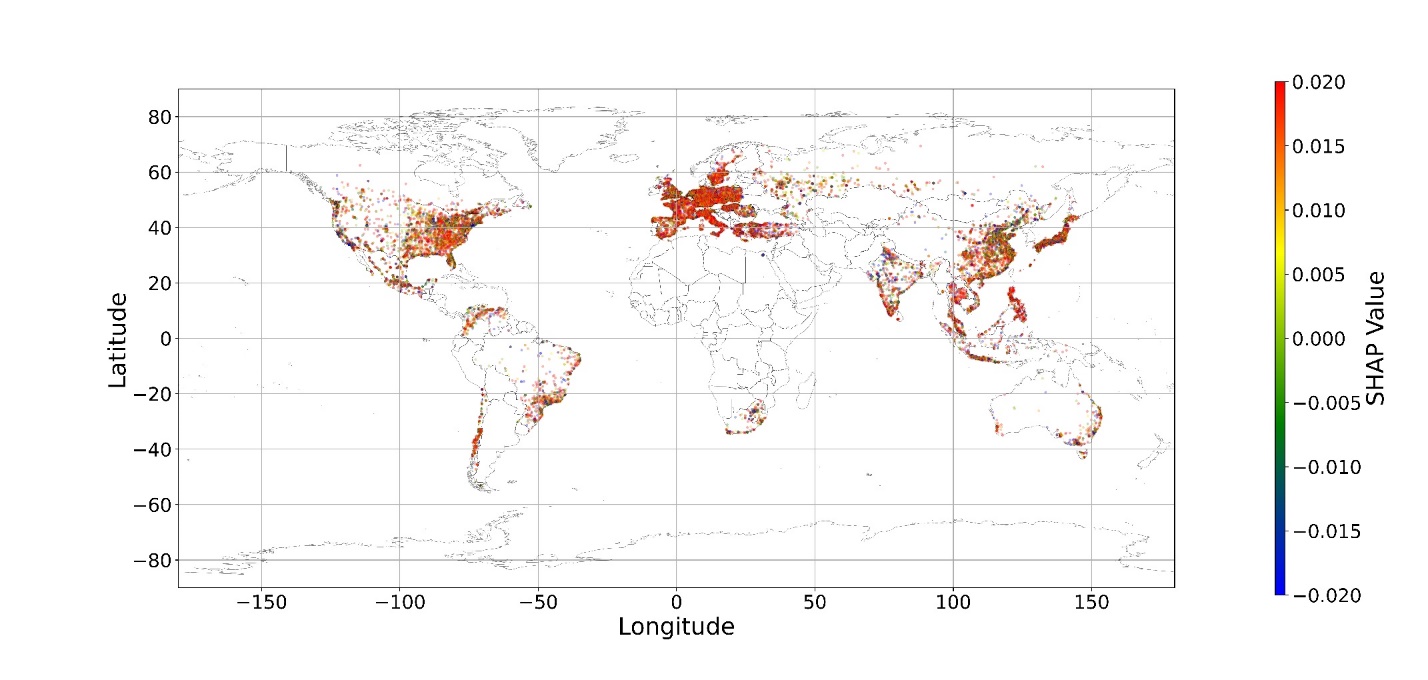


Figure S3.h: The Spatial Scatter Plot of the Urban Land’s SHAP Values

(Map’s Shapefile is downloaded from <https://hub.arcgis.com/datasets/esri::world-countries-generalized/explore> ; We use Python 3.9.16 to plot <https://www.python.org/downloads/release/python-3916/>)


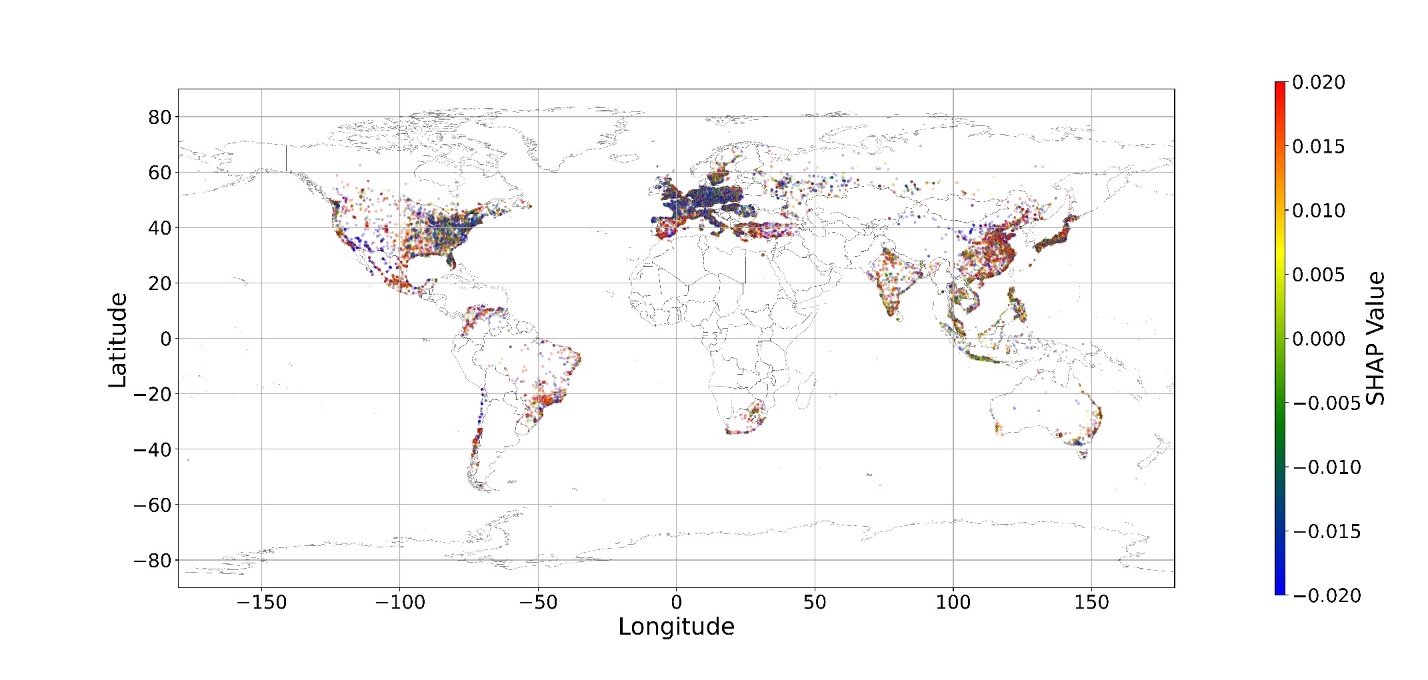


Figure S3.i: The Spatial Scatter Plot of the Bare Land’s SHAP Values

(Map’s Shapefile is downloaded from <https://hub.arcgis.com/datasets/esri::world-countries-generalized/explore> ; We use Python 3.9.16 to plot <https://www.python.org/downloads/release/python-3916/>)


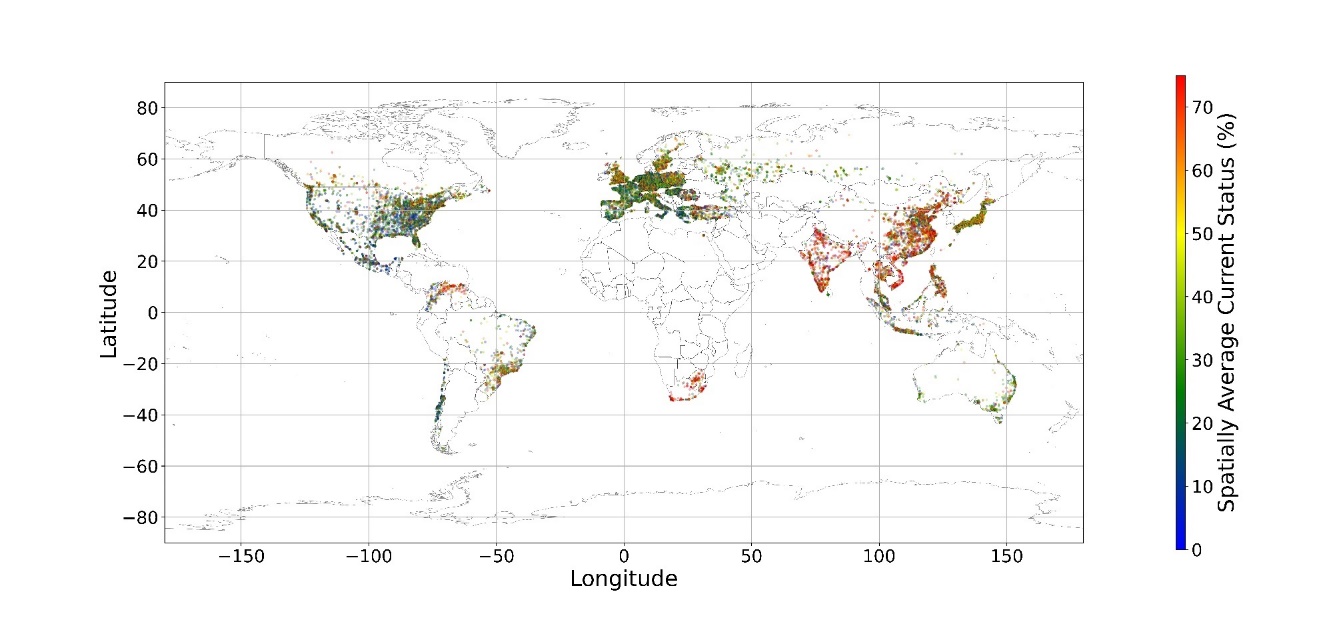


Figure S4.a: The Spatial Scatter Plot of the Income’s Current Status

(Map’s Shapefile is downloaded from <https://hub.arcgis.com/datasets/esri::world-countries-generalized/explore> ; We use Python 3.9.16 to plot <https://www.python.org/downloads/release/python-3916/>)


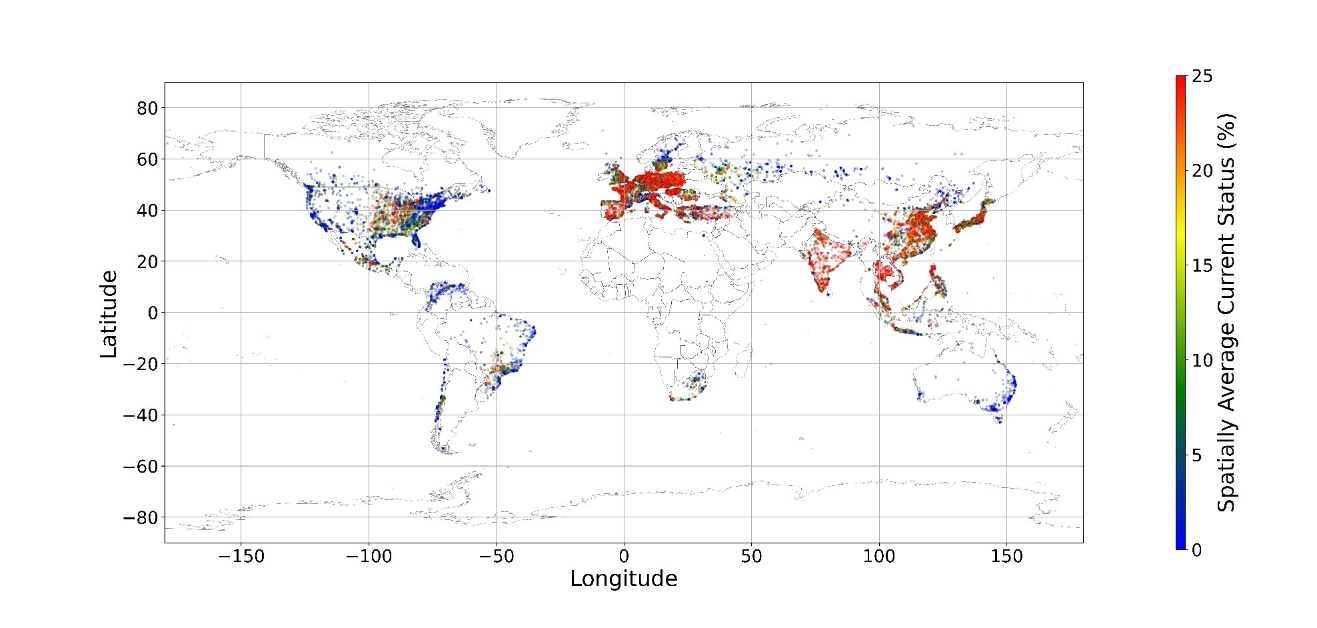


Figure S4.b: The Spatial Scatter Plot of the Cropland’s Current Status

(Map’s Shapefile is downloaded from <https://hub.arcgis.com/datasets/esri::world-countries-generalized/explore> ; We use Python 3.9.16 to plot <https://www.python.org/downloads/release/python-3916/>)


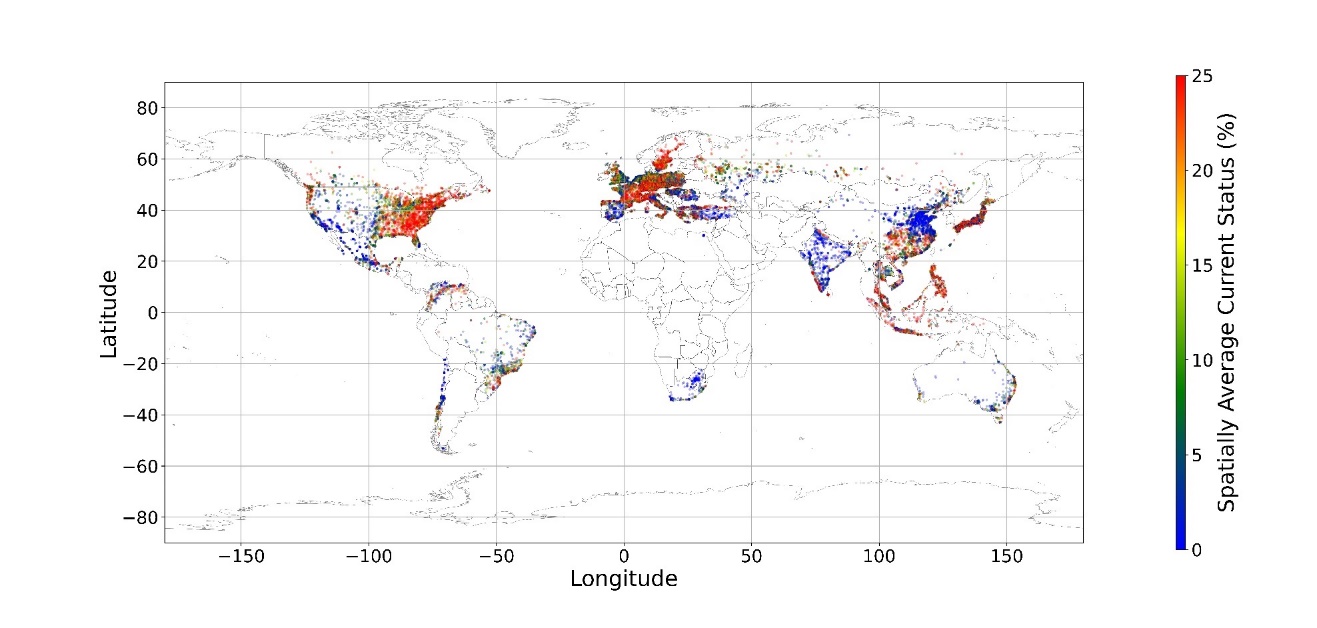


Figure S4.c: The Spatial Scatter Plot of the Forest’s Current Status

(Map’s Shapefile is downloaded from <https://hub.arcgis.com/datasets/esri::world-countries-generalized/explore> ; We use Python 3.9.16 to plot <https://www.python.org/downloads/release/python-3916/>)


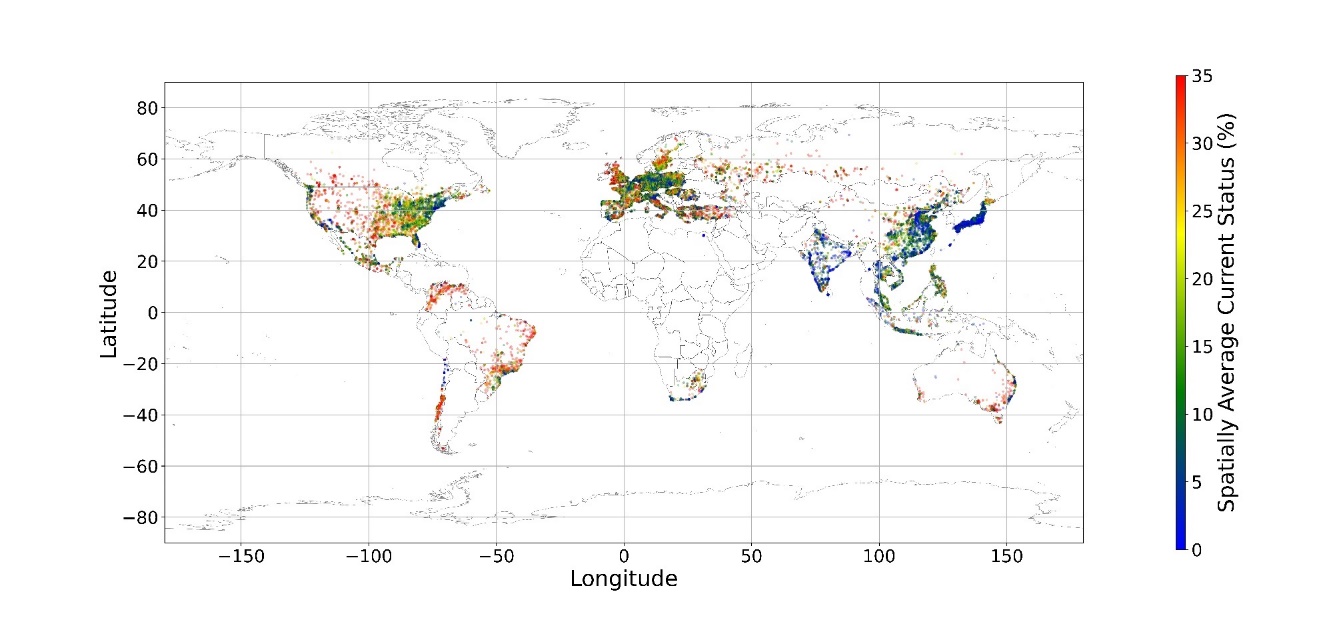


Figure S4.d: The Spatial Scatter Plot of the Grassland’s Current Status

(Map’s Shapefile is downloaded from <https://hub.arcgis.com/datasets/esri::world-countries-generalized/explore> ; We use Python 3.9.16 to plot <https://www.python.org/downloads/release/python-3916/>)


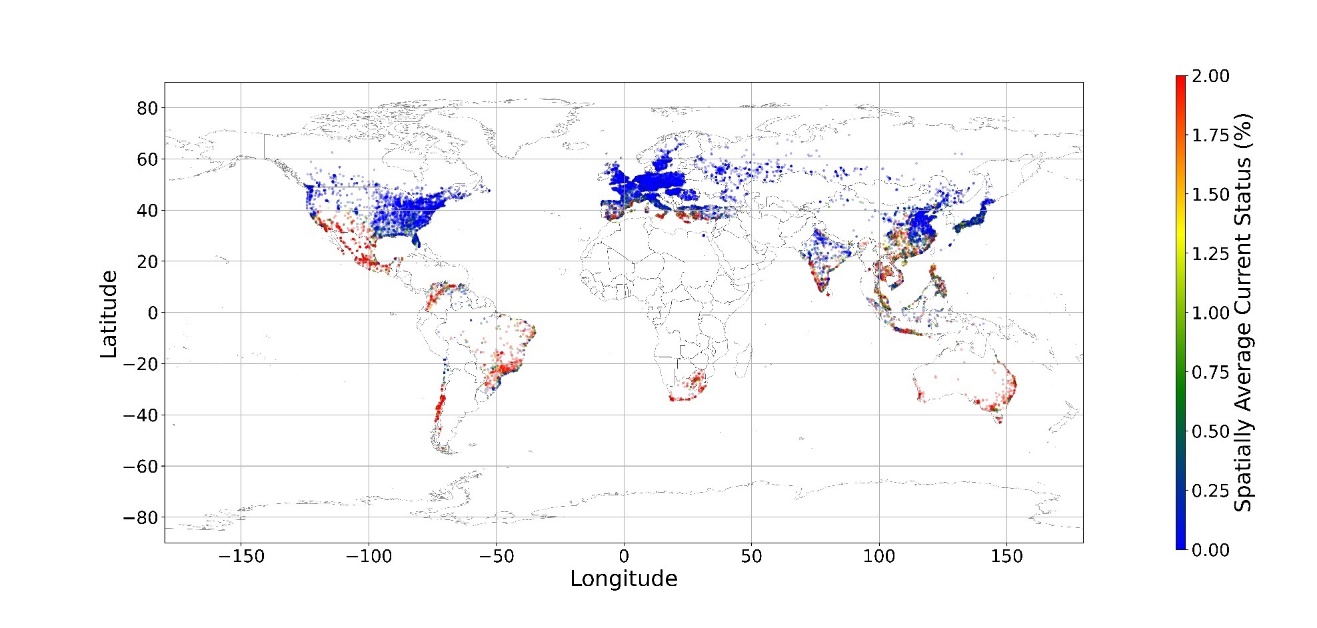


Figure S4.e: The Spatial Scatter Plot of the Shrubland’s Current Status

(Map’s Shapefile is downloaded from <https://hub.arcgis.com/datasets/esri::world-countries-generalized/explore> ; We use Python 3.9.16 to plot <https://www.python.org/downloads/release/python-3916/>)


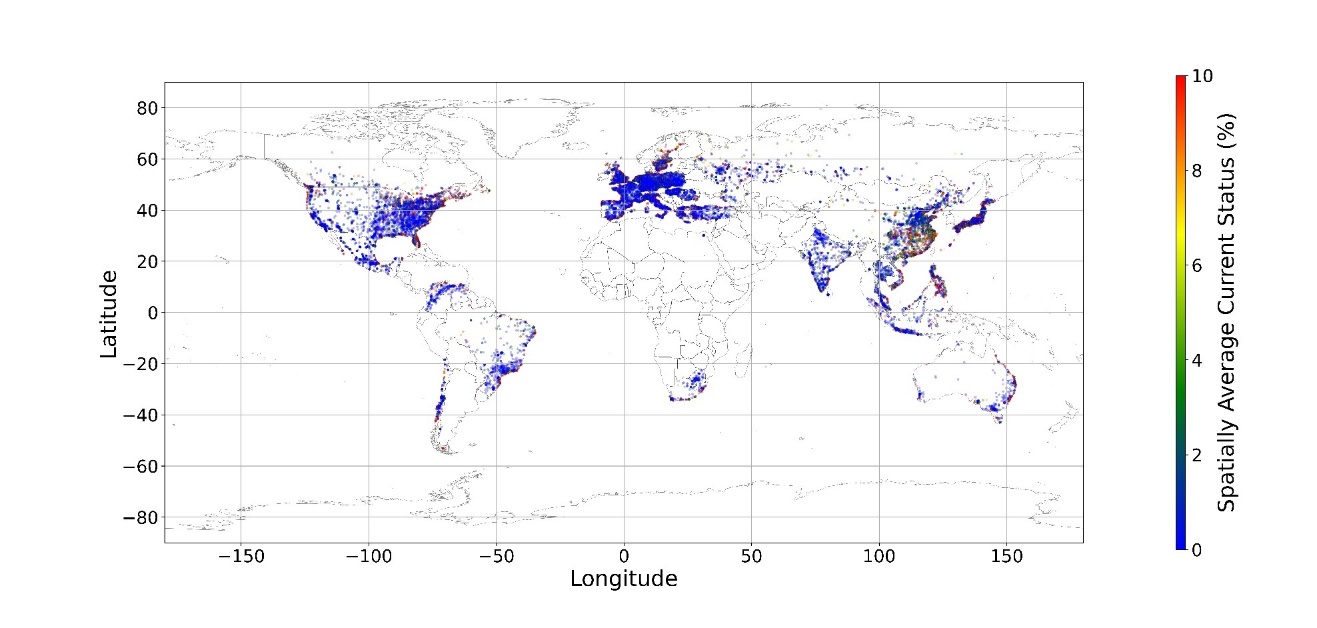


Figure S4.f: The Spatial Scatter Plot of the Water’s Current Status

(Map’s Shapefile is downloaded from <https://hub.arcgis.com/datasets/esri::world-countries-generalized/explore> ; We use Python 3.9.16 to plot <https://www.python.org/downloads/release/python-3916/>)


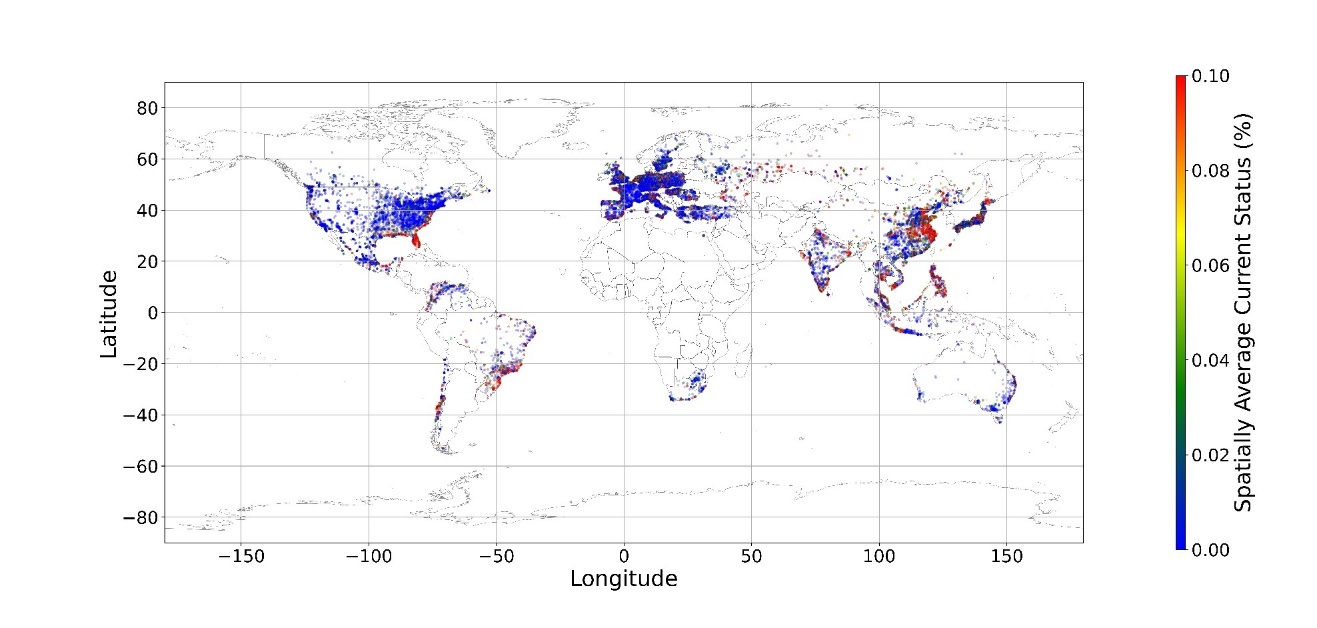


Figure S4.g: The Spatial Scatter Plot of the Wetland’s Current Status

(Map’s Shapefile is downloaded from <https://hub.arcgis.com/datasets/esri::world-countries-generalized/explore> ; We use Python 3.9.16 to plot <https://www.python.org/downloads/release/python-3916/>)


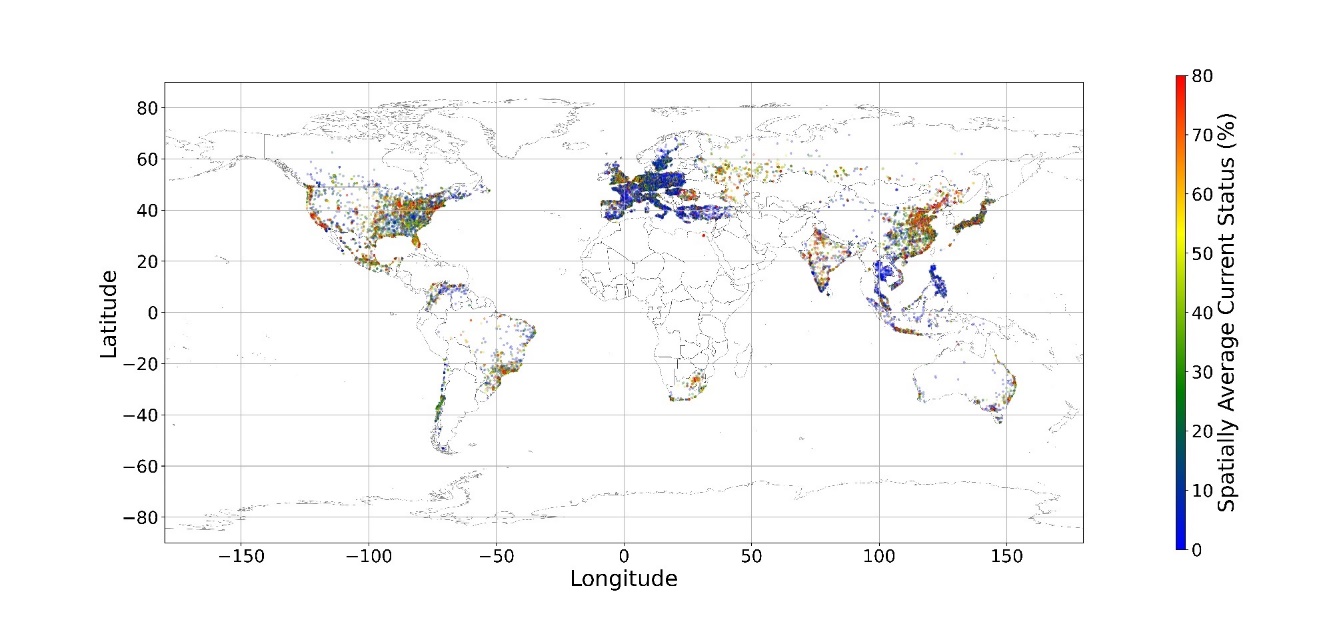


Figure S4.h: The Spatial Scatter Plot of the Urban Land’s Current Status

(Map’s Shapefile is downloaded from <https://hub.arcgis.com/datasets/esri::world-countries-generalized/explore> ; We use Python 3.9.16 to plot <https://www.python.org/downloads/release/python-3916/>)


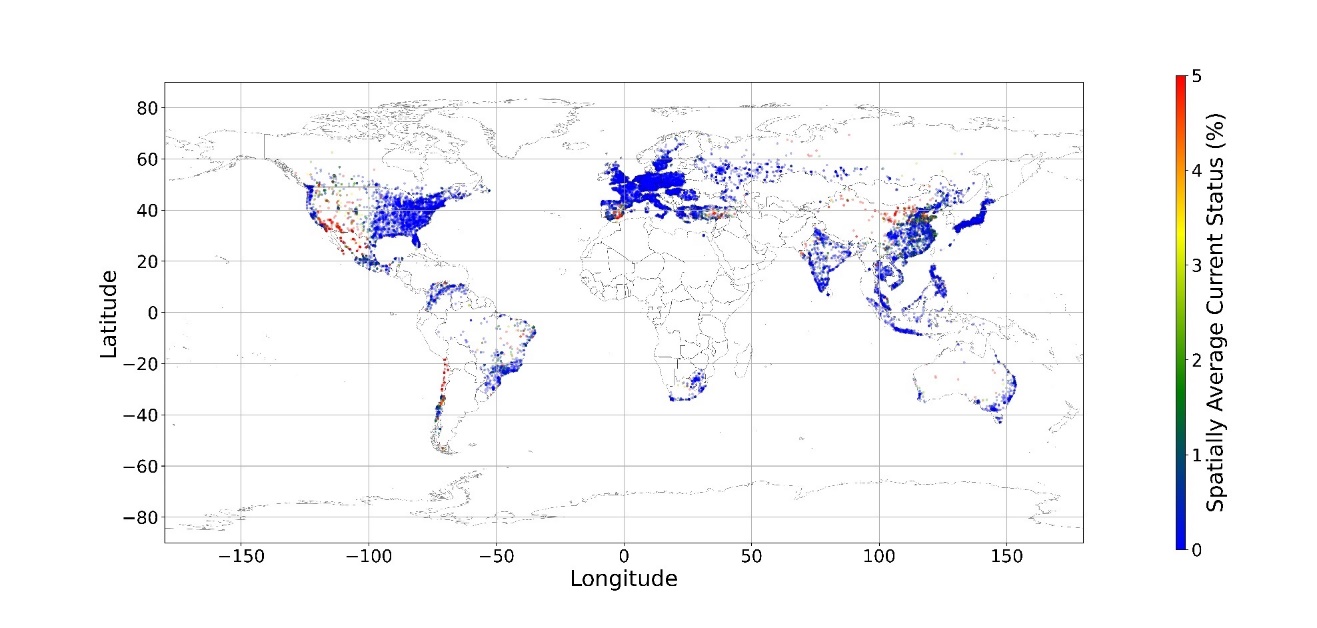


Figure S4.i: The Spatial Scatter Plot of the Bare Land’s Current Status

(Map’s Shapefile is downloaded from <https://hub.arcgis.com/datasets/esri::world-countries-generalized/explore> ; We use Python 3.9.16 to plot <https://www.python.org/downloads/release/python-3916/>)
